# Supplementary material for: BysR, a LysR-Type Pleiotropic Regulator, Controls Production of Occidiofungin by Activating the LuxR-Type Transcriptional Regulator AmbR1 in Burkholderia sp. Strain JP2-270
Source: Microbiol Spectr. 2023 Mar 20;11(2):e02684-22. doi: 10.1128/spectrum.02684-22 (PMC10100970; doi:10.1128/spectrum.02684-22)
Supplement: Supplemental file 1 — Fig. S1 to S7 and Tables S1 to S5. Download spectrum.02684-22-s0001.pdf, PDF file, 2.2 MB [file spectrum.02684-22-s0001.pdf]

Supplementary Information

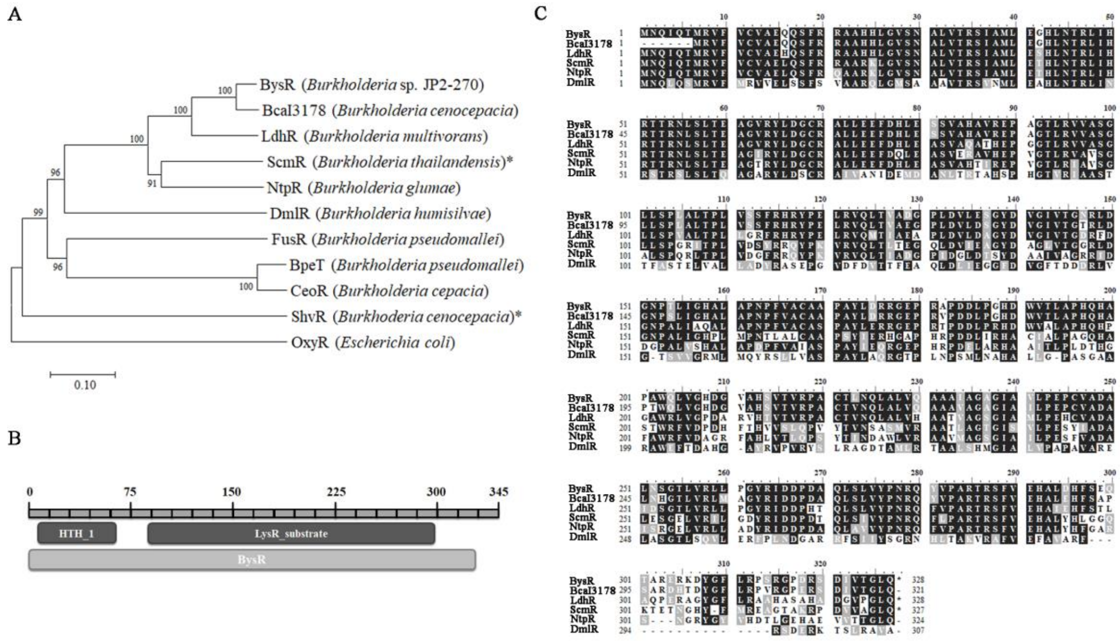

Supplementary Figure S1: Structure and correlation of BysR and related LTTR proteins. A. Phylogenetic tree based on amino acid sequences of BysR and related LTTR proteins. The tree was generated with MEGA7 using the neighbor-joining method and a bootstrap value of 1,000 iterations. The LTTRs marked with stars are involved in the regulation of secondary metabolites. B. Domain structure of BysR was analyzed by using the Pfam program at Pfam: Home page (xfam.org). C. Multiple sequence alignments for BysR. The alignments were conducted using MEGA7 (ClustalW algorithm). The consensus sequences are represented in black background. The similar sequences are shaded with grey background. The references protein BcaI3178 (I35_RS03450), LdhR (WP_012214022.1), ScmR (BTH_I1403), NtpR (WP_012734779.1), DmlR (WP_175225518.1), FusR (WP_004531197.1), BpeT (ALV61276.1),CeoR (WP_006484497.1), ShvR (ALV61276.1) and OxyR (AAN83350.1) were obtained from NCBI.

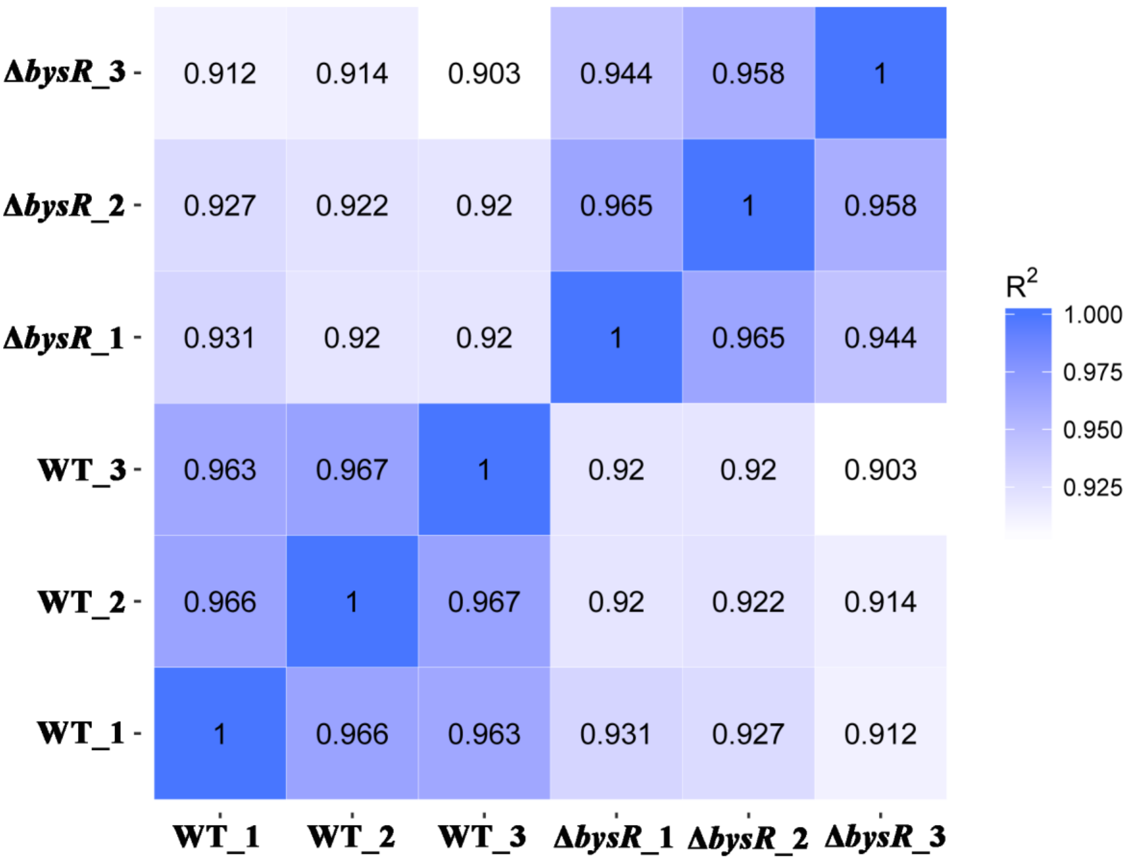

Supplementary Figure S2: The pearson correlation coefficient between replicates of RNA-seq. WT_1,WT_2,WT_3 and Δ*bysR*_1, Δ*bysR*_2, Δ*bysR*_3 represent the three replication of wild type isolate JP2-270 and *bysR* deletion isolate Δ*bysR*, respectively.

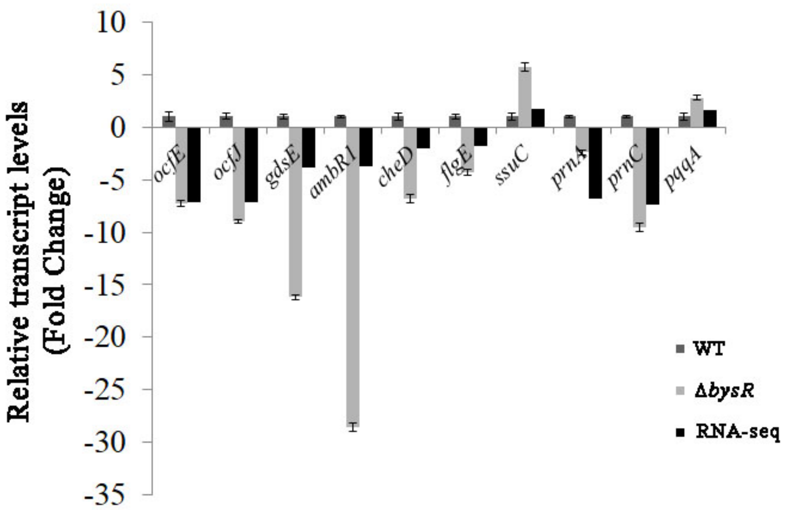

Supplementary Figure S3: The transcription levels of representative genes in WT, Δ*bysR* and RNA-Seq results. The *recA* gene is used as a control. The relative gene expression levels are presented as expression ratios of the indicated genes.

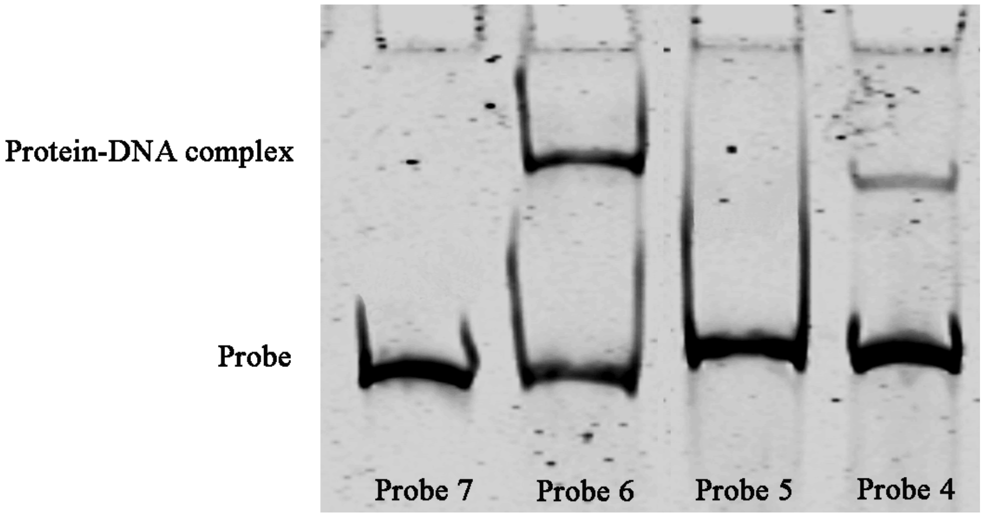

Supplementary Figure S4: EMSA verification of BysR binding consensus box. Probe 4: The wild type promoter DNA probe of gene DM992_38905. Probe 5: The mutated promoter DNA probe of gene DM992_38905. Probe 6: The wild type promoter DNA probe of gene DM992_31485. Probe 7: The mutated promoter DNA probe of gene DM992_31485.

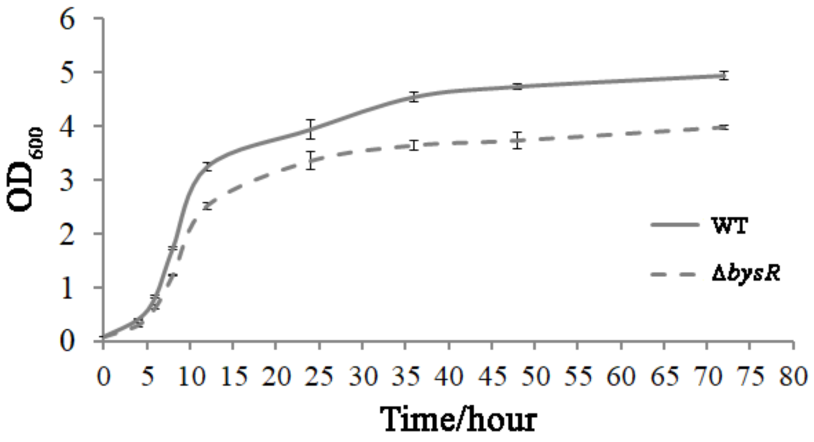

Supplementary Figure S5: Growth curve of JP2-270 and Δ*bysR* cultured in rich medium. Data are expressed as mean ± SD of triplicate.

>The enriched peak closest to *ambR1* (Merged-Chr3-148813-2|Chr3:188426-149308)

ATGCACTGAAGGCCGGAACTGGAAAACCCGGCACCGGCCCGGAGCGGGCACGTTCCCGGCGGATTCAACGAAAAAATCAC
GATGAAAAAATGAAACTATCCTTTCCATTGAATTTTATCCACGAATACCCCAGCCGCGTATGATAGCGGCGATTTAAAATATC
TGCATTTATCCTGTAATTAGTTTTTAGCCACGCCGCTTGCAGACCAAAAATCAACAAATCTGCAAGCCGTTCACGAACAAA
ATTCTGGCAGGCCAGATAAATCTGGCCTTTGTTTACCACGAAATTTTGTAATAGTCACCCGAGCACCGCATACCATGGATTTT
CGTCCAAGAGGCCTTATTCGAGAGAAGAATCGGCGATTTTCATCACGTCACCACCGCTCACCGACGTGGCCTCAAGCACT
TTCCTCCGGACATTGTTTCGATTTGATAAACGATCGATTCAATCTTCGTGATGGAAAAGCACAATATTCGCCGCCTACTTTC
GGCAGCCCATTGTCGATTACACCTTCAGGCGATAAAAATCGCCGCAAATAACATTTCCGGCGCCGCCGATACGGGTGTCG
CAAAATATCAACAAATGGCGAAATCATTGCCCGTCACAAAATATCCATATTGAATCACACGTAATTTATCCCCCCGCCGCTT
GCCGCCCGGCAGATAAATTGCTACGCTTTTCCAGCCAAATAAACGACAAATCGCCAGCCGATTTTGAACTGCAGTCAGAT
TTATTTAAAAAAACGAAACATGAAAATAAATATGCACGCGCAATCATCTTTTATCGATCGTCCCGGCTAGCGCTTCGTCAC
ACGCGATTCAGTCGAATTTCCATATCCACCTGCCGGCAGGGCAGGCCGACGGCCAGTCGAAGAAGTCGCC

>*ambR1* promoter region

CTGACTCTCCTTGTTTCCTTGTTGTGCGTGCTTGAGGACGGCGGCGCAACGCCAGCGCGGGCGAGCCGGCGCGGTTTGC
GCGGCGCGCGTTTCGTCGCGCGTTCTCGCATTGCACGCGCCGATGTGTTCGGCTCGCGCGAAACGGCGGCGCGCCGTGC
TCCTCTGTTCAGTTCGATGCGAAACCGCGTGCGGGGACGACGGCGGCGGCCAAGCCGCGGTCGCATTGAGTGGCGATCG
TTACCAATGCCCCTGCAAGAAGCTGAAACGTCCATTTCAGTCATTGAGCGTTGCTGGGTGAAAACGTGCTGCCGCCGAAT
GCACTGAAGGCCGGAACTGGAAAACCCGGCACCGGCCCGGAGCGGGCACGTTCCCGGCGGATTCAACGAAAAAATCAC
GATGAAAAAATGAAACTATCCTTTCCATTGAATTTTATCCACGAATACCCCAGCCGCGTATGATAGCGGCGATTTAAAATAT
CTGCATTTATCCTGTAATTAGTTTTTAGCCACGCCGCTTGCAGACCAAAAATCAACAAATCTGCAAGCCGTTCACGAACA
AAATTCTGGCAGGCCAGATAAATCTGGCCTTTGTTTACCACGAAATTTTGTAATAGTCACCCGAGCACCGCATACCATGGA
TTTTCGTCCAAGAGGCCTTATTCGAGAGAAGAATCGGCGATTTTCATCACGTCACCACCGCTCACCGACGTGGCCTCAAG
CACTTTCCTCCGGACATTGTTTCGATTTGATAAACGATCGATTCAATCTTCGTGATGGAAAAGCACAATATTCGCCGCCTAC
TTTCGGCAGCCCATTGTCGATTACACCTTCAGGCGATAAAAATCGCCGCAAATAACATTTCCGGCGCCGCCGATACGGGTG
TCGCAAAATATCAACAAATGGCGAAATCATTGCCCGTCACAAAATATCCATATTGAATCACACGTAATTTATCCCCCCGCCG
CTTGCCGCCCGGCAGATAAATTGCTACGCTTTTCCAGCCAAATAAACGACAAATCGCCAGCCGATTTTGAACTGCAGTCA
GATTTATTTAAAAAAACGAAACATGAAAATAAATATGCACGCGCAATCATCTTTTATCGATCGTCCCGGCTAGCGCTTCGTC
ACACGCGATTCAGTCGAATTTCCATATCCACCTGCCGGCAGGGCAGGCCGACGGCCAGTCGAAGAAGTCGCCGGCGATT
TTTCGATTCGCTTCCAGACGTCCGTGATGACGCGCGGCCGCGCATTGTCGAATGCACCGTCCTCTTTTCAATCCTGGCCTG
CCAGCGCGCAACGCTCCCCGCGCGTCACGCCTGCTCGAATCACCACACTGGCCTCAACGCGTCATTTGTCGTCTTTCCGA
TCTGTAAAACAATTCCGAGAAATTGGAGCACACCATGAGTCACGACTTTCGAGACGAGCCTGCGCCGCGTCGCGCCTTC
CTGGCCAAC

Supplementary Figure S6: The analysis of *ambR1* promoter region. The sequence marked with a double underline represents the sequence of probe 1 in EMSA assay. The sequence marked by a wavy line represents the sequence of probe 2 in EMSA assay. The sequence highlighted in red represents the sequences of probe 3 in EMSA. The sequences marked with grey background represent the putative motifs of BysR. The italicized and bold sequence in the enriched peak closest to *ambR1* is the partial sequence of probe 3.

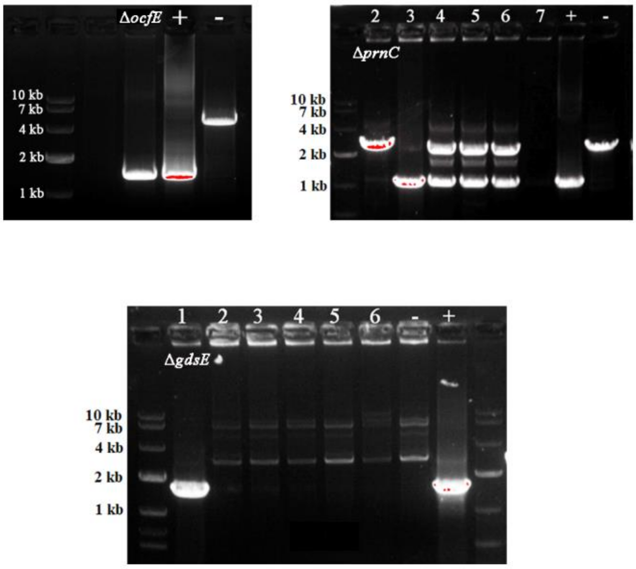

Supplementary Figure S7: PCR identification of target gene deletion mutants of JP2-270. +: The positive control, and the vectors with homologous arms were used for PCR. -: The negative control, and the wild type DNA was used as the template for PCR. Δ*ocfE* represents the in-frame deletion mutant of *ocfE*, Δ*gdsE* represents the in-frame deletion mutant of *gdsE* and Δ*prnC* represents the in-frame deletion mutant of *prnC*.

Supplementary Table S1 Primers used in this study

Primers	Sequence (5'-3')
ambR1-qRTF	TATCGGCGAAATTCTTCAGG
ambR1-qRTR	GGATTTCAGTGCCATCTCGT
prnA-qRTF	TCCGTTCAACCAGAAGTTCC
prnA-qRTR	GCCATCAAGGAAAAGGTTCA
prnC-qRTF	GATGTCCGTTTTCGGGTAGA
prnC-qRTR	CGACGACATCTTCAAGGTCA
gdsH-qRTF	GTCGAGGGGGTGTACAAGAA
gdsH-qRTR	GCGATATTGTCCTGGTACGG
33465-qRTF	ACCCGTGAATCCCCTTTTAC
33465-qRTR	GTCTACACGTGCAGCGTCAT
recA-qRTF	CGGCTCAATCAAGAAGAACG
recA-qRTR	AGATGCCCTCGCCATACAG
ocfE-qRTF	CGTCGGATGCCTATTTCAG
ocfE-qRTR	GCTTTCGGTTTCTTCCCAGT
ocfJ-qRTF	AGGAGCCGAATCAGACAGG
ocfJ-qRTR	AAAACGAACGCCGCCACT
pqqB-qRTF	GTGTTGTTGATGTGCGTGAG
pqqB-qRTR	ATGATCGACCTCGGCCTGT
CheD-qRTF	CATCTTCACTTCGTCGAGCA
CheD-qRTR	CAAGGCGAAGTACGAGAAGC
flgE-qRTF	ACGCTGACGACCTACTCGAT
flgE-qRTR	AGGACTCCGCATACTGGTTG
ssuC-qRTF	ATCAGCATCACCCACATCAA
ssuC-qRTR	GAGATGGCGAAGAGTTACGG
ocfEupF	*TCTAGA*GACGCATACCTGTTCAGCTG (XbaI)
ocfEupR	CCAGCGGATAGTGCGCATAGTCCTCCTGCCACTGTTTCACCAAAGCC
ocfEdwF	CAGGAGGACTATGCGCACTA
ocfEdwR	*AAGCTT*CGAAGAAGTGGAGATTGCCG (HindIII)
gdsEupF	*TCTAGA*GCCGAACATGCTGTCGTATT (XbaI)
gdsEupR	GGCCGGCGAGATGTGGAAGAACGCGGCATCTACCTCGTACCAGCAGC
gdsEdwF	GCCGCGTTCTTCCACATCT
gdsEdwR	*AAGCTT*TCCTCTCCATTTACTCGCCG (HindIII)
prnCupF	*AAGCTT*TCAGCGGCAAAGTTCATGAC (HindIII)
prnCupR	AATCAACAGTTTCGCGTGCTGATCATCAACTCGAAGTACAGCCTGCT
prnCdwF	GATGATCAGCACGCGAAACT

prnCdwR	*TCTAGA*CGCCGGAAATCCTGTAGTG (XbaI)
Test-ocfEF	CGGCCTGTTCATCAATACGG
Test-ocfER	GGAAGATGAGCAGGCTTTCG
Test-prnCF	GCCCACCTTCTTGATCGAGA
Test-prnCR	AGTTTCGCGTGCTGATCATC
Test-gdsEF	GCCGAACATGCTGTCGTATT
Test-gdsER	GTGCACGTCGAGGAAGAAC
pCS-F	tcccccgggctgcaggaattcCGTTGAATAGAACTCTTCACATCCTC
pCS-R	gataagcttgatatcgaattcGTTACTTGTTCCTAAAAAGATAAAACGG
CambR1-F	cgctctagaactagtggatccATGTTCGCGATGTTCTCGAAG
CambR1-R	ttcctgcagcccgggggatccTCACGCCGCCGACGCGCA
CambR2-F	cgctctagaactagtggatccATGGAGTTCAGCAGATTGTTCGC
CambR2-R	ttcctgcagcccgggggatccTCAGGTGCCGAGGTACGGC
BysR-F	*GAATTC*ATGAACCAGATTCAGACCAT (*Eco*R I)
BysR-R	*GTCGAC*TTACTGCAGGCCCGTGACGA (*Sal* I)
EMSA-ambR1-1F	Cy5’- CGATTTTCATCACGTCACCAC
EMSA-ambR1-1R	ATTTTTATCGCCTGAAGGTGTAATC
EMSA-ambR1-2F	Cy5’-CTCCTTGTTTCCTTGTTGTGC
EMSA-ambR1-2R	CAGTTCCGGCCTTCAGTG
EMSA-ambR1-3F	Cy5’- CCTCTGTTCAGTTCGATGCG
EMSA-ambR1-3R	GACGTGATGAAAATCGCCGA
EMSA-38905-F	Cy5’- GGCCCATCGATTTCAGACAG
EMSA-MT38905-R	GCTGAATAATCTCAGCGATT
EMSA-MT38905-F	AATGCTGTCAAAATCAAGATCGTCACGAATCGCTGAGATTATTCAGC
EMSA-38905-R	TCTCGCCATCAACGGAAGAT
EMSA-31485-F	Cy5’- GGTGACTCGTCCATCATTGC
EMSA-MT31485-R	GCGATTTTCACATAGTCCACT
EMSA-MT31485-F	TTGAAAATTTGTGAGATAAGGTGAGGAGTGGACTATGTGAAAATCGC
EMSA-31485-R	TTTTCAGATCAACCGTGCCG

Note：Red letters indicate sites of point mutations in the motif sequence.

Supplementary Table S2 The DEGs in Δ*bysR* relative to WT

Gene_id	Readcount _BysR	Readcount _WT	log2 Fold Change	pval	padj	gene _name	description
DM992_00925	3.1756642	17.557832	-2.467	0.000336	0.0065564	--	EF-hand domain-containing protein
DM992_00930	25.640139	98.839943	-1.9467	1.97E-09	1.09E-07	--	peptidase S10
DM992_01600	12.457677	40.928935	-1.7161	2.17E-05	0.000599	--	hypothetical protein
DM992_02890	17.927326	39.204006	-1.1288	0.002007	0.030115	--	nitronate monooxygenase
DM992_02925	8.6210366	26.628976	-1.6271	0.000823	0.014421	--	MinD/ParA family protein
DM992_02935	23.029892	55.54123	-1.2701	0.000118	0.0026941	flhA	flagellar biosynthesis protein FlhA
DM992_02970	19.056252	67.186052	-1.8179	6.12E-08	2.74E-06	cheZ	protein phosphatase CheZ
DM992_02975	32.838356	86.04828	-1.3898	1.55E-06	5.59E-05	cheY	chemotaxis protein CheY
DM992_02980	22.391181	78.601761	-1.8116	1.35E-08	6.66E-07	--	chemotaxis response regulator protein-glutamate methylesterase
DM992_02985	18.877453	75.307757	-1.9961	9.51E-10	5.43E-08	cheD	chemoreceptor glutamine deamidase CheD
DM992_02990	20.625555	124.97072	-2.5991	1.30E-18	1.43E-16	--	chemotaxis protein CheR
DM992_02995	51.059574	218.61924	-2.0982	1.90E-18	2.04E-16	--	methyl-accepting chemotaxis protein
DM992_03000	12.876611	62.934542	-2.2891	6.06E-10	3.53E-08	cheW	chemotaxis protein CheW
DM992_03005	54.619022	242.25911	-2.1491	1.59E-20	2.00E-18	cheA	chemotaxis protein CheA
DM992_03010	2.3748545	21.514339	-3.1794	6.42E-07	2.45E-05	--	response regulator
DM992_03015	16.622897	62.926161	-1.9205	1.06E-07	4.55E-06	MotB	motility protein MotB
DM992_03020	37.578477	178.54239	-2.2483	6.42E-19	7.28E-17	motA	flagellar motor stator protein MotA
DM992_03030	48.660547	100.98616	-1.0533	0.000146	0.0032002	flhD	flagellar transcriptional regulator FlhD
DM992_03075	337.43177	2966.1428	-3.1359	2.02E-25	3.17E-23	fliC	flagellin
DM992_03080	40.345618	108.16315	-1.4227	1.58E-07	6.69E-06	fliD	flagellar hook protein FliD
DM992_03085	4.9066029	23.716857	-2.2731	2.57E-05	0.0007023	--	flagellar protein FliT
DM992_03105	16.85025	44.068465	-1.387	0.000241	0.0048583	--	glycosyltransferase
DM992_03110	29.271332	71.203023	-1.2825	5.13E-05	0.0012955	--	hypothetical protein
DM992_03115	27.946442	63.793946	-1.1908	0.001155	0.019166	--	FAD-dependent oxidoreductase
DM992_03135	31.790762	83.828932	-1.3988	4.20E-06	0.0001382	--	glycosyltransferase family 1 protein
DM992_03685	59.808126	133.21619	-1.1554	5.76E-06	0.0001837	emrB	MFS transporter
DM992_03725	6.1553651	26.544366	-2.1085	3.53E-05	0.0009256	fliN	flagellar motor switch protein FliN

DM992_04000	1004.7751	493.9035	1.0246	0.000104	0.0024076	--	hypothetical protein
DM992_04105	348.56621	150.84986	1.2083	4.38E-09	2.30E-07	--	alpha/beta hydrolase
DM992_04415	47.907103	234.50951	-2.2913	7.48E-21	9.69E-19	--	hypothetical protein
DM992_04510	11.745313	40.198144	-1.775	1.69E-05	0.0004868	fliI	flagellar protein export ATPase FliI
DM992_04520	16.240558	47.078247	-1.5355	3.05E-05	0.0008243	fliG	flagellar motor switch protein FliG
DM992_04535	6.0574352	21.548072	-1.8308	0.001199	0.01969	fliS	flagellar export chaperone FliS
DM992_04720	13.25091	40.471204	-1.6108	5.50E-05	0.0013739	flgM	flagellar biosynthesis anti-sigma factor FlgM
DM992_04725	24.043261	51.875437	-1.1094	0.000473	0.0088981	flgA	flagellar biosynthesis protein FlgA
DM992_04745	41.695145	145.23866	-1.8005	0.0001	0.0023429	flgE	flagellar hook protein FlgE
DM992_04750	27.767642	71.829465	-1.3712	0.000436	0.0082688	flgF	flagellar basal-body rod protein FlgF
DM992_04760	6.0279532	26.263991	-2.1233	3.74E-05	0.0009788	flgH	flagellar basal body L-ring protein FlgH
DM992_04765	14.678472	35.65302	-1.2803	0.001944	0.029321	flgI	flagellar biosynthesis protein FlgI
DM992_04770	13.892815	43.600904	-1.65	3.82E-05	0.0009959	flgJ	flagellar assembly peptidoglycan hydrolase FlgJ
DM992_04785	34.300501	155.58955	-2.1814	8.91E-17	9.09E-15	flgK	flagellar hook-associated protein FlgK
DM992_04790	30.451543	183.44059	-2.5907	1.12E-22	1.55E-20	flgL	flagellar hook-associated protein 3
DM992_04870	5.4354251	18.868789	-1.7955	0.001961	0.029533	potH	putrescine ABC transporter permease PotH
DM992_04875	8.0579904	28.445829	-1.8197	0.000252	0.0050512	--	polyamine ABC transporter ATP-binding protein
DM992_04880	53.486177	114.47035	-1.0977	1.43E-05	0.0004173	--	polyamine ABC transporter substrate-binding protein
DM992_04885	10.733851	29.186137	-1.4431	0.000898	0.015496	--	aspartate aminotransferase family protein
DM992_04895	46.708801	96.392429	-1.0452	6.71E-05	0.0016433	--	gamma-glutamyl-gamma-aminobutyrate hydrolase family protein
DM992_05130	216.76638	104.18521	1.057	1.29E-06	4.75E-05	--	Na+/H+ antiporter
DM992_05700	16.044699	44.04287	-1.4568	0.000181	0.0037907	--	hypothetical protein
DM992_05785	78.799642	173.91084	-1.1421	0.000148	0.0032376	cydX	cytochrome bd-I oxidase subunit CydX
DM992_06070	160.79397	381.7033	-1.2472	5.22E-10	3.06E-08	--	ParA family protein
DM992_06080	207.50081	1943.1518	-3.2272	1.83E-60	7.48E-58	--	phasin
DM992_06085	10.408568	83.311503	-3.0007	1.73E-16	1.68E-14	--	acetyl-CoA C-acetyltransferase
DM992_06090	8.6087183	100.44849	-3.5445	1.82E-22	2.48E-20	--	beta-ketoacyl-ACP reductase
DM992_06095	3.0287176	17.40658	-2.5229	0.000218	0.0044561	--	hypothetical protein
DM992_06920	13.995127	32.799916	-1.2288	0.002873	0.040847	--	TetR family transcriptional regulator
DM992_08195	41.846113	17.600768	1.2495	0.00106	0.017879	--	oxidoreductase
DM992_08270	396.33034	949.78004	-1.2609	2.48E-12	1.84E-10	--	chromate transporter
DM992_10590	57.560684	133.82126	-1.2172	1.86E-06	6.64E-05	--	transposase

DM992_11020	33.03004	73.914463	-1.1621	0.000394	0.007591	--	hypothetical protein
DM992_11110	310.06033	150.9728	1.0383	4.39E-07	1.74E-05	--	short-chain dehydrogenase/reductase
DM992_11205	166.25614	75.972659	1.1299	1.98E-06	7.01E-05	--	hypothetical protein
DM992_11935	79.861512	162.42345	-1.0242	2.47E-05	0.0006764	--	nitronate monooxygenase
DM992_11940	419.64567	1034.1753	-1.3012	1.16E-13	9.24E-12	--	OmpW family protein
DM992_12040	67.822306	140.6692	-1.0525	2.15E-05	0.0005977	--	AcrB/AcrD/AcrF family protein
DM992_12045	11.267879	31.271762	-1.4726	0.000988	0.016896	--	efflux RND transporter periplasmic adaptor subunit
DM992_12065	632.31996	1443.7502	-1.1911	1.12E-11	7.92E-10	--	YeiH family putative sulfate export transporter
DM992_12125	20229.37	7051.7228	1.5204	0.000131	0.0029264	--	saccharopine dehydrogenase
DM992_12225	35.218879	89.950758	-1.3528	5.55E-06	0.0001785	--	MerR family transcriptional regulator
DM992_12230	129.83943	290.08878	-1.1598	7.48E-08	3.34E-06	--	J domain-containing protein
DM992_12260	431.11368	913.4304	-1.0832	2.69E-09	1.46E-07	--	hypothetical protein
DM992_12265	165.74356	532.25982	-1.6832	3.95E-08	1.84E-06	--	carbamate kinase
DM992_12270	210.08173	486.90188	-1.2127	7.18E-10	4.13E-08	--	ornithine carbamoyltransferase
DM992_12275	251.0875	651.23953	-1.375	5.50E-13	4.27E-11	--	arginine deiminase
DM992_12280	343.99947	943.16893	-1.4551	2.38E-11	1.61E-09	--	arginine:agmatine antiporter
DM992_12300	77.292242	156.18517	-1.0149	1.75E-05	0.0005012	--	hypothetical protein
DM992_12310	78.623781	158.36109	-1.0102	0.000427	0.0081153	--	hemolysin D
DM992_12325	105.29347	219.34643	-1.0588	3.57E-06	0.00012	--	hypothetical protein
DM992_12340	38.151934	78.728171	-1.0451	0.000341	0.0066284	--	hypothetical protein
DM992_12370	1243.7373	2653.8409	-1.0934	2.24E-10	1.37E-08	--	universal stress protein
DM992_12375	466.85773	1038.9775	-1.1541	1.49E-09	8.33E-08	--	universal stress protein
DM992_12380	1218.0802	2666.7398	-1.1305	5.14E-08	2.33E-06	--	universal stress protein
DM992_12400	596.31997	1699.5162	-1.511	1.21E-10	7.67E-09	--	alcohol dehydrogenase
DM992_12405	233.75816	501.67717	-1.1017	1.34E-08	6.63E-07	--	nitroreductase
DM992_12430	473.31059	1139.6271	-1.2677	8.66E-13	6.60E-11	--	hypothetical protein
DM992_12435	324.54738	724.35574	-1.1583	4.52E-10	2.68E-08	--	OsmY domain-containing protein
DM992_12445	734.34541	1559.0555	-1.0861	6.91E-10	4.00E-08	--	Hsp20/alpha crystallin family protein
DM992_12455	312.63156	832.99768	-1.4138	2.80E-14	2.33E-12	--	universal stress protein
DM992_12645	256.71677	614.75218	-1.2598	2.56E-11	1.70E-09	--	amino acid ABC transporter substrate-binding protein
DM992_13260	135.02415	62.219133	1.1178	0.000132	0.0029445	--	phenylacetic acid degradation protein
DM992_13355	21.056448	6.2792564	1.7456	0.001269	0.020573	ssuC	aliphatic sulfonate ABC transporter permease ssuC

DM992_13855	116.23375	270.96866	-1.2211	2.69E-06	9.29E-05	--	hypothetical protein
DM992_13865	93.392143	194.53595	-1.0587	2.40E-06	8.36E-05	--	heavy metal translocating P-type ATPase
DM992_13875	1344.8723	2721.594	-1.017	3.11E-09	1.67E-07	--	hypothetical protein
DM992_13885	157.27632	403.05025	-1.3577	2.02E-11	1.37E-09	--	carboxymuconolactone decarboxylase family protein
DM992_13890	535.63356	1116.4536	-1.0596	1.28E-09	7.24E-08	--	hypothetical protein
DM992_13925	37.57657	104.17141	-1.4711	1.72E-07	7.11E-06	--	CreA family protein
DM992_13930	125.2111	372.37485	-1.5724	1.38E-14	1.18E-12	--	universal stress protein
DM992_13945	90.354765	228.46461	-1.3383	1.31E-09	7.40E-08	--	ABC transporter ATP-binding protein
DM992_13950	63.673785	192.08679	-1.593	1.65E-11	1.13E-09	--	transcriptional regulator
DM992_13955	241.25502	570.21421	-1.2409	6.64E-11	4.23E-09	--	efflux RND transporter periplasmic adaptor subunit
DM992_13960	232.07001	467.05076	-1.009	2.12E-06	7.46E-05	--	AcrB/AcrD/AcrF family protein
DM992_13970	108.93394	218.92112	-1.007	3.60E-06	0.0001205	--	porin
DM992_16145	69.540822	209.99341	-1.5944	0.000172	0.0036361	--	DUF4088 domain-containing protein
DM992_17160	11.414825	35.049845	-1.6185	0.000262	0.0052327	--	glycosyl transferase
DM992_17165	27.681671	63.947157	-1.2079	0.000106	0.0024369	--	glycosyltransferase family 1 protein
DM992_17170	123.80858	263.68449	-1.0907	6.10E-07	2.35E-05	galE	UDP-glucose 4-epimerase GalE
DM992_17470	239.55887	1520.4055	-2.666	3.49E-44	8.37E-42	bysR	LysR family transcriptional regulator
DM992_17730	0.2962641	11.63133	-5.295	2.15E-05	0.0005977	--	hypothetical protein
DM992_17735	6.7773752	69.664215	-3.3616	1.33E-16	1.33E-14	--	serine protease
DM992_17775	124.42188	301.70775	-1.2779	4.26E-07	1.70E-05	--	OsmY domain-containing protein
DM992_17780	37.292264	89.968099	-1.2705	1.20E-05	0.0003565	--	glutamyl-tRNA amidotransferase
DM992_17785	209.8235	533.70341	-1.3469	2.71E-12	1.98E-10	ftsH	cell division protein FtsH
DM992_17790	84.31403	194.21271	-1.2038	1.51E-06	5.50E-05	--	adenosylcobalamin-dependent ribonucleoside-diphosphate reductase
DM992_17795	62.939876	157.82776	-1.3263	4.45E-08	2.06E-06	--	1-phosphofructokinase family hexose kinase
DM992_17800	75.069899	188.9651	-1.3318	1.47E-08	7.12E-07	--	polysaccharide deacetylase family protein
DM992_17810	160.64192	406.19093	-1.3383	2.31E-07	9.49E-06	--	serine hydrolase
DM992_18300	426.18321	1085.0617	-1.3482	3.15E-13	2.47E-11	--	shikimate transporter
DM992_18385	40.281089	91.175969	-1.1786	1.50E-05	0.000436	--	chemotaxis protein
DM992_18390	781.3433	2205.3549	-1.497	3.83E-17	3.96E-15	--	hypothetical protein
DM992_18400	672.61044	1458.5125	-1.1167	5.48E-06	0.0001774	--	universal stress protein
DM992_18445	13.165298	31.586621	-1.2626	0.0035	0.04858	pilZ	pilus assembly protein PilZ
DM992_18625	43.214248	11.478502	1.9126	0.001644	0.025637	--	S58 family peptidase

DM992_18855	3406.2206	1503.2165	1.1801	3.25E-05	0.0008704	--	glycoside hydrolase family 68 protein
DM992_18860	412.79633	202.67405	1.0263	0.00086	0.014927	--	glycoside hydrolase family 32 protein
DM992_19210	49.049999	19.856875	1.3046	0.000125	0.002828	--	acyl-CoA dehydrogenase
DM992_19265	32.363756	13.551201	1.256	0.002097	0.03121	--	oligosaccharide repeat unit polymerase
DM992_19690	38.112968	14.555365	1.3887	0.000324	0.0063357	--	hypothetical protein
DM992_19710	17.508855	121.81202	-2.7985	7.49E-20	9.12E-18	--	acyltransferase
DM992_19980	75.302097	319.50957	-2.0851	1.14E-20	1.45E-18	--	amino acid permease
DM992_19985	243.15084	1250.6606	-2.3628	7.73E-35	1.50E-32	aspA	aspartate ammonia-lyase
DM992_19995	864.78257	2232.9478	-1.3685	1.35E-14	1.17E-12	--	asparaginase
DM992_20000	40.123111	12.298819	1.7059	2.00E-05	0.0005665	--	TonB-dependent siderophore receptor
DM992_20485	162.43976	352.91196	-1.1194	2.89E-08	1.37E-06	--	alpha/beta hydrolase
DM992_20495	10.604068	27.65571	-1.383	0.00206	0.030791	--	ribosome-associated translation inhibitor RaiA
DM992_20515	222.83118	463.37782	-1.0562	4.82E-08	2.21E-06	--	CBS domain-containing protein
DM992_20535	42.409056	115.32174	-1.4432	3.54E-08	1.66E-06	--	Hsp20/alpha crystallin family protein
DM992_20550	92.716941	260.23799	-1.4889	2.84E-08	1.36E-06	--	hypothetical protein
DM992_20555	58.669612	204.50969	-1.8015	4.68E-14	3.86E-12	--	nicotinate phosphoribosyltransferase
DM992_20595	247.49615	511.61969	-1.0477	1.78E-06	6.39E-05	--	phosphoenolpyruvate synthase
DM992_20970	52.129641	24.037768	1.1168	0.001186	0.019588	--	metallophosphoesterase
DM992_21725	33.868889	186.38517	-2.4603	9.79E-22	1.29E-19	asnB	asparagine synthase (glutamine-hydrolyzing)
DM992_21875	114.38571	242.57171	-1.0845	2.08E-05	0.0005829	atpC	ATP synthase F1 subunit epsilon
DM992_22265	84.410052	185.08936	-1.1327	6.96E-07	2.64E-05	--	DUF2158 domain-containing protein
DM992_22685	32.745168	234.14757	-2.8381	2.70E-24	4.01E-22	--	short-chain dehydrogenase/reductase
DM992_23330	314.20035	125.32965	1.326	2.00E-10	1.24E-08	pqqE	pyrroloquinoline quinone biosynthesis protein PqqE
DM992_23340	116.63088	51.516191	1.1789	0.000459	0.0086738	pqqC	pyrroloquinoline quinone biosynthesis protein PqqC
DM992_23345	404.78221	157.09684	1.3655	1.22E-11	8.56E-10	--	pyrroloquinoline quinone biosynthesis protein PqqB
DM992_23350	32.028422	10.308144	1.6356	0.000243	0.0048915	pqqA	pyrroloquinoline quinone precursor peptide PqqA
DM992_23365	156.43598	70.223388	1.1555	3.68E-06	0.0001225	--	prolyl aminopeptidase
DM992_23370	129.53105	62.739727	1.0458	1.67E-05	0.0004834	--	TonB-dependent receptor
DM992_23375	4707.5445	1890.7698	1.316	8.85E-05	0.0021118	--	PQQ-dependent dehydrogenase%2C methanol/ethanol family
DM992_23380	992.48657	369.92105	1.4238	0.001713	0.026419	--	cytochrome C
DM992_23385	120.16579	46.481249	1.3703	1.05E-07	4.54E-06	pqqB	High potential iron-sulfur protein
DM992_23390	560.34568	232.25158	1.2706	2.48E-11	1.66E-09	--	RNA polymerase subunit sigma-70

DM992_23570	57.672839	27.373886	1.0751	0.002103	0.03121	--	DUF4148 domain-containing protein
DM992_23850	54.766432	128.68323	-1.2325	7.37E-07	2.76E-05	--	SMP-30/gluconolaconase/LRE-like region family protein
DM992_24035	7.722297	266.12348	-5.1069	0.000582	0.010555	--	LysE family translocator
DM992_24060	54.756948	124.1992	-1.1815	6.68E-06	0.0002096	--	5-methyltetrahydropteroyltriglutamate--homocysteine S-methyltransferase
DM992_24185	4117.3627	1922.9051	1.0984	9.58E-09	4.80E-07	--	MFS transporter
DM992_24190	1431.9923	617.74675	1.2129	1.23E-11	8.56E-10	--	shikimate dehydrogenase
DM992_24195	1536.2473	754.81131	1.0252	9.90E-09	4.93E-07	aroQ	type II 3-dehydroquinate dehydratase
DM992_24310	24.895922	8.639517	1.5269	0.001453	0.023092	--	methionine ABC transporter ATP-binding protein
DM992_24475	27.062855	73.532961	-1.4421	2.67E-06	9.28E-05	--	hypothetical protein
DM992_24480	55.373753	184.8934	-1.7394	1.40E-12	1.05E-10	--	hypothetical protein
DM992_24780	60.719751	11.651188	2.3817	0.000716	0.01267	--	NAD-dependent dehydratase
DM992_24785	217.70996	101.96189	1.0944	3.91E-05	0.0010158	--	MarR family transcriptional regulator
DM992_24995	1089.0486	517.17402	1.0743	4.87E-09	2.53E-07	--	acyl-CoA thioesterase
DM992_25000	1166.9602	544.48316	1.0998	0.000501	0.0092911	--	enoyl-CoA hydratase
DM992_25005	2626.9653	1114.4053	1.2371	0.000227	0.0046273	--	acyl-CoA dehydrogenase
DM992_25010	748.95445	291.94056	1.3592	3.44E-10	2.06E-08	--	hypothetical protein
DM992_25015	1427.1813	575.87232	1.3093	9.75E-08	4.26E-06	--	hypothetical protein
DM992_25155	524.56582	192.64676	1.4452	3.73E-11	2.43E-09	--	VOC family protein
DM992_26140	15.922029	107.455	-2.7546	3.37E-18	3.57E-16	--	DNA-binding protein
DM992_26145	22.317528	119.28166	-2.4181	3.28E-16	3.15E-14	--	TOMM system kinase/cyclase fusion protein
DM992_26150	13.843798	98.328691	-2.8284	8.25E-18	8.63E-16	--	hypothetical protein
DM992_26155	14.977107	90.663455	-2.5978	4.98E-15	4.42E-13	--	hypothetical protein
DM992_26160	19.636462	55.159101	-1.4901	2.72E-05	0.000741	--	bacteriocin biosynthesis cyclodehydratase
DM992_26490	84.188368	38.927794	1.1128	7.69E-05	0.0018509	--	hydrogenase 1 large subunit
DM992_26630	144.97883	304.6703	-1.0714	5.49E-07	2.14E-05	--	hypothetical protein
DM992_26640	815.46372	1670.2506	-1.0344	3.85E-09	2.04E-07	--	hypothetical protein
DM992_26645	215.54287	559.53433	-1.3763	1.24E-12	9.35E-11	--	CreA family protein
DM992_26650	120.51561	282.2878	-1.2279	6.21E-05	0.0015308	--	carboxypeptidase regulatory-like domain-containing protein
DM992_26680	167.40197	346.13735	-1.048	5.54E-07	2.15E-05	--	hypothetical protein
DM992_26690	397.71991	821.30319	-1.0462	5.27E-09	2.71E-07	--	hypothetical protein
DM992_26845	25.948721	83.050672	-1.6783	4.92E-08	2.24E-06	--	SMP-30/gluconolaconase/LRE-like region family protein
DM992_27155	53.78739	133.86293	-1.3154	4.08E-07	1.63E-05	--	AraC family transcriptional regulator

DM992_27160	8.7066481	752.71465	-6.4338	1.16E-54	4.51E-52	--	fucose-binding lectin II
DM992_27165	3.1438112	703.08827	-7.805	2.66E-34	5.04E-32	--	fucose-binding lectin II
DM992_27170	16.306739	10026.215	-9.2641	9.07E-111	5.29E-108	--	hypothetical protein
DM992_27875	36.359661	76.582778	-1.0747	0.00048	0.0089778	--	hypothetical protein
DM992_27885	175.81267	489.60373	-1.4776	2.20E-14	1.87E-12	--	LemA family protein
DM992_27895	23.277603	48.69442	-1.0648	0.002601	0.037364	--	hypothetical protein
DM992_28505	148.73734	68.628648	1.1159	6.77E-06	0.0002118	--	isocitrate lyase/phosphoenolpyruvate mutase family protein
DM992_28515	238.12992	118.06854	1.0121	7.36E-05	0.0017877	--	TetR family transcriptional regulator
DM992_28560	8.4693481	25.683825	-1.6005	0.002168	0.031874	--	DSD1 family PLP-dependent enzyme
DM992_29025	51.245487	3220.6643	-5.9738	9.57E-155	9.77E-152	--	fucose-binding lectin protein
DM992_29370	19.859433	81.444212	-2.036	3.45E-10	2.06E-08	--	Crp/Fnr family transcriptional regulator
DM992_29375	4.4562794	24.992505	-2.4876	1.89E-05	0.0005383	--	flagellar motor protein
DM992_29870	1169.804	572.54667	1.0308	9.74E-05	0.0022838	--	rubrerythrin
DM992_29890	579.91251	1652.51	-1.5108	9.58E-17	9.65E-15	--	transposase
DM992_29895	53.111981	990.30505	-4.2208	5.15E-25	7.94E-23	--	mannose-1-phosphate guanylyltransferase/mannose-6-phosphate isomerase
DM992_29900	18.29013	470.76294	-4.6859	1.03E-71	4.93E-69	--	undecaprenyl-phosphate glucose phosphotransferase
DM992_29905	17.386649	354.99097	-4.3517	2.41E-23	3.39E-21	--	UDP-glucose/GDP-mannose dehydrogenase family protein
DM992_29910	2.3505779	49.83836	-4.4062	1.43E-16	1.40E-14	--	low molecular weight phosphotyrosine protein phosphatase
DM992_29915	17.874031	322.12889	-4.1717	1.08E-48	3.16E-46	--	exopolysaccharide biosynthesis protein
DM992_29920	28.911362	496.25403	-4.1014	1.07E-62	4.60E-60	--	exopolysaccharide biosynthesis protein
DM992_29925	11.645012	207.22509	-4.1534	1.31E-41	2.82E-39	--	glycosyltransferase family 2 protein
DM992_29930	11.681607	211.32075	-4.1771	3.67E-43	8.32E-41	--	glycosyltransferase family 1 protein
DM992_29935	14.054091	241.31069	-4.1018	1.71E-45	4.65E-43	--	glucose-6-phosphate isomerase
DM992_29940	8.5868126	241.55575	-4.8141	8.33E-53	2.95E-50	--	glycosyltransferase family 1 protein
DM992_29945	13.103963	281.9856	-4.4275	4.39E-54	1.63E-51	--	glycosyltransferase family 1 protein
DM992_30720	72.426976	216.24195	-1.578	3.14E-12	2.27E-10	--	epimerase
DM992_30725	30.581789	327.72641	-3.4217	1.67E-33	3.11E-31	gmd	GDP-mannose 4%2C6-dehydratase
DM992_30730	15.819717	192.56979	-3.6056	7.82E-27	1.30E-24	--	acyltransferase
DM992_30735	25.633386	338.55917	-3.7233	3.32E-29	5.65E-27	--	hypothetical protein
DM992_30740	14.820213	134.2578	-3.1794	7.57E-25	1.14E-22	--	flippase
DM992_30745	18.478517	275.753	-3.8995	3.00E-45	7.64E-43	--	glycosyl transferase family 1
DM992_30750	20.770954	68.145265	-1.714	0.000141	0.0031004	--	acyltransferase

DM992_30755	187.75436	1361.1537	-2.8579	6.20E-30	1.08E-27	galU	UTP--glucose-1-phosphate uridylyltransferase
DM992_30760	9.5735347	39.223424	-2.0346	2.10E-05	0.0005871	--	acyltransferase
DM992_31245	505.09362	1503.1545	-1.5734	1.54E-18	1.68E-16	--	DUF3079 domain-containing protein
DM992_31250	3465.7134	8451.6504	-1.2861	2.80E-14	2.33E-12	--	hydroxylamine reductase
DM992_31255	744.24578	1773.9997	-1.2532	1.30E-05	0.0003835	--	oxidoreductase
DM992_31260	87.076892	207.30763	-1.2514	5.60E-08	2.52E-06	--	DUF296 domain-containing protein
DM992_31280	807.59305	400.04588	1.0135	0.001839	0.028104	--	universal stress protein
DM992_31420	33.225437	223.34448	-2.7489	3.18E-15	2.88E-13	--	EmrB/QacA family drug resistance transporter
DM992_31425	12.330265	47.111979	-1.9339	7.15E-07	2.70E-05	--	hypothetical protein
DM992_31485	62.244212	144.54583	-1.2155	5.58E-07	2.16E-05	clpV	type VI secretion system ATPase TssH
DM992_31625	65.515539	26.021952	1.3321	2.87E-05	0.0007785	--	LysR family transcriptional regulator
DM992_32860	31.415999	8.9435972	1.8126	9.58E-05	0.0022588	--	TonB-dependent receptor
DM992_33030	16.477962	48.112678	-1.5459	0.00129	0.020797	--	alpha/beta hydrolase
DM992_33035	24.599658	67.142363	-1.4486	1.41E-05	0.0004138	--	chlorinating enzyme
DM992_33040	38.122556	107.06631	-1.4898	1.61E-07	6.79E-06	--	hypothetical protein
DM992_33045	18.451406	72.627024	-1.9768	4.44E-09	2.32E-07	fabD	[acyl-carrier-protein] S-malonyltransferase
DM992_33060	16.950551	63.217629	-1.899	2.72E-08	1.31E-06	--	3-hydroxy-3-methylglutaryl-ACP synthase
DM992_33065	15.99094	43.587851	-1.4467	0.000187	0.0039016	--	hypothetical protein
DM992_33070	47.029805	109.16783	-1.2149	5.70E-06	0.0001823	--	hypothetical protein
DM992_33075	44.811485	210.3442	-2.2308	2.29E-19	2.63E-17	--	hypothetical protein
DM992_33080	16.674285	121.35738	-2.8636	1.79E-19	2.12E-17	--	DUF1697 domain-containing protein
DM992_33085	23.603349	76.4761	-1.696	7.35E-07	2.76E-05	--	hypothetical protein
DM992_33090	18.130865	95.753249	-2.4009	8.20E-14	6.62E-12	--	DUF3224 domain-containing protein
DM992_33095	42.369627	131.56403	-1.6347	0.000533	0.0098033	--	long-chain fatty acid--CoA ligase
DM992_33105	18.867969	53.910184	-1.5146	4.24E-05	0.0010882	--	hypothetical protein
DM992_33110	7.364698	21.703926	-1.5593	0.002004	0.030112	oprM	OprM
DM992_33200	2.6173599	65.989219	-4.656	6.19E-16	5.81E-14	--	hypothetical protein
DM992_33205	31.196223	169.83391	-2.4447	1.48E-19	1.78E-17	--	hypothetical protein
DM992_33210	17.978713	76.161366	-2.0828	1.83E-10	1.14E-08	--	cupin-like domain-containing protein
DM992_33315	77.145295	158.35906	-1.0375	2.28E-05	0.0006296	--	IS3 family transposase
DM992_33320	39.537696	132.37544	-1.7433	5.03E-11	3.26E-09	--	helicase
DM992_33325	12.692606	1793.2463	-7.1424	3.35E-162	5.47E-159	ocfN	thioesterase

DM992_33330	30.85249	1505.9402	-5.6091	5.51E-126	4.50E-123	ocfM	SDR family NAD-dependent epimerase/dehydratase
DM992_33335	53.158523	3101.2305	-5.8664	9.66E-47	2.72E-44	ocfL	diaminobutyrate--2-oxoglutarate transaminase
DM992_33340	11.772424	1343.8571	-6.8348	1.85E-25	2.97E-23	ocfK	TauD/TfdA family dioxygenase
DM992_33345	46.070194	6589.9544	-7.1603	3.06E-203	8.32E-200	ocfJ	beta-ketoacyl synthase
DM992_33350	43.873676	3206.4096	-6.1915	1.24E-159	1.69E-156	ocfI	short-chain dehydrogenase
DM992_33355	21.98704	5470.3835	-7.9588	1.83E-218	1.49E-214	ocfH	hypothetical protein
DM992_33360	23.66422	3413.6215	-7.1725	5.31E-173	1.08E-169	ocfH	hypothetical protein
DM992_33365	8.4912537	2066.5834	-7.9271	5.22E-113	3.28E-110	ocfH	hypothetical protein
DM992_33370	7.4654623	1616.2532	-7.7582	3.62E-125	2.69E-122	ocfG	MBL fold metallo-hydrolase
DM992_33375	19.720063	2583.0838	-7.0333	6.53E-125	4.44E-122	ocfF	non-ribosomal peptide synthetase
DM992_33380	46.282858	6669.2617	-7.1709	9.93E-204	4.05E-200	ocfE	non-ribosomal peptide synthetase
DM992_33385	83.074131	4957.8522	-5.8992	2.13E-35	4.25E-33	ocfD	non-ribosomal peptide synthetase
DM992_33390	2.0543138	488.042	-7.8922	3.31E-24	4.83E-22	ocfB	hypothetical protein
DM992_33395	47.044958	2485.8832	-5.7236	8.32E-31	1.51E-28	ocfA	ATP-binding protein
DM992_33405	39.674695	2007.8898	-5.6613	4.11E-133	3.72E-130	ambR2	LuxR family transcriptional regulator
DM992_33410	33.643907	430.58408	-3.6779	2.95E-45	7.64E-43	ambR1	LuxR family transcriptional regulator
DM992_33415	8.0018608	394.29037	-5.6228	5.61E-49	1.76E-46	--	FAD-linked oxidase
DM992_33430	7.03421	24.924413	-1.8251	0.000411	0.0078414	--	RND transporter
DM992_33825	44.569339	108.75341	-1.2869	4.49E-06	0.000147	--	glycosyltransferase
DM992_33830	3.4448172	13.398373	-1.9596	0.003625	0.049882	--	GtrA family protein
DM992_33835	7.1349743	451.726	-5.9844	1.43E-86	7.78E-84	--	inclusion body protein
DM992_33840	3.4523935	185.80937	-5.7501	3.42E-52	1.16E-49	--	inclusion body protein
DM992_33845	2.1375544	28.481451	-3.736	7.74E-09	3.90E-07	--	hypothetical protein
DM992_34385	4.6693028	21.199606	-2.1828	0.0003	0.0059333	xylB	xylulokinase
DM992_34390	6.5909994	28.331648	-2.1038	9.08E-05	0.0021534	xylA	xylose isomerase
DM992_34760	6.5985757	41.930387	-2.6678	5.62E-09	2.87E-07	--	hypothetical protein
DM992_35035	66.825069	10.98181	2.6053	6.27E-11	4.03E-09	--	LysE family translocator
DM992_35140	589.09082	3008.8248	-2.3526	3.69E-38	7.53E-36	--	hydrolase
DM992_35720	33.434078	73.313177	-1.1328	0.000194	0.0040182	--	nitroreductase
DM992_35750	28.967492	65.706258	-1.1816	0.000247	0.0049591	--	DUF2964 family protein
DM992_35760	632.41542	1466.4332	-1.2134	3.52E-12	2.52E-10	--	OsmY domain-containing protein
DM992_35765	109.26124	308.77793	-1.4988	8.10E-13	6.24E-11	--	glutamyl-tRNA amidotransferase

DM992_35770	437.90508	955.88884	-1.1262	2.43E-10	1.47E-08	--	ATP-dependent zinc metalloprotease FtsH
DM992_35775	64.131222	136.26853	-1.0874	4.00E-06	0.000132	--	adenosylcobalamin-dependent ribonucleoside-diphosphate reductase
DM992_35780	57.020987	136.94989	-1.2641	9.12E-06	0.0002757	--	1-phosphofructokinase family hexose kinase
DM992_35820	412.97916	1253.8076	-1.6022	1.01E-18	1.13E-16	raiA	ribosome-associated translation inhibitor RaiA
DM992_35825	813.11696	2111.2001	-1.3765	5.56E-15	4.88E-13	--	hypothetical protein
DM992_35830	99.207635	215.49377	-1.1191	1.08E-05	0.0003252	--	hypothetical protein
DM992_35835	53.496227	128.52972	-1.2646	1.15E-05	0.0003449	--	hypothetical protein
DM992_35840	97.981863	205.04976	-1.0654	1.53E-06	5.53E-05	groL	chaperonin GroEL
DM992_36625	54.921058	128.04179	-1.2212	8.38E-05	0.0020048	--	citrate-proton symporter
DM992_37480	1.3343738	9.9017996	-2.8915	0.002618	0.03748	--	IS5/IS1182 family transposase
DM992_38375	18.94353	68.737473	-1.8594	1.42E-08	6.95E-07	--	CHAD domain-containing protein
DM992_38760	108.05711	500.65754	-2.212	8.65E-27	1.41E-24	--	LysR family transcriptional regulator
DM992_38820	4.9360849	196.88341	-5.3178	1.49E-51	4.85E-49	prnD	2Fe-2S ferredoxin
DM992_38825	2.2331133	366.45744	-7.3584	1.63E-43	3.79E-41	prnC	FAD-dependent oxidoreductase
DM992_38830	3.8979751	207.05922	-5.7312	2.22E-39	4.65E-37	prnB	monodechloroaminopyrrolnitrin synthase PrnB
DM992_38835	17.303408	1934.3674	-6.8047	3.13E-157	3.65E-154	prnA	tryptophan 7-halogenase
DM992_38840	5.827248	59.39327	-3.3494	3.68E-15	3.30E-13	--	hypothetical protein
DM992_38845	35.566067	116.63055	-1.7134	3.06E-07	1.23E-05	--	LysE family translocator
DM992_38855	21.39013	56.426298	-1.3994	6.14E-05	0.001518	--	IS110 family transposase
DM992_38890	101.27829	210.06578	-1.0525	3.11E-06	0.0001056	--	AraC family transcriptional regulator
DM992_38895	3.5941347	21.72158	-2.5954	8.83E-06	0.0002698	--	MarR family transcriptional regulator
DM992_38900	41.378523	199.74849	-2.2712	5.16E-20	6.38E-18	--	LysR family transcriptional regulator
DM992_38905	255.89993	1797.2215	-2.8121	8.53E-49	2.58E-46	gdsA	histidine phosphatase family protein
DM992_38910	139.25106	1345.1196	-3.272	1.48E-30	2.63E-28	gdsB	[acyl-carrier-protein] S-malonyltransferase
DM992_38915	114.65945	537.47915	-2.2289	1.37E-23	1.97E-21	gdsC	acyl carrier protein
DM992_38920	43.233058	630.57174	-3.8665	2.97E-22	3.98E-20	gdsD	asparagine synthase (glutamine-hydrolyzing)
DM992_38925	161.4317	2363.967	-3.8722	1.43E-82	7.29E-80	gdsE	polyketide synthase
DM992_38930	525.20051	5225.567	-3.3146	8.21E-70	3.72E-67	gdsF	hypothetical protein
DM992_38935	47.419258	445.24108	-3.231	6.57E-45	1.63E-42	gdsG	hypothetical protein
DM992_38940	35.181821	349.68146	-3.3131	1.12E-41	2.47E-39	gdsH	NADP-dependent oxidoreductase
DM992_39405	31.594335	139.43271	-2.1418	1.46E-15	1.35E-13	--	fimbrial biogenesis outer membrane usher protein
DM992_39410	22.040799	117.96149	-2.4201	5.20E-16	4.94E-14	--	molecular chaperone

DM992_39415	588.02431	3126.5014	-2.4106	1.83E-15	1.68E-13	--	spore coat protein
DM992_39620	25.096987	52.324277	-1.06	0.002451	0.035461	--	hypothetical protein
DM992_39625	166.07925	341.81371	-1.0413	1.64E-07	6.86E-06	--	hypothetical protein
DM992_39635	79.924754	176.0295	-1.1391	1.91E-06	6.78E-05	--	cell division protein FtsH
DM992_39645	192.05838	479.34516	-1.3195	3.10E-11	2.04E-09	--	OsmY domain-containing protein
DM992_39650	31.807822	81.429967	-1.3562	5.62E-05	0.0013992	--	hypothetical protein
DM992_39680	80.37317	191.5164	-1.2527	8.47E-08	3.73E-06	--	nitroreductase
DM992_39695	285.76776	608.42704	-1.0902	5.11E-09	2.64E-07	--	hypothetical protein
DM992_39710	116.22545	251.00758	-1.1108	5.03E-07	1.97E-05	--	Hsp20/alpha crystallin family protein
DM992_39715	164.11091	559.70469	-1.77	2.06E-19	2.40E-17	--	DUF1488 domain-containing protein
DM992_39720	75.456516	183.61473	-1.283	6.38E-07	2.44E-05	--	universal stress protein
DM992_39735	417.1367	1044.23	-1.3238	2.24E-13	1.78E-11	--	fatty acyl-AMP ligase
DM992_39745	147.75845	515.66869	-1.8032	1.51E-11	1.05E-09	--	hypothetical protein
DM992_39750	242.29014	495.44646	-1.032	1.03E-07	4.46E-06	--	SagB/ThcOx family dehydrogenase
DM992_39755	177.90389	404.29703	-1.1843	2.56E-09	1.40E-07	--	hypothetical protein
DM992_39765	26.148963	56.831067	-1.1199	0.000712	0.012632	--	potassium-transporting ATPase subunit C
DM992_39785	1263.9597	2586.8886	-1.0333	6.08E-09	3.08E-07	--	acetate--CoA ligase
DM992_39790	376.25648	782.70356	-1.0567	2.27E-08	1.10E-06	pdhA	pyruvate dehydrogenase (acetyl-transferring) E1 component subunit alpha
DM992_39795	347.90595	854.12153	-1.2957	2.75E-09	1.49E-07	--	alpha-ketoacid dehydrogenase subunit beta
DM992_39815	142.42606	303.84236	-1.0931	1.72E-05	0.0004942	--	phosphoribosyltransferase
DM992_39835	95.778956	236.78486	-1.3058	0.001649	0.025637	--	hypothetical protein
DM992_39840	39.360804	134.83211	-1.7763	3.28E-09	1.75E-07	--	nicotinate phosphoribosyltransferase
DM992_39965	10.327339	35.748793	-1.7914	4.88E-05	0.0012415	--	hypothetical protein
DM992_39970	79.800177	168.51784	-1.0784	3.28E-05	0.0008754	--	Crp/Fnr family transcriptional regulator
DM992_40000	200.99346	426.92353	-1.0868	3.35E-08	1.58E-06	--	hypothetical protein
DM992_40005	292.87356	678.89605	-1.2129	2.33E-10	1.42E-08	--	ABC transporter ATP-binding protein
DM992_40010	401.69298	1009.2823	-1.3292	2.68E-07	1.09E-05	--	hypothetical protein
DM992_40015	2370.0889	5413.7602	-1.1917	2.54E-12	1.87E-10	--	hypothetical protein
DM992_40265	315.33629	723.05197	-1.1972	1.44E-10	9.06E-09	--	AMP-dependent synthetase
DM992_40280	202.00724	416.23243	-1.043	4.04E-05	0.0010462	glgA	glycogen synthase GlgA
DM992_40370	638.67495	1297.5984	-1.0227	4.55E-08	2.10E-06	--	hypothetical protein
DM992_40375	2140.0101	4342.494	-1.0209	2.30E-09	1.27E-07	--	hypothetical protein

DM992_40410	151.64065	323.98357	-1.0953	1.40E-07	5.98E-06	--	DNA-binding protein
DM992_40665	207.70589	646.58715	-1.6383	5.65E-14	4.61E-12	--	hypothetical protein
DM992_40690	14.238096	36.839457	-1.3715	0.00156	0.024474	--	DNA-binding response regulator

Note: The colors of log2 fold change column represents the transcription level of the genes in Δ*bysR* relative to WT based on the RNA-seq results.

Supplementary Table S3 The KEGG pathway enrichment results

Term	ID	Input number	Background number	P-Value	Corrected P-Value	Input
Flagellar assembly	bct02040	20	38	5.77E-11	3.69E-09	DM992_03030|DM992_03080|DM992_04500|DM992_04720|DM992_04535|DM992_04785|DM992_04765|DM992_03075<br>DM992_04720|DM992_04535|DM992_04785|DM992_03020|DM992_04750|DM992_03725|DM992_04725|DM992_04790<br>DM992_03020|DM992_04750|DM992_03725|DM992_04510|DM992_04520|DM992_29375|DM992_03015|DM992_04760<br>DM992_04745|DM992_04745|DM992_02935|DM992_02935|DM992_04765|DM992_03075|DM992_04725|DM992_04790<br>DM992_04510|DM992_04520|DM992_29375|DM992_03015|DM992_04760
Bacterial chemotaxis	bct02030	19	36	1.71E-10	5.46E-09	DM992_02990|DM992_03020|DM992_03005|DM992_24480|DM992_02980|DM992_05700|DM992_04625|DM992_29375<br>DM992_04520|DM992_03725|DM992_02985|DM992_02975|DM992_31425|DM992_03000|DM992_18385|DM992_03010<br>DM992_03015|DM992_02970|DM992_24475
Two-component system	bct02020	26	137	4.11E-06	8.77E-05	DM992_39765|DM992_33890|DM992_03020|DM992_03000|DM992_03005|DM992_13970|DM992_24475|DM992_02990<br>DM992_05790|DM992_05795|DM992_03010|DM992_03030|DM992_06085|DM992_03075|DM992_24480|DM992_02980<br>DM992_40690|DM992_13985|DM992_05700|DM992_12640|DM992_29375|DM992_31425|DM992_18385|DM992_33455<br>DM992_12410|DM992_02975
Carbon metabolism	bct01200	14	127	0.045771221	0.562894776	DM992_20510|DM992_20505|DM992_06085|DM992_06090|DM992_21865|DM992_39795<br>DM992_39790|DM992_10125|DM992_39830|DM992_39785|DM992_12350|DM992_03180|DM992_12360|DM992_20595
Chloroalkane and chloroalkene degradation	bct00625	3	11	0.048891576	0.562894776	DM992_32870|DM992_12400|DM992_12350
Fatty acid biosynthesis	bct00061	4	21	0.061332921	0.562894776	DM992_38910|DM992_40265|DM992_21850|DM992_33045
Methane metabolism	bct00680	5	31	0.064043202	0.562894776	DM992_20510|DM992_20505|DM992_20595|DM992_39785|DM992_12350
Glycolysis / Gluconeogenesis	bct00010	5	33	0.077394647	0.562894776	DM992_32870|DM992_39795|DM992_12400|DM992_39790|DM992_39785
Pyruvate metabolism	bct00620	7	56	0.085271629	0.562894776	DM992_06085|DM992_21865|DM992_39795|DM992_39790|DM992_27175|DM992_39785|DM992_20595
Glyoxylate and dicarboxylate metabolism	bct00630	8	68	0.087952309	0.562894776	DM992_20510|DM992_20505|DM992_06085|DM992_06090|DM992_10125|DM992_03180|DM992_12360|DM992_03185
Pentose and glucuronate interconversions	bct00040	3	18	0.131263488	0.662833539	DM992_34385|DM992_30755|DM992_29905
Fatty acid metabolism	bct01212	5	40	0.134638063	0.662833539	DM992_38910|DM992_06085|DM992_40265|DM992_21850|DM992_33045
Amino sugar and nucleotide sugar metabolism	bct00520	5	40	0.134638063	0.662833539	DM992_17170|DM992_30755|DM992_30725|DM992_29905|DM992_29895
Fatty acid degradation	bct00071	4	30	0.148174712	0.677370114	DM992_32870|DM992_06085|DM992_12400|DM992_40265
Nicotinate and nicotinamide metabolism	bct00760	3	20	0.160295325	0.683926718	DM992_39840|DM992_20555|DM992_39825
Galactose metabolism	bct00052	2	11	0.184464944	0.737859776	DM992_17170|DM992_30755
Alanine, aspartate and glutamate metabolism	bct00250	4	38	0.249116096	0.883205108	DM992_38920|DM992_33335|DM992_19985|DM992_21725
Quorum sensing	bct02024	10	120	0.263278825	0.883205108	DM992_03030|DM992_40690|DM992_27160|DM992_02885|DM992_33455|DM992_27165|DM992_27170|DM992_33845<br>DM992_12305|DM992_40265
Butanoate metabolism	bct00650	5	53	0.274558578	0.883205108	DM992_33335|DM992_12355|DM992_06085|DM992_06090|DM992_12360
RNA degradation	bct03018	2	15	0.276001596	0.883205108	DM992_35840|DM992_20465
Pentose phosphate pathway	bct00030	3	28	0.289807091	0.88322161	DM992_17795|DM992_39830|DM992_35780
Nitrogen metabolism	bct00910	3	29	0.306759148	0.892390248	DM992_02890|DM992_11935|DM992_31250
beta-Lactam resistance	bct01501	3	34	0.391411443	0.964259232	DM992_12305|DM992_13960|DM992_13970
Starch and sucrose metabolism	bct00500	2	21	0.411062415	0.964259232	DM992_30755|DM992_29905
Fructose and mannose metabolism	bct00051	2	22	0.432478931	0.964259232	DM992_30725|DM992_29895
Degradation of aromatic compounds	bct01220	4	56	0.498183912	0.964259232	DM992_32870|DM992_33030|DM992_12400|DM992_34755
Biosynthesis of unsaturated fatty acids	bct01040	1	11	0.528486316	0.964259232	DM992_21850
Sulfur relay system	bct04122	1	11	0.528486316	0.964259232	DM992_04415
Oxidative phosphorylation	bct00190	4	60	0.549852983	0.964259232	DM992_21875|DM992_05790|DM992_21870|DM992_05795
Cationic antimicrobial peptide (CAMP) resistance	bct01503	1	12	0.557154764	0.964259232	DM992_13970
Steroid degradation	bct00984	1	12	0.557154764	0.964259232	DM992_38820

Propanoate metabolism	bct00640	3	46	0.578517418	0.964259232	DM992_33335|DM992_06085|DM992_39785
Carbon fixation in photosynthetic organisms	bct00710	1	13	0.584085092	0.964259232	DM992_21865
Tyrosine metabolism	bct00350	2	30	0.586840792	0.964259232	DM992_32870|DM992_12400
Citrate cycle (TCA cycle)	bct00020	2	30	0.586840792	0.964259232	DM992_39795|DM992_39790
Synthesis and degradation of ketone bodies	bct00072	1	14	0.609382378	0.964259232	DM992_06085
Arginine and proline metabolism	bct00330	3	50	0.632396729	0.964259232	DM992_04885|DM992_20325|DM992_20335
Biotin metabolism	bct00780	1	16	0.655466855	0.964259232	DM992_21850
Selenocompound metabolism	bct00450	1	16	0.655466855	0.964259232	DM992_24060
One carbon pool by folate	bct00670	1	17	0.676434031	0.964259232	DM992_03180
Benzoate degradation	bct00362	3	55	0.692773142	0.964259232	DM992_06085|DM992_33030|DM992_34755
Cysteine and methionine metabolism	bct00270	2	37	0.694615486	0.964259232	DM992_24060|DM992_02915
Microbial metabolism in diverse environments	bct01120	20	341	0.700898341	0.964259232	DM992_20510|DM992_32870|DM992_34755|DM992_33030|DM992_12410|DM992_06090|DM992_21865|DM992_12360 DM992_39795|DM992_39790|DM992_12400|DM992_10125|DM992_33335|DM992_06085|DM992_20505|DM992_39830 DM992_38820|DM992_20595|DM992_39785|DM992_12350
Folate biosynthesis	bct00790	1	19	0.714628255	0.964259232	DM992_04415
Terpenoid backbone biosynthesis	bct00900	1	19	0.714628255	0.964259232	DM992_06085
Valine, leucine and isoleucine biosynthesis	bct00290	1	21	0.74832598	0.964259232	DM992_27175
beta-Alanine metabolism	bct00410	1	21	0.74832598	0.964259232	DM992_33335
Porphyrin and chlorophyll metabolism	bct00860	2	42	0.7565512	0.964259232	DM992_17040|DM992_33360
Ascorbate and aldarate metabolism	bct00053	1	22	0.763656142	0.964259232	DM992_29905
Metabolic pathways	bct01100	53	893	0.766075921	0.964259232	DM992_21725|DM992_03180|DM992_34755|DM992_17170|DM992_40265|DM992_33045|DM992_39840|DM992_19985 DM992_20510|DM992_32870|DM992_04415|DM992_05790|DM992_34385|DM992_20325|DM992_24060|DM992_04885 DM992_21870|DM992_03185|DM992_20595|DM992_33030|DM992_21850|DM992_39795|DM992_35775|DM992_39790 DM992_38820|DM992_33335|DM992_12400|DM992_39830|DM992_12350|DM992_20555|DM992_30725|DM992_20505 DM992_30755|DM992_12410|DM992_27175|DM992_33360|DM992_39825|DM992_39785|DM992_17795|DM992_17790 DM992_21865|DM992_38910|DM992_02915|DM992_29895|DM992_17040|DM992_21875|DM992_29905|DM992_05795 DM992_06085|DM992_35780|DM992_38920|DM992_20335|DM992_10125
Bacterial secretion system	bct03070	2	45	0.788274095	0.964259232	DM992_13970|DM992_31485
Lysine degradation	bct00310	1	25	0.804281894	0.964259232	DM992_06085
Pantothenate and CoA biosynthesis	bct00770	1	26	0.816212408	0.964259232	DM992_33065
Pyrimidine metabolism	bct00240	2	48	0.816306954	0.964259232	DM992_35775|DM992_17790
Glycine, serine and threonine metabolism	bct00260	2	52	0.848513321	0.964259232	DM992_03180|DM992_03185
Tryptophan metabolism	bct00380	1	31	0.865827298	0.964259232	DM992_06085
Biosynthesis of secondary metabolites	bct01110	17	334	0.868028958	0.964259232	DM992_32870|DM992_06085|DM992_38920|DM992_39795|DM992_03180|DM992_39790|DM992_33360|DM992_03185 DM992_27175|DM992_10125|DM992_24060|DM992_21725|DM992_12400|DM992_39830|DM992_39785|DM992_17040 DM992_29895
Purine metabolism	bct00230	3	80	0.887846092	0.964259232	DM992_35775|DM992_17790|DM992_39830
2-Oxocarboxylic acid metabolism	bct01210	1	34	0.88892648	0.964259232	DM992_27175
Valine, leucine and isoleucine degradation	bct00280	1	43	0.937025003	0.988252759	DM992_06085
Sulfur metabolism	bct00920	1	49	0.956884033	0.988252759	DM992_12410
Biosynthesis of antibiotics	bct01130	10	248	0.95736986	0.988252759	DM992_32870|DM992_06085|DM992_39795|DM992_30755|DM992_39790|DM992_12400|DM992_39830|DM992_39785 DM992_03180|DM992_03185
ABC transporters	bct02010	7	236	0.993402699	0.995107464	DM992_33395|DM992_13985|DM992_02885|DM992_04625|DM992_04880|DM992_04870|DM992_04875
Biosynthesis of amino acids	bct01230	3	145	0.995107464	0.995107464	DM992_24060|DM992_39830|DM992_27175

Supplementary Table S4 The enriched peaks identified by DAP-seq

<table>
<tr><th>PeakID</th><th>Chr:Start-End</th><th>Annotation</th><th>Distance to TSS</th><th>Nearest PromoterID</th><th>venn</th><th>BYSR-1_peakid</th><th>BYSR-1_fold enrichment</th><th>BYSR-1_-log10 (pvalue)</th><th>BYSR-1_-log10 (qvalue)</th><th>BYSR-1_peak Chr:Start-End</th><th>BYSR-1_summit_site</th><th>BYSR-2_peakid</th><th>BYSR-2_fold enrichment</th><th>BYSR-2_-log10 (pvalue)</th><th>BYSR-2_-log10 (qvalue)</th><th>BYSR-2_peak Chr:Start-End</th><th>BYSR-2_summit_site</th><th>geneid</th><th>description</th></tr>
<tr><td>Merged-Plas2-91922-2</td><td>Plas2:91771-92076</td><td>promoter</td><td>-651</td><td>DM992_39765</td><td>1&2</td><td>BYSR-1_peak_783</td><td>1.32879</td><td>4.92544</td><td>2.91807</td><td>Plas2:91788-92055</td><td>92042</td><td>BYSR-2_peak_667</td><td>1.44405</td><td>7.13485</td><td>4.41386</td><td>Plas2:91771-92076</td><td>91862</td><td>DM992_39765</td><td>WP_059493067.1|MULTISPECIES: potassium-transporting ATPase subunit KdpC [Burkholderia cepacia complex]</td></tr>
<tr><td>Merged-Plas2-56627-2</td><td>Plas2:56300-57049</td><td>promoter</td><td>271</td><td>DM992_39560</td><td>1&2</td><td>BYSR-1_peak_779</td><td>1.37602</td><td>6.41478</td><td>4.00161</td><td>Plas2:56300-56726</td><td>56690</td><td>BYSR-2_peak_663</td><td>1.3389</td><td>4.88581</td><td>2.80657</td><td>Plas2:56433-57049</td><td>56490</td><td>DM992_39560</td><td>WP_060311642.1|MULTISPECIES: DUF1851 domain-containing protein [Burkholderia cepacia complex]</td></tr>
<tr><td>Merged-Plas2-54375-2</td><td>Plas2:54181-54595</td><td>promoter</td><td>-1564</td><td>DM992_39555</td><td>1&2</td><td>BYSR-1_peak_778</td><td>1.27218</td><td>4.05076</td><td>2.29122</td><td>Plas2:54181-54466</td><td>54203</td><td>BYSR-2_peak_661</td><td>1.23266</td><td>2.89738</td><td>1.41092</td><td>Plas2:54261-54595</td><td>54285</td><td>DM992_39555</td><td>WP_175036909.1|hypothetical protein [Burkholderia contaminans]</td></tr>
<tr><td>Merged-Plas2-49273-4</td><td>Plas2:48131-50310</td><td>promoter</td><td>-96</td><td>DM992_39535</td><td>1&2</td><td>BYSR-1_peak_777</td><td>1.4234</td><td>8.35612</td><td>5.42345</td><td>Plas2:48131-50310</td><td>49853</td><td>BYSR-2_peak_658, BYSR-2_peak_659, BYSR-2_peak_660</td><td>-</td><td>-</td><td>-</td><td>-</td><td>-</td><td>DM992_39535</td><td>WP_107314531.1|carbon-nitrogen hydrolase family protein [Burkholderia metallica]</td></tr>
<tr><td>Merged-Plas2-46946-2</td><td>Plas2:46222-47508</td><td>promoter</td><td>109</td><td>DM992_39520</td><td>1&2</td><td>BYSR-1_peak_776</td><td>1.32725</td><td>5.24378</td><td>3.14964</td><td>Plas2:46222-47450</td><td>46830</td><td>BYSR-2_peak_657</td><td>1.40194</td><td>6.39077</td><td>3.88848</td><td>Plas2:46605-47508</td><td>47247</td><td>DM992_39520</td><td>WP_107314542.1|helix-turn-helix domain-containing protein [Burkholderia metallica]</td></tr>
<tr><td>Merged-Plas2-45496-2</td><td>Plas2:45189-45803</td><td>promoter-TSS (JP2270_GM003485_rna)</td><td>617</td><td>DM992_39515</td><td>1&2</td><td>BYSR-1_peak_775</td><td>1.36228</td><td>6.54088</td><td>4.09499</td><td>Plas2:45435-45803</td><td>45632</td><td>BYSR-2_peak_655</td><td>1.32491</td><td>4.87935</td><td>2.8018</td><td>Plas2:45189-45560</td><td>45539</td><td>DM992_39515</td><td>WP_105393594.1|response regulator transcription factor [Burkholderia cepacia]</td></tr>
<tr><td>Merged-Plas2-44297-3</td><td>Plas2:43488-45139</td><td>promoter</td><td>-34</td><td>DM992_39510</td><td>1&2</td><td>BYSR-1_peak_774, BYSR-1_peak_773</td><td>-</td><td>-</td><td>-</td><td>-</td><td>-</td><td>BYSR-2_peak_654</td><td>1.25142</td><td>3.45475</td><td>1.79347</td><td>Plas2:43488-44883</td><td>44808</td><td>DM992_39510</td><td>AKM04415.1|transposase [Burkholderia pyrrocinia]</td></tr>
<tr><td>Merged-Plas2-414163-2</td><td>Plas2:413893-414409</td><td>promoter</td><td>221</td><td>DM992_41355</td><td>1&2</td><td>BYSR-1_peak_878</td><td>1.35799</td><td>6.64298</td><td>4.16807</td><td>Plas2:413893-414376</td><td>414214</td><td>BYSR-2_peak_744</td><td>1.31048</td><td>4.72486</td><td>2.6902</td><td>Plas2:413975-414409</td><td>414272</td><td>DM992_41355</td><td>WP_124469842.1|porin [Burkholderia cepacia]</td></tr>
<tr><td>Merged-Plas2-412963-2</td><td>Plas2:412280-413741</td><td>promoter</td><td>284</td><td>DM992_41350</td><td>1&2</td><td>BYSR-1_peak_877</td><td>1.41598</td><td>8.41747</td><td>5.46672</td><td>Plas2:412280-413532</td><td>413180</td><td>BYSR-2_peak_743</td><td>1.50044</td><td>10.354</td><td>6.58093</td><td>Plas2:412301-413741</td><td>413361</td><td>DM992_41350</td><td>WP_069263648.1|hypothetical protein [Burkholderia vietnamiensis]</td></tr>
<tr><td>Merged-Plas2-411020-2</td><td>Plas2:410581-411552</td><td>promoter</td><td>453</td><td>DM992_41340</td><td>1&2</td><td>BYSR-1_peak_876</td><td>1.29921</td><td>4.66543</td><td>2.73105</td><td>Plas2:410769-411552</td><td>410947</td><td>BYSR-2_peak_742</td><td>1.24048</td><td>3.19676</td><td>1.61764</td><td>Plas2:410581-411178</td><td>410596</td><td>DM992_41340</td><td>WP_174393665.1|helix-turn-helix domain-containing protein [Burkholderia cepacia]</td></tr>
<tr><td>Merged-Plas2-40729-2</td><td>Plas2:40331-41044</td><td>promoter</td><td>-128</td><td>DM992_39500</td><td>1&2</td><td>BYSR-1_peak_772</td><td>1.25095</td><td>3.78249</td><td>2.10244</td><td>Plas2:40331-41044</td><td>40605</td><td>BYSR-2_peak_653</td><td>1.29852</td><td>4.33464</td><td>2.41109</td><td>Plas2:40518-41024</td><td>40672</td><td>DM992_39500</td><td>WP_174912115.1|MULTISPECIES: hypothetical protein [Burkholderia cepacia complex]</td></tr>
<tr><td>Merged-Plas2-398819-2</td><td>Plas2:398702-398936</td><td>promoter</td><td>80</td><td>DM992_41280</td><td>1&2</td><td>BYSR-1_peak_875</td><td>1.39494</td><td>6.95562</td><td>4.39872</td><td>Plas2:398705-398934</td><td>398878</td><td>BYSR-2_peak_740</td><td>1.30463</td><td>4.38074</td><td>2.44514</td><td>Plas2:398702-398936</td><td>398799</td><td>DM992_41280</td><td>WP_155642184.1|IS481 family transposase [Burkholderia cepacia]</td></tr>
<tr><td>Merged-Plas2-396608-2</td><td>Plas2:396288-396861</td><td>promoter</td><td>51</td><td>DM992_41275</td><td>1&2</td><td>BYSR-1_peak_874</td><td>1.30379</td><td>5.46922</td><td>3.31114</td><td>Plas2:396288-396861</td><td>396524</td><td>BYSR-2_peak_739</td><td>1.32878</td><td>5.46152</td><td>3.2197</td><td>Plas2:396533-396752</td><td>396651</td><td>DM992_41275</td><td>WP_091925906.1|MULTISPECIES: hypothetical protein [Burkholderia]</td></tr>
<tr><td>Merged-Plas2-393551-2</td><td>Plas2:393275-393838</td><td>promoter</td><td>182</td><td>DM992_41255</td><td>1&2</td><td>BYSR-1_peak_873</td><td>1.42571</td><td>8.19462</td><td>5.30688</td><td>Plas2:393275-393809</td><td>393481</td><td>BYSR-2_peak_736</td><td>1.44708</td><td>7.74442</td><td>4.83297</td><td>Plas2:393285-393838</td><td>393492</td><td>DM992_41255</td><td>WP_081070358.1|transposase [Burkholderia cepacia]</td></tr>
<tr><td>Merged-Plas2-390984-2</td><td>Plas2:390766-391172</td><td>promoter</td><td>-6</td><td>DM992_41240</td><td>1&2</td><td>BYSR-1_peak_872</td><td>1.26072</td><td>3.93742</td><td>2.21116</td><td>Plas2:390766-391135</td><td>390776</td><td>BYSR-2_peak_735</td><td>1.24184</td><td>3.32307</td><td>1.70254</td><td>Plas2:390866-391172</td><td>391048</td><td>DM992_41240</td><td>WP_175721204.1|LysR family transcriptional regulator [Burkholderia anthina]</td></tr>
<tr><td>Merged-Plas2-385235-2</td><td>Plas2:384969-385687</td><td>promoter</td><td>-124</td><td>DM992_41220</td><td>1&2</td><td>BYSR-1_peak_870</td><td>1.28467</td><td>4.703</td><td>2.75848</td><td>Plas2:385087-385687</td><td>385586</td><td>BYSR-2_peak_734</td><td>1.31774</td><td>4.86064</td><td>2.78843</td><td>Plas2:384969-385198</td><td>385093</td><td>DM992_41220</td><td>WP_175889413.1|NAD-dependent epimerase/dehydratase family protein [Burkholderia cepacia]</td></tr>
<tr><td>Merged-Plas2-370441-4</td><td>Plas2:369339-371414</td><td>promoter</td><td>145</td><td>DM992_41150</td><td>1&2</td><td>BYSR-1_peak_866, BYSR-1_peak_865</td><td>-</td><td>-</td><td>-</td><td>-</td><td>-</td><td>BYSR-2_peak_731, BYSR-2_peak_732</td><td>-</td><td>-</td><td>-</td><td>-</td><td>-</td><td>DM992_41150</td><td>WP_175889426.1|SDR family oxidoreductase [Burkholderia cepacia]</td></tr>
<tr><td>Merged-Plas2-366639-2</td><td>Plas2:366394-367090</td><td>promoter</td><td>-319</td><td>DM992_41125</td><td>1&2</td><td>BYSR-1_peak_864</td><td>1.4283</td><td>8.99685</td><td>5.90494</td><td>Plas2:366409-367090</td><td>366930</td><td>BYSR-2_peak_729</td><td>1.39727</td><td>6.80948</td><td>4.18671</td><td>Plas2:366394-366665</td><td>366508</td><td>DM992_41125</td><td>WP_175889429.1|FAD-dependent oxidoreductase [Burkholderia cepacia]</td></tr>
<tr><td>Merged-Plas2-365556-2</td><td>Plas2:365325-365817</td><td>promoter</td><td>-705</td><td>DM992_41120</td><td>1&2</td><td>BYSR-1_peak_863</td><td>1.37635</td><td>7.33243</td><td>4.67535</td><td>Plas2:365325-365683</td><td>365484</td><td>BYSR-2_peak_728</td><td>1.28853</td><td>4.31689</td><td>2.39834</td><td>Plas2:365400-365817</td><td>365489</td><td>DM992_41120</td><td>ABB08506.1|Short-chain dehydrogenase/reductase SDR [Burkholderia lata]</td></tr>
<tr><td>Merged-Plas2-363376-3</td><td>Plas2:362530-364788</td><td>promoter</td><td>-365</td><td>DM992_41110</td><td>1&2</td><td>BYSR-1_peak_862</td><td>1.42411</td><td>8.57477</td><td>5.58656</td><td>Plas2:362530-364788</td><td>363361</td><td>BYSR-2_peak_726, BYSR-2_peak_727</td><td>-</td><td>-</td><td>-</td><td>-</td><td>-</td><td>DM992_41110</td><td>WP_175889430.1|nuclear transport factor 2 family protein [Burkholderia cepacia]</td></tr>
<tr><td>Merged-Plas2-361544-2</td><td>Plas2:361225-362196</td><td>promoter</td><td>497</td><td>DM992_41100</td><td>1&2</td><td>BYSR-1_peak_861</td><td>1.32322</td><td>5.99929</td><td>3.69864</td><td>Plas2:361284-362196</td><td>362038</td><td>BYSR-2_peak_725</td><td>1.42862</td><td>7.842</td><td>4.8977</td><td>Plas2:361225-361474</td><td>361348</td><td>DM992_41100</td><td>WP_175721217.1|TetR/AcrR family transcriptional regulator [Burkholderia anthina]</td></tr>
<tr><td>Merged-Plas2-352597-2</td><td>Plas2:352132-352957</td><td>promoter</td><td>467</td><td>DM992_41045</td><td>1&2</td><td>BYSR-1_peak_858</td><td>1.25589</td><td>3.80539</td><td>2.11927</td><td>Plas2:352569-352957</td><td>352910</td><td>BYSR-2_peak_724</td><td>1.31643</td><td>4.84905</td><td>2.77931</td><td>Plas2:352132-352730</td><td>352598</td><td>DM992_41045</td><td>WP_155687443.1|hypothetical protein [Burkholderia cepacia]</td></tr>
<tr><td>Merged-Plas2-348610-3</td><td>Plas2:347965-349180</td><td>promoter</td><td>-74</td><td>DM992_41010</td><td>1&2</td><td>BYSR-1_peak_857, BYSR-1_peak_856</td><td>-</td><td>-</td><td>-</td><td>-</td><td>-</td><td>BYSR-2_peak_722</td><td>1.31886</td><td>5.20304</td><td>3.03673</td><td>Plas2:347965-349157</td><td>348170</td><td>DM992_41010</td><td>WP_059896624.1|site-specific integrase [Burkholderia vietnamiensis]</td></tr>
<tr><td>Merged-Plas2-343011-2</td><td>Plas2:342446-343743</td><td>promoter</td><td>-507</td><td>DM992_40980</td><td>1&2</td><td>BYSR-1_peak_853</td><td>1.5048</td><td>11.4855</td><td>7.78963</td><td>Plas2:342446-343743</td><td>342740</td><td>BYSR-2_peak_719</td><td>1.49561</td><td>10.4688</td><td>6.65147</td><td>Plas2:342453-343404</td><td>342807</td><td>DM992_40980</td><td>WP_175043636.1|transporter substrate-binding domain-containing protein [Burkholderia lata]</td></tr>
<tr><td>Merged-Plas2-341983-2</td><td>Plas2:341786-342223</td><td>promoter</td><td>-257</td><td>DM992_40975</td><td>1&2</td><td>BYSR-1_peak_852</td><td>1.28248</td><td>4.74441</td><td>2.7893</td><td>Plas2:341786-342223</td><td>341952</td><td>BYSR-2_peak_718</td><td>1.35239</td><td>5.99428</td><td>3.60408</td><td>Plas2:341809-342114</td><td>341963</td><td>DM992_40975</td><td>KAF1035283.1|Outer membrane porin protein [Burkholderia lata]</td></tr>
<tr><td>Merged-Plas2-339821-3</td><td>Plas2:339129-340939</td><td>TTS (JP2270_GM003792_rna)</td><td>594</td><td>DM992_40970</td><td>1&2</td><td>BYSR-1_peak_851, BYSR-1_peak_850</td><td>-</td><td>-</td><td>-</td><td>-</td><td>-</td><td>BYSR-2_peak_717</td><td>1.41835</td><td>7.85116</td><td>4.90299</td><td>Plas2:339160-340666</td><td>340220</td><td>DM992_40970</td><td>WP_175802407.1|LysR family transcriptional regulator [Burkholderia anthina]</td></tr>
<tr><td>Merged-Plas2-334160-2</td><td>Plas2:333841-334595</td><td>promoter-TSS (JP2270_GM003790_rna)</td><td>590</td><td>DM992_40950</td><td>1&2</td><td>BYSR-1_peak_849</td><td>1.33863</td><td>5.67208</td><td>3.45936</td><td>Plas2:333841-334595</td><td>334022</td><td>BYSR-2_peak_716</td><td>1.34078</td><td>5.15412</td><td>2.99964</td><td>Plas2:333883-334321</td><td>334039</td><td>DM992_40950</td><td>WP_175804297.1|site-specific integrase [Burkholderia cenocepacia]</td></tr>
<tr><td>Merged-Plas2-324582-2</td><td>Plas2:324036-324979</td><td>promoter</td><td>-19</td><td>DM992_40925</td><td>1&2</td><td>BYSR-1_peak_846</td><td>1.38744</td><td>7.48722</td><td>4.78855</td><td>Plas2:324036-324708</td><td>324129</td><td>BYSR-2_peak_715</td><td>1.41228</td><td>7.38259</td><td>4.58481</td><td>Plas2:324605-324979</td><td>324780</td><td>DM992_40925</td><td>WP_105392915.1|MULTISPECIES: hypothetical protein [Burkholderia]</td></tr>
<tr><td>Merged-Plas2-322499-2</td><td>Plas2:322161-322841</td><td>promoter</td><td>197</td><td>DM992_40920</td><td>1&2</td><td>BYSR-1_peak_845</td><td>1.32775</td><td>5.57099</td><td>3.38691</td><td>Plas2:322375-322841</td><td>322738</td><td>BYSR-2_peak_714</td><td>1.25742</td><td>3.72318</td><td>1.97965</td><td>Plas2:322161-322622</td><td>322301</td><td>DM992_40920</td><td>WP_105392914.1|MULTISPECIES: hypothetical protein [Burkholderia]</td></tr>
<tr><td>Merged-Plas2-319588-2</td><td>Plas2:319110-319887</td><td>TTS (JP2270_GM003780_rna)</td><td>1287</td><td>DM992_40905</td><td>1&2</td><td>BYSR-1_peak_844</td><td>1.30977</td><td>5.09063</td><td>3.03851</td><td>Plas2:319110-319887</td><td>319453</td><td>BYSR-2_peak_713</td><td>1.26997</td><td>3.6757</td><td>1.94601</td><td>Plas2:319496-319860</td><td>319806</td><td>DM992_40905</td><td>WP_175927441.1|hypothetical protein [Burkholderia cepacia]</td></tr>
</table>

Merged-Plas2-310234-2	Plas2:310050-310450	promoter	181	DM992_40865	1&2	BYSR-1_peak_842	1.33912	5.43736	3.29045	Plas2:310076-310361	310222	BYSR-2_peak_711	1.49002	8.96786	5.67064	Plas2:310050-310450	310212	DM992_40865	WP_175907302.1|ATP-binding protein [Burkholderia seminalis]
Merged-Plas2-306403-3	Plas2:305869-306932	promoter-TSS (JP2270_GM003768_rna)	676	DM992_40850	1&2	BYSR-1_peak_840	1.4495	8.51223	5.5396	Plas2:306135-306732	306653	BYSR-2_peak_709, BYSR-2_peak_710	-	-	-	-	-	DM992_40850	KWB25139.1|hypothetical protein WL32_06815 [Burkholderia cepacia]
Merged-Plas2-305067-2	Plas2:304810-305317	promoter	-202	DM992_40840	1&2	BYSR-1_peak_839	1.36142	6.44168	4.02205	Plas2:304810-305294	304900	BYSR-2_peak_708	1.46255	8.19988	5.14256	Plas2:304847-305317	305187	DM992_40840	WP_060320187.1|MULTISPECIES: ParB/RepB/Spo0J family partition protein [Burkholderia]
Merged-Plas2-302080-3	Plas2:300902-302998	promoter	-882	DM992_40835	1&2	BYSR-1_peak_838, BYSR-1_peak_837	-	-	-	-	-	BYSR-2_peak_706	1.37535	6.2131	3.75794	Plas2:300927-302789	301054	DM992_40835	WP_175028733.1|RepB family plasmid replication initiator protein [Burkholderia seminalis]
Merged-Plas2-290078-2	Plas2:289670-290534	promoter	-197	DM992_40765	1&2	BYSR-1_peak_832	1.32245	5.46234	3.30884	Plas2:289670-290240	290083	BYSR-2_peak_704	1.43896	8.0788	5.06035	Plas2:289868-290534	290080	DM992_40765	WP_060243457.1|H-NS histone family protein [Burkholderia pseudomultivorans]
Merged-Plas2-27858-2	Plas2:27366-28260	promoter	-290	DM992_39415	1&2	BYSR-1_peak_769	1.28175	4.50631	2.61814	Plas2:27366-28260	27614	BYSR-2_peak_651	1.34536	5.5755	3.30122	Plas2:27562-28244	27614	DM992_39415	WP_080400280.1|spore coat U domain-containing protein [Burkholderia ubonensis]
Merged-Plas2-266656-2	Plas2:266506-266813	promoter	27	DM992_40685	1&2	BYSR-1_peak_826	1.2992	4.7948	2.82376	Plas2:266506-266800	266673	BYSR-2_peak_699	1.37477	5.78682	3.45332	Plas2:266506-266813	266731	DM992_40685	WP_059493067.1|MULTISPECIES: potassium-transporting ATPase subunit KdpC [Burkholderia cepacia complex]
Merged-Plas2-261608-3	Plas2:261047-262212	promoter	111	DM992_40665	1&2	BYSR-1_peak_824,BYSR-1_peak_823	-	-	-	-	-	BYSR-2_peak_698	1.38353	6.57159	4.01758	Plas2:261047-262192	262009	DM992_40665	WP_009691488.1|MULTISPECIES: hypothetical protein [Burkholderia cepacia complex]
Merged-Plas2-254568-2	Plas2:254283-254791	promoter	20	DM992_40620	1&2	BYSR-1_peak_822	1.34052	6.0339	3.7225	Plas2:254479-254720	254630	BYSR-2_peak_696	1.43135	8.03911	5.03168	Plas2:254283-254791	254643	DM992_40620	WP_059474714.1|peptidoglycan-associated lipoprotein Pal [Burkholderia vietnamiensis]
Merged-Plas2-249929-2	Plas2:249754-250097	promoter	119	DM992_40585	1&2	BYSR-1_peak_819	1.31506	4.93001	2.9214	Plas2:249772-250097	250049	BYSR-2_peak_694	1.50823	8.89938	5.6227	Plas2:249754-250093	249827	DM992_40585	KWB15561.1|transposase [Burkholderia cepacia]
Merged-Plas2-246094-2	Plas2:245469-246475	promoter	-210	DM992_40570	1&2	BYSR-1_peak_818	1.42294	7.47439	4.77933	Plas2:245961-246475	246025	BYSR-2_peak_693	1.47794	8.24588	5.17493	Plas2:245469-246472	246168	DM992_40570	WP_175964102.1|alpha/beta hydrolase [Burkholderia pyrrocinia]
Merged-Plas2-242192-2	Plas2:242009-242400	promoter	412	DM992_40550	1&2	BYSR-1_peak_817	1.27937	4.41553	2.55181	Plas2:242009-242257	242093	BYSR-2_peak_692	1.32312	4.87539	2.79887	Plas2:242102-242400	242369	DM992_40550	WP_175715016.1|multidrug transporter [Burkholderia ambifaria]
Merged-Plas2-241445-2	Plas2:241154-241751	promoter	39	DM992_40545	1&2	BYSR-1_peak_816	1.28705	4.73066	2.77922	Plas2:241254-241623	241380	BYSR-2_peak_691	1.46556	9.22387	5.8489	Plas2:241154-241751	241281	DM992_40545	WP_174432864.1|carboxymuconolactone decarboxylase family protein [Burkholderia metallica]
Merged-Plas2-234748-2	Plas2:234467-235187	promoter	96	DM992_40505	1&2	BYSR-1_peak_814	1.26679	3.96651	2.23239	Plas2:234467-235187	234617	BYSR-2_peak_690	1.29339	4.13596	2.27103	Plas2:234488-234853	234617	DM992_40505	WP_175949491.1|hypothetical protein [Burkholderia vietnamiensis]
Merged-Plas2-217451-2	Plas2:217058-218014	promoter	232	DM992_40385	1&2	BYSR-1_peak_811	1.39844	7.04112	4.46225	Plas2:217058-218014	217976	BYSR-2_peak_689	1.26354	3.57187	1.87392	Plas2:217222-217513	217364	DM992_40385	WP_071335614.1|hypothetical protein [Burkholderia contaminans]
Merged-Plas2-209871-2	Plas2:209627-210120	promoter	-56	DM992_40320	1&2	BYSR-1_peak_809	1.33442	5.05803	3.01369	Plas2:209627-210111	210044	BYSR-2_peak_687	1.34164	4.79608	2.74286	Plas2:209628-210120	209750	DM992_40320	WP_175812180.1|cation-translocating P-type ATPase [Burkholderia contaminans]
Merged-Plas2-204462-2	Plas2:204153-204743	promoter	424	DM992_40300	1&2	BYSR-1_peak_807	1.33632	5.89561	3.62074	Plas2:204153-204661	204194	BYSR-2_peak_686	1.33399	5.3073	3.10896	Plas2:204292-204743	204647	DM992_40300	WP_105392878.1|response regulator transcription factor [Burkholderia cepacia]
Merged-Plas2-190921-2	Plas2:190082-191337	promoter	59	DM992_40265	1&2	BYSR-1_peak_804	1.33605	5.94088	3.65495	Plas2:190082-191337	190427	BYSR-2_peak_684	1.28509	4.03325	2.19865	Plas2:191014-191251	191141	DM992_40265	WP_175806738.1|AMP-binding protein [Burkholderia cenocepacia]
Merged-Plas2-189096-2	Plas2:188907-189268	promoter	-114	DM992_40260	1&2	BYSR-1_peak_803	1.29484	4.22009	2.41325	Plas2:188907-189216	188942	BYSR-2_peak_683	1.31141	4.28123	2.37403	Plas2:188993-189268	189114	DM992_40260	WP_088510477.1|AMP-binding protein [Burkholderia ubonensis]
Merged-Plas2-188182-2	Plas2:187938-188598	TTS (JP2270_GM003647_rna)	705	DM992_40260	1&2	BYSR-1_peak_802	1.33507	5.88221	3.61337	Plas2:187987-188598	188087	BYSR-2_peak_682	1.38522	6.54724	4.00187	Plas2:187938-188208	188110	DM992_40260	WP_088510477.1|AMP-binding protein [Burkholderia ubonensis]
Merged-Plas2-182724-2	Plas2:182576-182851	promoter	145	DM992_40240	1&2	BYSR-1_peak_801	1.37947	6.18099	3.82895	Plas2:182624-182851	182751	BYSR-2_peak_680	1.27718	3.43417	1.77897	Plas2:182576-182846	182708	DM992_40240	AVR14377.1|hypothetical protein A8H33_12760 [Burkholderia vietnamiensis]
Merged-Plas2-173376-2	Plas2:173073-173591	exon (JP2270_GM003634_rna, exon 1 of 1)	1957	DM992_40205	1&2	BYSR-1_peak_796	1.25774	3.78606	2.10485	Plas2:173073-173591	173097	BYSR-2_peak_678	1.42482	7.39517	4.59417	Plas2:173298-173544	173425	DM992_40205	OUE42570.1|integrase [Burkholderia territorii]
Merged-Plas2-167060-2	Plas2:166914-167221	promoter	356	DM992_40175	1&2	BYSR-1_peak_795	1.41716	7.45535	4.76456	Plas2:166914-167175	166989	BYSR-2_peak_676	1.47344	7.93263	4.96046	Plas2:166931-167221	167014	DM992_40175	WP_124634223.1|group II intron reverse transcriptase/maturase [Burkholderia cenocepacia]
Merged-Plas2-164128-2	Plas2:163903-164330	promoter	-193	DM992_40145	1&2	BYSR-1_peak_794	1.5689	12.3865	8.50001	Plas2:163903-164307	164040	BYSR-2_peak_675	1.3678	5.49959	3.2489	Plas2:163972-164330	164055	DM992_40145	WP_010099900.1|hypothetical protein [Burkholderia ubonensis]
Merged-Plas2-133151-2	Plas2:132741-133555	promoter	-361	DM992_39985	1&2	BYSR-1_peak_787	1.34684	6.22744	3.8636	Plas2:132741-133333	132903	BYSR-2_peak_671	1.40657	6.56197	4.01111	Plas2:132976-133555	133465	DM992_39985	WP_128841768.1|BON domain-containing protein [Burkholderia catarinensis]
Merged-Plas2-130316-2	Plas2:129420-131042	promoter	-206	DM992_39970	1&2	BYSR-1_peak_786	1.29485	4.30499	2.47288	Plas2:129987-130815	130227	BYSR-2_peak_670	1.51422	9.17619	5.81685	Plas2:129420-131042	130356	DM992_39970	WP_034181435.1|MULTISPECIES: Crp/Fnr family transcriptional regulator [Burkholderia cepacia complex]
Merged-Plas2-111794-2	Plas2:111553-112083	promoter	-76	DM992_39865	1&2	BYSR-1_peak_785	1.42292	7.39924	4.72558	Plas2:111594-112083	111672	BYSR-2_peak_668	1.53125	9.59073	6.09051	Plas2:111553-111948	111659	DM992_39865	WP_105393367.1|hypothetical protein [Burkholderia cepacia]
Merged-Plas1-3811-2	Plas1:3560-4042	promoter	65	DM992_39230	1&2	BYSR-1_peak_760	1.30058	9.72483	6.45354	Plas1:3560-4022	3672	BYSR-2_peak_643	1.31752	9.91313	6.30196	Plas1:3622-4042	3893	DM992_39230	WP_060190439.1|hypothetical protein [Burkholderia diffusa]
Merged-Plas1-14683-2	Plas1:14343-15018	promoter	401	DM992_39290	1&2	BYSR-1_peak_765	1.33892	12.3351	8.46035	Plas1:14343-15018	14506	BYSR-2_peak_646	1.30778	9.35886	5.93715	Plas1:14355-15018	14495	DM992_39290	WP_069236659.1|hypothetical protein [Burkholderia ubonensis]
Merged-Plas1-13801-2	Plas1:13588-14005	promoter	-25	DM992_39280	1&2	BYSR-1_peak_764	1.25599	7.98265	5.15197	Plas1:13588-13989	13893	BYSR-2_peak_645	1.26233	7.64177	4.76459	Plas1:13624-14005	13879	DM992_39280	AOK57664.1|hypothetical protein WM29_00020 [Burkholderia ubonensis]
Merged-Chr3-982359-2	Chr3:982136-982555	promoter	-235	DM992_37235	1&2	BYSR-1_peak_689	1.48422	10.4416	6.9887	Chr3:982229-982555	982352	BYSR-2_peak_579	1.53979	11.3837	7.25474	Chr3:982136-982518	982230	DM992_37235	WP_175748014.1|nitrate reductase subunit alpha [Burkholderia pyrrocinia]
Merged-Chr3-96974-2	Chr3:96550-97263	promoter	-510	DM992_33345	1&2	BYSR-1_peak_594	1.24046	4.00265	2.25887	Chr3:96992-97263	97214	BYSR-2_peak_478	1.31805	5.42243	3.19363	Chr3:96550-97091	97016	DM992_33345	WP_072439727.1|MULTISPECIES: AMP-binding protein [Burkholderia]
Merged-Chr3-957206-2	Chr3:957015-957412	promoter	-162	DM992_37120	1&2	BYSR-1_peak_688	1.2891	4.88595	2.89185	Chr3:957119-957412	957300	BYSR-2_peak_577	1.54434	12.0247	7.66516	Chr3:957015-957280	957137	DM992_37120	WP_175961843.1|RidA family protein [Burkholderia pyrrocinia]

Merged-Chr3-95027-2	Chr3:94791-95275	promoter	35	DM992_33335	1&2	BYSR-1_peak_593	1.2841	4.66365	2.73105	Chr3:94791-95275	95165	BYSR-2_peak_477	1.27485	3.95372	2.1409	Chr3:94860-95185	95179	DM992_33335	WP_072439731.1|MULTISPECIES: diaminobutyrate--2-oxoglutarate transaminase family protein [Burkholderia]
Merged-Chr3-944987-2	Chr3:944610-945373	promoter	-90	DM992_37045	1&2	BYSR-1_peak_687	1.32351	6.03938	3.72577	Chr3:944687-945278	945103	BYSR-2_peak_575	1.37547	6.67004	4.08804	Chr3:944610-945373	945301	DM992_37045	WP_034184565.1|MULTISPECIES: hypothetical protein [Burkholderia cepacia complex]
Merged-Chr3-911811-2	Chr3:911594-912070	promoter	118	DM992_36880	1&2	BYSR-1_peak_685	1.29202	5.18846	3.11116	Chr3:911598-912070	911876	BYSR-2_peak_574	1.3516	6.34718	3.85559	Chr3:911594-911985	911870	DM992_36880	WP_096475765.1|PIN domain nuclease [Burkholderia stabilis]
Merged-Chr3-900137-2	Chr3:899970-900287	promoter	449	DM992_36815	1&2	BYSR-1_peak_683	1.46652	11.8388	8.06598	Chr3:899970-900270	900157	BYSR-2_peak_573	1.33636	6.15595	3.71861	Chr3:900022-900287	900159	DM992_36815	WP_175748086.1|patatin-like phospholipase family protein [Burkholderia pyrrocinia]
Merged-Chr3-898733-2	Chr3:898525-898982	promoter	-926	DM992_36815	1&2	BYSR-1_peak_682	1.33558	6.01549	3.71099	Chr3:898525-898982	898586	BYSR-2_peak_572	1.42585	7.87208	4.91792	Chr3:898553-898875	898770	DM992_36815	WP_175748086.1|patatin-like phospholipase family protein [Burkholderia pyrrocinia]
Merged-Chr3-896590-2	Chr3:896435-896747	promoter	349	DM992_36810	1&2	BYSR-1_peak_681	1.31485	5.8904	3.61693	Chr3:896473-896747	896561	BYSR-2_peak_571	1.35779	6.48501	3.95564	Chr3:896435-896708	896561	DM992_36810	PXX36870.1|adenylate/guanylate cyclase [Burkholderia pyrrocinia]
Merged-Chr3-876074-2	Chr3:875592-876552	promoter-TSS (JP2270_GM007706_rna)	503	DM992_36730	1&2	BYSR-1_peak_679	1.43646	9.80993	6.51811	Chr3:875732-876420	876092	BYSR-2_peak_569	1.4484	9.3246	5.91381	Chr3:875592-876552	876012	DM992_36730	WP_174419649.1|MULTISPECIES: hypothetical protein [Burkholderia cepacia complex]
Merged-Chr3-861222-2	Chr3:860931-861533	promoter	-151	DM992_36655	1&2	BYSR-1_peak_677	1.35125	6.70846	4.21755	Chr3:860931-861486	861322	BYSR-2_peak_567	1.34742	5.786	3.45287	Chr3:860940-861533	861397	DM992_36655	WP_006405208.1|MULTISPECIES: MSMEG_0572 family nitrogen starvation response protein [Burkholderia]
Merged-Chr3-835916-2	Chr3:835597-836416	promoter	-33	DM992_36555	1&2	BYSR-1_peak_676	1.37466	7.28601	4.64082	Chr3:835753-836416	835902	BYSR-2_peak_564	1.21319	2.79363	1.34072	Chr3:835597-835900	835598	DM992_36555	WP_131947596.1|IclR family transcriptional regulator [Burkholderia pyrrocinia]
Merged-Chr3-819252-2	Chr3:819077-819412	promoter	-460	DM992_36495	1&2	BYSR-1_peak_675	1.35475	7.02834	4.45245	Chr3:819077-819412	819199	BYSR-2_peak_563	1.28522	4.56941	2.57856	Chr3:819131-819390	819257	DM992_36495	WP_175748129.1|undecaprenyl-phosphate glucose phosphotransferase [Burkholderia pyrrocinia]
Merged-Chr3-802744-2	Chr3:802244-803078	promoter	-147	DM992_36395	1&2	BYSR-1_peak_673	1.40297	8.23366	5.33389	Chr3:802620-803035	802706	BYSR-2_peak_561	1.46386	9.29145	5.88823	Chr3:802244-803078	802669	DM992_36395	WP_105393474.1|hypothetical protein [Burkholderia cepacia]
Merged-Chr3-801805-2	Chr3:801659-801932	promoter	-102	DM992_36390	1&2	BYSR-1_peak_672	1.27846	4.53632	2.6395	Chr3:801659-801921	801850	BYSR-2_peak_560	1.29562	4.57488	2.58255	Chr3:801709-801932	801822	DM992_36390	WP_131947611.1|hypothetical protein [Burkholderia pyrrocinia]
Merged-Chr3-798380-3	Chr3:797784-798896	promoter	-71	DM992_36380	1&2	BYSR-1_peak_671, BYSR-1_peak_670	-	-	-	-	-	BYSR-2_peak_559	1.38553	6.85567	4.22049	Chr3:798160-798675	798547	DM992_36380	WP_105393471.1|MULTISPECIES: PIN domain nuclease [Burkholderia]
Merged-Chr3-774754-2	Chr3:773523-775974	promoter	257	DM992_36240	1&2	BYSR-1_peak_661	1.49021	10.7768	7.24716	Chr3:773523-775927	774677	BYSR-2_peak_550	1.48054	9.15443	5.80227	Chr3:773595-775974	775162	DM992_36240	WP_175766173.1|hypothetical protein [Burkholderia ambifaria]
Merged-Chr3-742868-2	Chr3:742698-743086	promoter	-184	DM992_36095	1&2	BYSR-1_peak_655	1.28878	4.64812	2.72076	Chr3:742737-743086	742768	BYSR-2_peak_546	1.29189	4.37147	2.43818	Chr3:742698-742951	742798	DM992_36095	KVA55295.1|hypothetical protein WI48_01830 [Burkholderia cepacia]
Merged-Chr3-740349-2	Chr3:740102-740596	promoter	-161	DM992_36080	1&2	BYSR-1_peak_654	1.27211	4.22213	2.41486	Chr3:740139-740596	740448	BYSR-2_peak_545	1.27477	3.87658	2.08775	Chr3:740102-740562	740532	DM992_36080	WP_059493340.1|porin [Burkholderia cepacia]
Merged-Chr3-72457-2	Chr3:72260-72695	TTS (JP2270_GM007002_rna)	514	DM992_33230	1&2	BYSR-1_peak_591	1.36738	7.2065	4.58328	Chr3:72276-72695	72492	BYSR-2_peak_475	1.27263	3.97301	2.15452	Chr3:72260-72600	72419	DM992_33230	WP_088509613.1|IS3 family transposase, partial [Burkholderia ubonensis]
Merged-Chr3-718695-2	Chr3:718200-719531	TTS (JP2270_GM007550_rna)	1763	DM992_35975	1&2	BYSR-1_peak_649	1.34348	5.99997	3.69923	Chr3:718325-719531	719242	BYSR-2_peak_540	1.39461	6.58995	4.03181	Chr3:718200-718726	718442	DM992_35975	WP_175021397.1|3-oxoacyl-ACP synthase [Burkholderia lata]
Merged-Chr3-711141-2	Chr3:710449-712090	exon (JP2270_GM007546_rna, exon 1 of 1)	2440	DM992_35945	1&2	BYSR-1_peak_647	1.41014	8.06305	5.2118	Chr3:710566-712090	711386	BYSR-2_peak_536	1.49865	9.12055	5.7771	Chr3:710449-711460	710953	DM992_35945	WP_105393512.1|MULTISPECIES: H-NS histone family protein [Burkholderia cepacia complex]
Merged-Chr3-704704-2	Chr3:704231-705095	promoter	114	DM992_35915	1&2	BYSR-1_peak_645	1.43162	8.43042	5.47632	Chr3:704231-705095	704333	BYSR-2_peak_534	1.39195	6.24092	3.77896	Chr3:704419-705074	704976	DM992_35915	WP_175748875.1|hypothetical protein [Burkholderia pyrrocinia]
Merged-Chr3-66843-3	Chr3:66095-67638	promoter	-422	DM992_33210	1&2	BYSR-1_peak_589, BYSR-1_peak_588	-	-	-	-	-	BYSR-2_peak_472	1.44396	8.87518	5.60823	Chr3:66119-67616	66401	DM992_33210	WP_105393962.1|MULTISPECIES: cupin-like domain-containing protein [Burkholderia]
Merged-Chr3-638700-2	Chr3:637828-639558	promoter	315	DM992_35535	1&2	BYSR-1_peak_640	1.40762	6.93825	4.38591	Chr3:637828-639558	639228	BYSR-2_peak_528	1.23692	2.92584	1.43078	Chr3:638480-638934	638482	DM992_35535	WP_124670329.1|hypothetical protein [Burkholderia seminalis]
Merged-Chr3-629735-2	Chr3:629474-629906	promoter	-41	DM992_35500	1&2	BYSR-1_peak_638	1.32242	5.29966	3.1912	Chr3:629474-629906	629751	BYSR-2_peak_527	1.26176	3.42296	1.77317	Chr3:629656-629906	629768	DM992_35500	KVQ18844.1|hypothetical protein WK01_34890 [Burkholderia cepacia]
Merged-Chr3-619701-2	Chr3:619238-620191	promoter	-579	DM992_35465	1&2	BYSR-1_peak_637	1.45266	8.23654	5.33389	Chr3:619238-619828	619615	BYSR-2_peak_526	1.45991	7.56877	4.71401	Chr3:619548-620191	620038	DM992_35465	WP_059706252.1|MULTISPECIES: HU family DNA-binding protein [Burkholderia cepacia complex]
Merged-Chr3-58749-2	Chr3:58466-59047	promoter	311	DM992_33180	1&2	BYSR-1_peak_583	1.30121	5.23078	3.13976	Chr3:58497-59047	58775	BYSR-2_peak_467	1.36475	6.48521	3.95564	Chr3:58466-58987	58786	DM992_33180	WP_069228354.1|TetR/AcrR family transcriptional regulator [Burkholderia diffusa]
Merged-Chr3-57092-2	Chr3:56583-57386	promoter	284	DM992_33170	1&2	BYSR-1_peak_582	1.47083	8.9005	5.83297	Chr3:57101-57386	57268	BYSR-2_peak_466	1.21234	2.82092	1.35943	Chr3:56583-57298	56762	DM992_33170	WP_069228095.1|DMT family transporter [Burkholderia diffusa]
Merged-Chr3-553911-2	Chr3:553581-554190	promoter	-436	DM992_35165	1&2	BYSR-1_peak_635	1.43099	7.97874	5.14869	Chr3:553581-554190	554107	BYSR-2_peak_523	1.38408	6.22029	3.76312	Chr3:553719-554155	553907	DM992_35165	WP_072440343.1|MULTISPECIES: hypothetical protein [Burkholderia]
Merged-Chr3-551554-3	Chr3:550889-552382	promoter	15	DM992_35160	1&2	BYSR-1_peak_634	1.33353	5.537	3.36165	Chr3:550893-552382	551765	BYSR-2_peak_521, BYSR-2_peak_522	-	-	-	-	-	DM992_35160	WP_072440344.1|MULTISPECIES: replication initiation protein [Burkholderia]
Merged-Chr3-548297-2	Chr3:548000-548601	promoter	-293	DM992_35150	1&2	BYSR-1_peak_633	1.49458	9.74522	6.46845	Chr3:548022-548601	548422	BYSR-2_peak_520	1.5978	11.955	7.61926	Chr3:548000-548565	548179	DM992_35150	WP_006759638.1|MULTISPECIES: ParA family protein [Burkholderia]
Merged-Chr3-515818-2	Chr3:515577-516005	promoter	-220	DM992_34990	1&2	BYSR-1_peak_630	1.26884	3.87443	2.16759	Chr3:515706-515985	515940	BYSR-2_peak_515	1.35464	5.21494	3.0455	Chr3:515577-516005	515739	DM992_34990	WP_034182527.1|hypothetical protein [Burkholderia pyrrocinia]
Merged-Chr3-497297-2	Chr3:497033-497554	promoter	-541	DM992_34900	1&2	BYSR-1_peak_629	1.36889	7.4282	4.74425	Chr3:497033-497492	497214	BYSR-2_peak_514	1.40346	7.14146	4.41834	Chr3:497112-497554	497402	DM992_34900	WP_034182511.1|MULTISPECIES: DoxX family protein [Burkholderia]
Merged-Chr3-484876-2	Chr3:484563-485189	promoter	16	DM992_34840	1&2	BYSR-1_peak_628	1.34234	6.1325	3.7957	Chr3:484563-485189	485106	BYSR-2_peak_513	1.49524	9.95278	6.32951	Chr3:484606-485148	485026	DM992_34840	WP_175945255.1|hypothetical protein [Burkholderia pyrrocinia]
Merged-Chr3-47975-2	Chr3:47569-48303	promoter	488	DM992_33125	1&2	BYSR-1_peak_579	1.32888	5.63095	3.42906	Chr3:47954-48303	48206	BYSR-2_peak_465	1.38138	6.13916	3.70688	Chr3:47569-48076	47657	DM992_33125	WP_174431111.1|LysE family transporter [Burkholderia metallica]

Merged-Chr3-439543-2	Chr3:439174-439935	promoter	-500	DM992_34620	1&2	BYSR-1_peak_626	1.3946	8.10438	5.23938	Chr3:439174-439741	439405	BYSR-2_peak_511	1.42659	8.19308	5.13793	Chr3:439324-439935	439730	DM992_34620	WP_072440499.1|MULTISPECIES: cupin domain-containing protein [Burkholderia]
Merged-Chr3-438173-2	Chr3:437925-438376	promoter	-139	DM992_34615	1&2	BYSR-1_peak_625	1.30469	5.65363	3.44625	Chr3:437925-438376	438196	BYSR-2_peak_510	1.42797	8.73496	5.50722	Chr3:438017-438375	438180	DM992_34615	WP_034182467.1|MULTISPECIES: H-NS histone family protein [Burkholderia]
Merged-Chr3-436349-2	Chr3:436182-436497	promoter	-214	DM992_34600	1&2	BYSR-1_peak_624	1.40978	9.04246	5.94101	Chr3:436266-436497	436361	BYSR-2_peak_509	1.40046	7.77471	4.85327	Chr3:436182-436454	436374	DM992_34600	WP_096475769.1|carboxymuconolactone decarboxylase family protein [Burkholderia stabilis]
Merged-Chr3-435219-2	Chr3:434481-435837	promoter	-568	DM992_34590	1&2	BYSR-1_peak_623	1.36315	7.42061	4.73863	Chr3:434770-435791	435015	BYSR-2_peak_508	1.3901	7.38473	4.58618	Chr3:434481-435837	434814	DM992_34590	WP_065504481.1|efflux transporter outer membrane subunit [Burkholderia stabilis]
Merged-Chr3-426118-2	Chr3:425962-426292	promoter	153	DM992_34565	1&2	BYSR-1_peak_621	1.38341	7.80294	5.02037	Chr3:425962-426181	426082	BYSR-2_peak_506	1.28825	4.6229	2.61756	Chr3:426037-426292	426169	DM992_34565	WP_034182461.1|3-hydroxybutyrate dehydrogenase [Burkholderia pyrrocinia]
Merged-Chr3-424595-2	Chr3:423966-425191	promoter-TSS (JP2270_GM007271_rna)	581	DM992_34555	1&2	BYSR-1_peak_620	1.33384	6.6195	4.15369	Chr3:423966-425191	424309	BYSR-2_peak_505	1.29243	4.89509	2.81316	Chr3:424308-424917	424396	DM992_34555	WP_131949356.1|multidrug transporter [Burkholderia pyrrocinia]
Merged-Chr3-423602-2	Chr3:423363-423912	promoter	-161	DM992_34550	1&2	BYSR-1_peak_619	1.20479	2.96453	1.52057	Chr3:423363-423638	423626	BYSR-2_peak_504	1.35588	6.24741	3.78417	Chr3:423495-423912	423707	DM992_34550	WP_131949355.1|FAD-dependent oxidoreductase [Burkholderia pyrrocinia]
Merged-Chr3-415423-2	Chr3:415232-415650	promoter	-255	DM992_34515	1&2	BYSR-1_peak_616	1.31346	5.7307	3.50272	Chr3:415232-415494	415396	BYSR-2_peak_503	1.31127	4.94095	2.84704	Chr3:415318-415650	415403	DM992_34515	WP_175963413.1|helix-turn-helix transcriptional regulator [Burkholderia pyrrocinia]
Merged-Chr3-35893-2	Chr3:35752-36034	promoter	107	DM992_33080	1&2	BYSR-1_peak_577	1.42934	8.79949	5.75461	Chr3:35752-36022	35899	BYSR-2_peak_463	1.37244	6.20796	3.75464	Chr3:35767-36034	35916	DM992_33080	WP_080427666.1|DUF1697 domain-containing protein [Burkholderia ubonensis]
Merged-Chr3-34745-2	Chr3:34317-35030	promoter	-211	DM992_33070	1&2	BYSR-1_peak_576	1.42163	8.84947	5.79344	Chr3:34317-35030	34819	BYSR-2_peak_462	1.51834	11.0714	7.03933	Chr3:34611-35023	34793	DM992_33070	KVD88643.1|hypothetical protein WI90_19590 [Burkholderia ubonensis]
Merged-Chr3-301729-2	Chr3:301394-301981	promoter	-482	DM992_34025	1&2	BYSR-1_peak_612	1.51502	13.1122	9.10008	Chr3:301616-301981	301839	BYSR-2_peak_501	1.38471	7.13515	4.41392	Chr3:301394-301926	301822	DM992_34025	WP_034182376.1|MULTISPECIES: response regulator [Burkholderia]
Merged-Chr3-292174-2	Chr3:292016-292379	TTS (JP2270_GM007159_rna)	882	DM992_33995	1&2	BYSR-1_peak_611	1.26821	4.33233	2.49333	Chr3:292016-292264	292221	BYSR-2_peak_500	1.24819	3.58814	1.88574	Chr3:292040-292379	292374	DM992_33995	WP_175749173.1|aspartate/glutamate racemase family protein [Burkholderia pyrrocinia]
Merged-Chr3-273829-2	Chr3:273621-274052	promoter	-152	DM992_33920	1&2	BYSR-1_peak_610	1.33115	6.91288	4.36902	Chr3:273621-273891	273825	BYSR-2_peak_497	1.30397	5.34173	3.13561	Chr3:273753-274052	273786	DM992_33920	WP_174380406.1|DUF1059 domain-containing protein [Burkholderia pyrrocinia]
Merged-Chr3-258181-2	Chr3:258049-258327	promoter	-112	DM992_33855	1&2	BYSR-1_peak_608	1.24756	4.19782	2.39652	Chr3:258050-258301	258179	BYSR-2_peak_494	1.411	8.03934	5.03182	Chr3:258049-258327	258203	DM992_33855	WP_175963023.1|TauD/TfdA family dioxygenase [Burkholderia pyrrocinia]
Merged-Chr3-249578-2	Chr3:249374-249857	promoter	-403	DM992_33815	1&2	BYSR-1_peak_607	1.25136	3.68234	2.03109	Chr3:249385-249698	249655	BYSR-2_peak_493	1.30332	4.42928	2.47976	Chr3:249374-249857	249689	DM992_33815	WP_175749153.1|DUF2029 domain-containing protein [Burkholderia pyrrocinia]
Merged-Chr3-215631-2	Chr3:215447-215815	promoter	-478	DM992_33670	1&2	BYSR-1_peak_605	1.36647	7.29991	4.65104	Chr3:215447-215783	215629	BYSR-2_peak_490	1.42283	8.29083	5.20466	Chr3:215479-215815	215626	DM992_33670	WP_034182301.1|response regulator [Burkholderia pyrrocinia]
Merged-Chr3-187273-2	Chr3:187096-187433	promoter	-133	DM992_33565	1&2	BYSR-1_peak_604	1.2595	3.95802	2.22622	Chr3:187096-187426	187131	BYSR-2_peak_489	1.29661	4.21146	2.32335	Chr3:187139-187433	187188	DM992_33565	WP_034182290.1|transketolase [Burkholderia pyrrocinia]
Merged-Chr3-185373-2	Chr3:185039-185593	promoter	363	DM992_33550	1&2	BYSR-1_peak_603	1.34572	6.14511	3.80486	Chr3:185359-185593	185534	BYSR-2_peak_488	1.32451	4.96985	2.86768	Chr3:185039-185502	185363	DM992_33550	WP_034182584.1|MULTISPECIES: PRC-barrel domain-containing protein [Burkholderia cepacia complex]
Merged-Chr3-164738-2	Chr3:164346-165073	promoter	153	DM992_33470	1&2	BYSR-1_peak_602	1.44196	8.89918	5.8321	Chr3:164346-165010	164915	BYSR-2_peak_487	1.34395	5.364	3.15041	Chr3:164525-165073	164792	DM992_33470	PXX33849.1|two-component system CAI-1 autoinducer sensor kinase/phosphatase CqsS [Burkholderia pyrrocinia]
Merged-Chr3-163787-2	Chr3:163525-164082	promoter	-263	DM992_33465	1&2	BYSR-1_peak_601	1.40541	8.21127	5.31877	Chr3:163525-164012	163734	BYSR-2_peak_486	1.40969	7.22367	4.47488	Chr3:163531-164082	163848	DM992_33465	WP_131946417.1|response regulator [Burkholderia pyrrocinia]
Merged-Chr3-161268-2	Chr3:160852-161676	promoter	-75	DM992_33460	1&2	BYSR-1_peak_600	1.48107	11.0707	7.46918	Chr3:160852-161676	161221	BYSR-2_peak_484	1.53209	11.6889	7.44836	Chr3:161084-161460	161218	DM992_33460	WP_072439868.1|MULTISPECIES: quorum-sensing autoinducer CAI-1 synthase [Burkholderia]
Merged-Chr3-148813-2	Chr3:148426-149308	promoter	-698	DM992_33410	1&2	BYSR-1_peak_598	1.28277	4.69879	2.75581	Chr3:148439-149308	149051	BYSR-2_peak_482	1.35024	5.90666	3.54016	Chr3:148426-149080	149040	DM992_33410	WP_131946407.1|LuxR family transcriptional regulator [Burkholderia pyrrocinia]
Merged-Chr3-1459497-2	Chr3:1459190-1459722	promoter	-394	DM992_39160	1&2	BYSR-1_peak_755	1.25833	3.93378	2.20854	Chr3:1459190-1459722	1459498	BYSR-2_peak_639	1.3587	5.97079	3.58699	Chr3:1459406-1459671	1459461	DM992_39160	WP_174930201.1|type VI secretion system tip protein VgrG [Burkholderia lata]
Merged-Chr3-1458693-2	Chr3:1458481-1458890	promoter	205	DM992_39150	1&2	BYSR-1_peak_754	1.25293	3.79005	2.10803	Chr3:1458587-1458890	1458868	BYSR-2_peak_638	1.27915	4.07603	2.22716	Chr3:1458481-1458817	1458683	DM992_39150	KGC06895.1|bacterial regulatory s, gntR family protein [Burkholderia cepacia]
Merged-Chr3-1454489-2	Chr3:1453996-1454825	promoter	85	DM992_39125	1&2	BYSR-1_peak_752	1.26675	4.07337	2.30787	Chr3:1454564-1454825	1454573	BYSR-2_peak_636	1.29973	4.38741	2.45024	Chr3:1453996-1454571	1454014	DM992_39125	WP_059986262.1|3-hydroxyacyl-ACP dehydratase FabZ [Burkholderia stagnalis]
Merged-Chr3-145116-2	Chr3:144760-145475	promoter	-1020	DM992_33405	1&2	BYSR-1_peak_597	1.39781	7.95042	5.12703	Chr3:144760-145475	145134	BYSR-2_peak_481	1.23746	3.30348	1.69132	Chr3:144972-145257	145004	DM992_33405	WP_034182255.1|LuxR family transcriptional regulator [Burkholderia pyrrocinia]
Merged-Chr3-1448498-2	Chr3:1448256-1448956	promoter	382	DM992_39095	1&2	BYSR-1_peak_751	1.27823	4.4035	2.54539	Chr3:1448256-1448517	1448469	BYSR-2_peak_635	1.4904	10.0988	6.41299	Chr3:1448265-1448956	1448835	DM992_39095	WP_175748406.1|DinB family protein [Burkholderia pyrrocinia]
Merged-Chr3-1446566-2	Chr3:1446384-1446755	promoter	-139	DM992_39090	1&2	BYSR-1_peak_750	1.3106	5.15514	3.0866	Chr3:1446384-1446755	1446629	BYSR-2_peak_634	1.35623	5.78093	3.45106	Chr3:1446386-1446739	1446540	DM992_39090	WP_175962040.1|threonine ammonia-lyase, biosynthetic [Burkholderia pyrrocinia]
Merged-Chr3-1444593-2	Chr3:1444448-1444748	promoter	-374	DM992_39085	1&2	BYSR-1_peak_749	1.3042	5.50895	3.34092	Chr3:1444503-1444748	1444640	BYSR-2_peak_633	1.24471	3.55235	1.86038	Chr3:1444448-1444674	1444652	DM992_39085	WP_049036018.1|glycine C-acetyltransferase [Burkholderia seminalis]
Merged-Chr3-1433523-2	Chr3:1433245-1433825	promoter	409	DM992_39025	1&2	BYSR-1_peak_747	1.28551	4.57593	2.66739	Chr3:1433245-1433714	1433659	BYSR-2_peak_631	1.31549	4.8292	2.76664	Chr3:1433309-1433825	1433755	DM992_39025	WP_165580006.1|outer membrane beta-barrel protein [Burkholderia cepacia]
Merged-Chr3-1430312-2	Chr3:1430183-1430440	promoter	197	DM992_39015	1&2	BYSR-1_peak_746	1.38742	7.22737	4.59878	Chr3:1430183-1430429	1430351	BYSR-2_peak_630	1.39227	6.6026	4.04146	Chr3:1430196-1430440	1430355	DM992_39015	WP_096476038.1|fumarylacetoacetate hydrolase family protein [Burkholderia stabilis]
Merged-Chr3-1362826-2	Chr3:1362524-1363080	promoter	-506	DM992_38905	1&2	BYSR-1_peak_744	1.26819	4.48769	2.60507	Chr3:1362524-1363080	1363066	BYSR-2_peak_628	1.35216	5.97011	3.58642	Chr3:1362650-1363050	1362984	DM992_38905	WP_174962985.1|histidine phosphatase family protein [Burkholderia ubonensis]
Merged-Chr3-1355730-2	Chr3:1355358-1355982	promoter	64	DM992_38860	1&2	BYSR-1_peak_741	1.3393	6.44171	4.02205	Chr3:1355358-1355956	1355641	BYSR-2_peak_625	1.28328	4.38208	2.44608	Chr3:1355625-1355982	1355752	DM992_38860	WP_060173445.1|MULTISPECIES: NmrA family NAD(P)-binding protein [Burkholderia]
Merged-Chr3-1341287-2	Chr3:1341146-1341446	promoter	-122	DM992_38800	1&2	BYSR-1_peak_737	1.27563	4.24666	2.43262	Chr3:1341146-1341446	1341358	BYSR-2_peak_624	1.38465	6.36201	3.86571	Chr3:1341155-1341404	1341294	DM992_38800	WP_034184338.1|MULTISPECIES: MFS transporter [Burkholderia]

Merged-Chr3-1331358-2	Chr3:1331193-1331571	promoter	35	DM992_38760	1&2	BYSR-1_peak_736	1.49271	10.9787	7.40089	Chr3:1331222-1331571	1331398	BYSR-2_peak_623	1.4825	9.50977	6.03598	Chr3:1331193-1331447	1331303	DM992_38760	EDT37897.1|transcriptional regulator, LysR family [Burkholderia ambifaria MEX-5]
Merged-Chr3-1238483-2	Chr3:1238300-1238646	promoter	-344	DM992_38380	1&2	BYSR-1_peak_734	1.25355	4.14256	2.35864	Chr3:1238300-1238646	1238509	BYSR-2_peak_621	1.21846	2.883	1.40073	Chr3:1238366-1238621	1238561	DM992_38380	WP_175747863.1|TauD/TfdA family dioxygenase [Burkholderia pyrrocinia]
Merged-Chr3-1223421-2	Chr3:1222764-1224368	promoter	-1021	DM992_38310	1&2	BYSR-1_peak_730	1.38249	7.35338	4.69154	Chr3:1222764-1223575	1223366	BYSR-2_peak_617	1.39246	7.07203	4.37413	Chr3:1222977-1224368	1224324	DM992_38310	AIO27028.1|hypothetical protein DM41_7438 [Burkholderia cepacia ATCC 25416]
Merged-Chr3-1215062-2	Chr3:1214843-1215259	TTS (JP2270_GM008013_rna)	760	DM992_38265	1&2	BYSR-1_peak_729	1.36809	6.78777	4.27521	Chr3:1214843-1215259	1215033	BYSR-2_peak_616	1.33786	5.30449	3.10692	Chr3:1214949-1215199	1215034	DM992_38265	WP_175749411.1|depolymerase [Burkholderia pyrrocinia]
Merged-Chr3-1213715-2	Chr3:1213482-1213896	promoter	-602	DM992_38265	1&2	BYSR-1_peak_728	1.24177	3.8407	2.14281	Chr3:1213590-1213892	1213703	BYSR-2_peak_615	1.25302	3.78089	2.02107	Chr3:1213482-1213896	1213758	DM992_38265	WP_175749411.1|depolymerase [Burkholderia pyrrocinia]
Merged-Chr3-1197292-2	Chr3:1197005-1197649	promoter	185	DM992_38160	1&2	BYSR-1_peak_727	1.28244	4.67821	2.74053	Chr3:1197107-1197649	1197340	BYSR-2_peak_614	1.22173	3.01018	1.48816	Chr3:1197005-1197410	1197015	DM992_38160	WP_105393399.1|IS6 family transposase [Burkholderia cepacia]
Merged-Chr3-1196017-2	Chr3:1195597-1196725	promoter	-275	DM992_38155	1&2	BYSR-1_peak_726	1.30017	5.50733	3.33972	Chr3:1195600-1196725	1196621	BYSR-2_peak_613	1.428	8.48352	5.33719	Chr3:1195597-1196147	1195958	DM992_38155	WP_042585278.1|MULTISPECIES: hypothetical protein [Burkholderia]
Merged-Chr3-1168638-2	Chr3:1168109-1169265	promoter	-1110	DM992_38040	1&2	BYSR-1_peak_721	1.2465	4.03765	2.28447	Chr3:1168568-1169265	1168631	BYSR-2_peak_608	1.38958	7.28374	4.51772	Chr3:1168109-1168611	1168405	DM992_38040	WP_042585167.1|MULTISPECIES: Sec-independent protein translocase subunit TatA [Burkholderia]
Merged-Chr3-1165299-3	Chr3:1163916-1166046	promoter	-257	DM992_38030	1&2	BYSR-1_peak_720	1.35913	6.86424	4.3328	Chr3:1165055-1165886	1165113	BYSR-2_peak_606, BYSR-2_peak_607	-	-	-	-	-	DM992_38030	VWB68644.1|acyltransferase [Burkholderia pseudomultivorans]
Merged-Chr3-1159328-2	Chr3:1158811-1160067	promoter	123	DM992_38015	1&2	BYSR-1_peak_718	1.3118	5.4587	3.30624	Chr3:1159026-1160067	1159733	BYSR-2_peak_604	1.39453	7.01233	4.33156	Chr3:1158811-1159408	1159292	DM992_38015	WP_060149016.1|sugar transferase [Burkholderia ubonensis]
Merged-Chr3-1155909-2	Chr3:1155662-1156156	promoter	292	DM992_38000	1&2	BYSR-1_peak_717	1.40597	8.27367	5.36187	Chr3:1155662-1156156	1155837	BYSR-2_peak_602	1.28904	4.31358	2.39578	Chr3:1155758-1156060	1155865	DM992_38000	WP_059829888.1|glycosyltransferase family 2 protein [Burkholderia ubonensis]
Merged-Chr3-1151532-2	Chr3:1151242-1151826	promoter	-820	DM992_37990	1&2	BYSR-1_peak_715	1.33411	6.25184	3.8818	Chr3:1151242-1151826	1151708	BYSR-2_peak_601	1.29657	4.79345	2.74097	Chr3:1151254-1151809	1151474	DM992_37990	WP_059982095.1|hypothetical protein [Burkholderia ubonensis]
Merged-Chr3-1140226-2	Chr3:1139957-1140533	promoter	-12	DM992_37955	1&2	BYSR-1_peak_713	1.3496	6.60913	4.14539	Chr3:1140037-1140377	1140220	BYSR-2_peak_599	1.40497	7.48037	4.65229	Chr3:1139957-1140533	1140233	DM992_37955	WP_059750681.1|acyltransferase [Burkholderia ubonensis]
Merged-Chr3-1138769-3	Chr3:1137749-1139586	promoter	214	DM992_37950	1&2	BYSR-1_peak_712,BYSR-1_peak_711	-	-	-	-	-	BYSR-2_peak_598	1.42203	7.77433	4.85301	Chr3:1137749-1139462	1138712	DM992_37950	WP_042585174.1|MULTISPECIES: O-antigen ligase family protein [Burkholderia]
Merged-Chr3-1132261-2	Chr3:1131831-1132724	exon (JP2270_GM007946_rna, exon 1 of 1)	698	DM992_37935	1&2	BYSR-1_peak_709	1.32731	5.92465	3.64283	Chr3:1131831-1132724	1131878	BYSR-2_peak_595	1.34232	5.84711	3.50017	Chr3:1131926-1132565	1132456	DM992_37935	WP_042585278.1|MULTISPECIES: hypothetical protein [Burkholderia]
Merged-Chr3-1131252-2	Chr3:1131069-1131401	promoter	-344	DM992_37935	1&2	BYSR-1_peak_708	1.41816	9.28158	6.11922	Chr3:1131069-1131390	1131163	BYSR-2_peak_594	1.23233	3.30544	1.69249	Chr3:1131149-1131401	1131156	DM992_37935	WP_042585278.1|MULTISPECIES: hypothetical protein [Burkholderia]
Merged-Chr3-1130143-2	Chr3:1129796-1130640	TTS (JP2270_GM007945_rna)	1224	DM992_37925	1&2	BYSR-1_peak_707	1.30095	5.20988	3.12412	Chr3:1129796-1130250	1130099	BYSR-2_peak_593	1.3609	6.27375	3.80497	Chr3:1129886-1130640	1130189	DM992_37925	OUE39854.1|transposase [Burkholderia territorii]
Merged-Chr3-1039738-2	Chr3:1039217-1040361	promoter	-133	DM992_37500	1&2	BYSR-1_peak_705	1.51656	12.2953	8.42924	Chr3:1039217-1039958	1039569	BYSR-2_peak_592	1.28719	4.47844	2.51423	Chr3:1039418-1040361	1040024	DM992_37500	WP_010099209.1|porin [Burkholderia ubonensis]
Merged-Chr3-1034478-2	Chr3:1034355-1034626	promoter	-113	DM992_37470	1&2	BYSR-1_peak_702	1.3845	7.42904	4.74485	Chr3:1034358-1034575	1034453	BYSR-2_peak_589	1.34884	5.85549	3.50167	Chr3:1034355-1034626	1034495	DM992_37470	WP_151048243.1|RidA family protein [Burkholderia diffusa]
Merged-Chr3-1033080-2	Chr3:1032714-1033448	promoter	314	DM992_37460	1&2	BYSR-1_peak_701	1.37259	7.27011	4.62831	Chr3:1032764-1033448	1033082	BYSR-2_peak_587	1.48045	9.81621	6.24033	Chr3:1032714-1033394	1033047	DM992_37460	WP_043301681.1|HAD-IA family hydrolase [Burkholderia paludis]
Merged-Chr3-1030129-2	Chr3:1029849-1030520	promoter	433	DM992_37445	1&2	BYSR-1_peak_700	1.32893	5.81578	3.56408	Chr3:1029849-1030520	1029940	BYSR-2_peak_586	1.37143	6.32681	3.84081	Chr3:1029897-1030253	1029926	DM992_37445	WP_095412256.1|SAM-dependent methyltransferase [Burkholderia ubonensis]
Merged-Chr3-1027733-2	Chr3:1026878-1028834	TTS (JP2270_GM007844_rna)	2761	DM992_37445	1&2	BYSR-1_peak_699	1.44867	10.3351	6.90851	Chr3:1026956-1028834	1027166	BYSR-2_peak_585	1.31225	5.30082	3.10435	Chr3:1026878-1028266	1027030	DM992_37445	WP_095412256.1|SAM-dependent methyltransferase [Burkholderia ubonensis]
Merged-Chr3-1019096-2	Chr3:1018787-1019539	promoter	211	DM992_37395	1&2	BYSR-1_peak_696	1.3941	8.88652	5.82182	Chr3:1018826-1019539	1019087	BYSR-2_peak_584	1.3352	6.22926	3.76994	Chr3:1018787-1019233	1019045	DM992_37395	WP_034197579.1|MULTISPECIES: arylamine N-acetyltransferase [Burkholderia]
Merged-Chr3-1013115-2	Chr3:1012956-1013264	promoter	-168	DM992_37365	1&2	BYSR-1_peak_694	1.38598	6.75907	4.25644	Chr3:1012988-1013252	1013155	BYSR-2_peak_583	1.49381	8.84206	5.58367	Chr3:1012956-1013264	1013187	DM992_37365	WP_034184980.1|MULTISPECIES: hypothetical protein [Burkholderia]
Merged-Chr3-1008284-2	Chr3:1008031-1008687	promoter	247	DM992_37335	1&2	BYSR-1_peak_693	1.34091	6.37908	3.97475	Chr3:1008033-1008388	1008271	BYSR-2_peak_582	1.40408	7.52692	4.68378	Chr3:1008031-1008687	1008272	DM992_37335	WP_059481482.1|ABC transporter ATP-binding protein [Burkholderia pseudomultivorans]
Merged-Chr2-989182-2	Chr2:988843-989473	promoter	-383	DM992_22335	1&2	BYSR-1_peak_362	1.31258	5.40097	3.26372	Chr2:989047-989473	989348	BYSR-2_peak_290	1.50028	10.6378	6.76401	Chr2:988843-989367	989166	DM992_22335	WP_065501401.1|autotransporter-associated beta strand repeat-containing protein [Burkholderia stabilis]
Merged-Chr2-953365-2	Chr2:953172-953556	promoter	-49	DM992_22180	1&2	BYSR-1_peak_359	1.30558	5.94252	3.65635	Chr2:953223-953556	953405	BYSR-2_peak_289	1.44819	9.75168	6.19656	Chr2:953172-953510	953365	DM992_22180	WP_034180569.1|MULTISPECIES: helix-turn-helix transcriptional regulator [Burkholderia]
Merged-Chr2-952607-2	Chr2:952482-952731	promoter	-96	DM992_22175	1&2	BYSR-1_peak_358	1.40166	8.92578	5.84987	Chr2:952482-952714	952592	BYSR-2_peak_288	1.326	5.612	3.32894	Chr2:952502-952731	952612	DM992_22175	WP_047902668.1|MULTISPECIES: acyl-CoA thioesterase [Burkholderia]
Merged-Chr2-934537-2	Chr2:934219-934929	promoter	153	DM992_22080	1&2	BYSR-1_peak_356	1.31424	6.0596	3.74134	Chr2:934236-934929	934766	BYSR-2_peak_285	1.30446	5.33901	3.13343	Chr2:934219-934765	934434	DM992_22080	WP_014725167.1|MULTISPECIES: helix-turn-helix domain-containing protein [Burkholderia]
Merged-Chr2-931145-2	Chr2:930988-931380	promoter	-327	DM992_22065	1&2	BYSR-1_peak_355	1.26209	4.17406	2.37882	Chr2:930989-931380	931153	BYSR-2_peak_284	1.2551	3.62261	1.91013	Chr2:930988-931223	931139	DM992_22065	WP_014725166.1|MULTISPECIES: electron transfer flavoprotein-ubiquinone oxidoreductase [Burkholderia]
Merged-Chr2-91229-2	Chr2:91035-91480	promoter	-275	DM992_18055	1&2	BYSR-1_peak_279	1.32355	5.17852	3.10357	Chr2:91035-91333	91091	BYSR-2_peak_221	1.37522	5.75476	3.43044	Chr2:91068-91480	91362	DM992_18055	WP_034179516.1|MULTISPECIES: type VI secretion system lipoprotein TssJ [Burkholderia]
Merged-Chr2-8872-2	Chr2:8529-9237	promoter	-250	DM992_17720	1&2	BYSR-1_peak_276	1.42408	8.79782	5.75359	Chr2:8579-9144	8760	BYSR-2_peak_214	1.40095	6.96541	4.29957	Chr2:8529-9237	8814	DM992_17720	WP_083534041.1|MULTISPECIES: response regulator transcription factor [Burkholderia]
Merged-Chr2-875513-2	Chr2:875319-875701	promoter	-75	DM992_21800	1&2	BYSR-1_peak_346	1.32696	6.04778	3.73216	Chr2:875342-875692	875552	BYSR-2_peak_280	1.48511	10.2543	6.51936	Chr2:875319-875701	875525	DM992_21800	EPZ89959.1|autoinducer-binding domain protein [Burkholderia cenocepacia K56-2Valvano]

Merged-Chr2-855646-2	Chr2:855467-855805	promoter	73	DM992_21705	1&2	BYSR-1_peak_345	1.35167	6.40458	3.99358	Chr2:855467-855805	855667	BYSR-2_peak_278	1.46632	8.84068	5.58263	Chr2:855533-855782	855678	DM992_21705	WP_105391120.1|MULTISPECIES: winged helix-turn-helix transcriptional regulator [Burkholderia]
Merged-Chr2-849652-2	Chr2:849422-849820	promoter	-242	DM992_21670	1&2	BYSR-1_peak_343	1.36873	6.82439	4.30265	Chr2:849571-849796	849675	BYSR-2_peak_276	1.42874	7.89555	4.93459	Chr2:849422-849820	849714	DM992_21670	WP_034180982.1|hypothetical protein [Burkholderia pyrrocinia]
Merged-Chr2-741289-2	Chr2:740911-741560	promoter	208	DM992_21085	1&2	BYSR-1_peak_333	1.21198	3.05317	1.58232	Chr2:740911-741495	741039	BYSR-2_peak_271	1.23342	3.08436	1.5408	Chr2:741192-741560	741193	DM992_21085	WP_124676322.1|hypothetical protein [Burkholderia cenocepacia]
Merged-Chr2-734946-3	Chr2:733792-735804	promoter	-388	DM992_21045	1&2	BYSR-1_peak_331	1.41788	8.83594	5.78283	Chr2:733792-735804	734073	BYSR-2_peak_269, BYSR-2_peak_270	-	-	-	-	-	DM992_21045	VWC09893.1|hypothetical protein BSE24067_05298 [Burkholderia seminalis]
Merged-Chr2-699679-2	Chr2:699128-700048	promoter	-19	DM992_20875	1&2	BYSR-1_peak_330	1.41888	7.25289	4.61502	Chr2:699523-700019	699939	BYSR-2_peak_267	1.27642	3.70373	1.96604	Chr2:699128-700048	699213	DM992_20875	WP_096473943.1|porin [Burkholderia stabilis]
Merged-Chr2-697388-2	Chr2:697219-697555	promoter	-7	DM992_20865	1&2	BYSR-1_peak_329	1.44329	8.8566	5.79885	Chr2:697242-697555	697426	BYSR-2_peak_266	1.29115	3.9965	2.17219	Chr2:697219-697539	697376	DM992_20865	WP_174421126.1|MULTISPECIES: DUF4148 domain-containing protein [Burkholderia cepacia complex]
Merged-Chr2-691952-2	Chr2:691690-692150	promoter	-47	DM992_20835	1&2	BYSR-1_peak_328	1.30335	5.04814	3.00923	Chr2:691690-692149	692022	BYSR-2_peak_263	1.32687	5.03595	2.91442	Chr2:691821-692150	692048	DM992_20835	WP_006478802.1|MULTISPECIES: DUF4148 domain-containing protein [Burkholderia]
Merged-Chr2-677730-2	Chr2:677338-678372	promoter	264	DM992_20770	1&2	BYSR-1_peak_327	1.38672	6.69066	4.20424	Chr2:677368-678372	678280	BYSR-2_peak_262	1.3944	6.1601	3.72184	Chr2:677338-677843	677524	DM992_20770	WP_006478812.1|hypothetical protein [Burkholderia cenocepacia]
Merged-Chr2-660766-4	Chr2:659605-661663	TTS (JP2270_GM004487_rna)	997	DM992_20695	1&2	BYSR-1_peak_326	1.27496	4.20746	2.40369	Chr2:659845-661474	660916	BYSR-2_peak_259, BYSR-2_peak_260, BYSR-2_peak_261	-	-	-	-	-	DM992_20695	WP_048249937.1|DMT family transporter [Burkholderia cepacia]
Merged-Chr2-651307-2	Chr2:650623-652182	promoter	98	DM992_20660	1&2	BYSR-1_peak_325	1.409	7.44295	4.75548	Chr2:651068-652182	652055	BYSR-2_peak_258	1.40583	6.82612	4.19957	Chr2:650623-651356	651171	DM992_20660	WP_069238327.1|aspartate--tRNA(Asn) ligase [Burkholderia ubonensis]
Merged-Chr2-63145-2	Chr2:62690-63782	TTS (JP2270_GM003940_rna)	1116	DM992_17970	1&2	BYSR-1_peak_277	1.5453	12.6004	8.67912	Chr2:62690-63409	62923	BYSR-2_peak_219	1.30142	5.06181	2.93472	Chr2:62702-63782	63527	DM992_17970	WP_174916884.1|IS3 family transposase [Burkholderia lata]
Merged-Chr2-620667-2	Chr2:620485-620827	promoter	-46	DM992_20495	1&2	BYSR-1_peak_321	1.36675	6.15705	3.8141	Chr2:620485-620777	620631	BYSR-2_peak_253	1.38107	6.00491	3.6096	Chr2:620581-620827	620746	DM992_20495	WP_105393352.1|ribosome-associated translation inhibitor RaiA [Burkholderia cepacia]
Merged-Chr2-609544-2	Chr2:609320-609822	promoter	366	DM992_20445	1&2	BYSR-1_peak_320	1.32799	5.50994	3.34151	Chr2:609463-609822	609593	BYSR-2_peak_251	1.27017	3.59316	1.88903	Chr2:609320-609571	609536	DM992_20445	WP_017335015.1|MULTISPECIES: DUF1003 domain-containing protein [Burkholderia]
Merged-Chr2-607966-2	Chr2:607609-608275	promoter	338	DM992_20435	1&2	BYSR-1_peak_319	1.34703	5.57617	3.39064	Chr2:607609-608275	607945	BYSR-2_peak_250	1.33797	4.76194	2.71696	Chr2:607861-608120	607909	DM992_20435	WP_105393979.1|amino acid ABC transporter substrate-binding protein [Burkholderia cepacia]
Merged-Chr2-606473-2	Chr2:606148-606849	promoter	282	DM992_20430	1&2	BYSR-1_peak_318	1.44167	8.17464	5.29224	Chr2:606148-606849	606246	BYSR-2_peak_249	1.47622	8.53394	5.36935	Chr2:606155-606743	606352	DM992_20430	WP_105393594.1|response regulator transcription factor [Burkholderia cepacia]
Merged-Chr2-599821-2	Chr2:599510-600037	promoter	1	DM992_20385	1&2	BYSR-1_peak_317	1.28909	4.38285	2.5307	Chr2:599510-600037	599979	BYSR-2_peak_248	1.39251	6.58603	4.02863	Chr2:599714-600026	599834	DM992_20385	WP_072436452.1|MULTISPECIES: hypothetical protein [Burkholderia]
Merged-Chr2-513065-2	Chr2:512781-513390	promoter	-189	DM992_19955	1&2	BYSR-1_peak_314	1.40212	7.47611	4.78054	Chr2:512853-513239	513142	BYSR-2_peak_245	1.38439	6.31311	3.83263	Chr2:512781-513390	513260	DM992_19955	WP_131946186.1|MCP four helix bundle domain-containing protein [Burkholderia pyrrocinia]
Merged-Chr2-470536-2	Chr2:470303-470734	promoter	-413	DM992_19765	1&2	BYSR-1_peak_310	1.25584	3.73728	2.069	Chr2:470390-470720	470652	BYSR-2_peak_243	1.49277	9.57063	6.07774	Chr2:470303-470734	470608	DM992_19765	WP_175958868.1|hypothetical protein [Burkholderia pyrrocinia]
Merged-Chr2-451517-2	Chr2:451232-451938	promoter	157	DM992_26165	1&2	BYSR-1_peak_309	1.36351	6.27806	3.90163	Chr2:451232-451651	451388	BYSR-2_peak_242	1.30345	4.90344	2.81907	Chr2:451247-451938	451767	DM992_26165	WP_105823359.1|epoxide hydrolase [Burkholderia multivorans]
Merged-Chr2-449757-2	Chr2:449431-450090	promoter	264	DM992_19680	1&2	BYSR-1_peak_308	1.47695	9.15005	6.02368	Chr2:449466-450090	450036	BYSR-2_peak_241	1.33376	5.16482	3.00743	Chr2:449431-450042	449504	DM992_19680	WP_105823359.1|epoxide hydrolase [Burkholderia multivorans]
Merged-Chr2-448757-2	Chr2:448565-448963	TTS (JP2270_GM004282_rna)	1002	DM992_19670	1&2	BYSR-1_peak_307	1.32106	5.71855	3.49361	Chr2:448565-448963	448898	BYSR-2_peak_240	1.34693	5.574	3.30019	Chr2:448618-448884	448736	DM992_19670	WP_031400700.1|cupin domain-containing protein [Burkholderia paludis]
Merged-Chr2-448002-2	Chr2:447614-448328	promoter	209	DM992_19670	1&2	BYSR-1_peak_306	1.48628	10.0123	6.66942	Chr2:447614-448196	448066	BYSR-2_peak_239	1.29192	4.12211	2.26085	Chr2:447872-448328	447924	DM992_19670	WP_031400700.1|cupin domain-containing protein [Burkholderia paludis]
Merged-Chr2-444433-2	Chr2:443920-444875	promoter	-295	DM992_19650	1&2	BYSR-1_peak_305	1.32718	5.57407	3.38899	Chr2:443920-444873	444678	BYSR-2_peak_237	1.40164	7.02784	4.34253	Chr2:444066-444875	444341	DM992_19650	WP_096476142.1|hypothetical protein [Burkholderia stabilis]
Merged-Chr2-443482-2	Chr2:443312-443618	TTS (JP2270_GM004275_rna)	628	DM992_19630	1&2	BYSR-1_peak_304	1.33083	5.35681	3.23115	Chr2:443382-443616	443445	BYSR-2_peak_236	1.31243	4.40896	2.4652	Chr2:443312-443618	443474	DM992_19630	KKI39748.1|hypothetical protein VI03_07025 [Burkholderia vietnamiensis]
Merged-Chr2-3226023-2	Chr2:3225866-3226183	promoter	-293	DM992_32795	1&2	BYSR-1_peak_573	1.32292	5.95921	3.66883	Chr2:3225889-3226183	3226130	BYSR-2_peak_461	1.2651	3.88517	2.09386	Chr2:3225866-3226157	3226141	DM992_32795	WP_105391923.1|phosphoenolpyruvate carboxykinase (GTP) [Burkholderia cepacia]
Merged-Chr2-3220237-2	Chr2:3219800-3220696	promoter	-197	DM992_32755	1&2	BYSR-1_peak_572	1.23741	3.82529	2.13164	Chr2:3219800-3220696	3219848	BYSR-2_peak_460	1.21492	2.86513	1.39036	Chr2:3219954-3220500	3219957	DM992_32755	WP_105391924.1|nuclear transport factor 2 family protein [Burkholderia cepacia]
Merged-Chr2-3218883-2	Chr2:3218745-3219023	promoter	-33	DM992_32745	1&2	BYSR-1_peak_571	1.39035	8.15599	5.27754	Chr2:3218799-3219023	3218901	BYSR-2_peak_459	1.31129	5.23231	3.05648	Chr2:3218745-3218968	3218825	DM992_32745	PXX40957.1|hypothetical protein NA66_1001567 [Burkholderia pyrrocinia]
Merged-Chr2-3130812-2	Chr2:3130524-3131029	promoter	-564	DM992_32285	1&2	BYSR-1_peak_567	1.27935	4.56912	2.66222	Chr2:3130524-3131029	3130966	BYSR-2_peak_456	1.4121	7.83134	4.88962	Chr2:3130689-3131006	3130877	DM992_32285	WP_059854052.1|MULTISPECIES: hypothetical protein [Burkholderia]
Merged-Chr2-3129612-3	Chr2:3129221-3129958	promoter	-564	DM992_32280	1&2	BYSR-1_peak_566	1.32431	5.86497	3.60078	Chr2:3129432-3129849	3129806	BYSR-2_peak_454, BYSR-2_peak_455	-	-	-	-	-	DM992_32280	WP_060061626.1|hypothetical protein [Burkholderia cepacia]
Merged-Chr2-3128099-2	Chr2:3127535-3128679	promoter	-712	DM992_32275	1&2	BYSR-1_peak_565	1.28522	4.87458	2.88312	Chr2:3127535-3128609	3128531	BYSR-2_peak_453	1.34714	5.99834	3.60602	Chr2:3127575-3128679	3127776	DM992_32275	WP_060195515.1|UbiA family prenyltransferase [Burkholderia cepacia]
Merged-Chr2-3118306-2	Chr2:3118100-3118488	promoter	-336	DM992_32265	1&2	BYSR-1_peak_564	1.36485	7.56802	4.84805	Chr2:3118100-3118484	3118273	BYSR-2_peak_452	1.39	7.77513	4.85352	Chr2:3118152-3118488	3118305	DM992_32265	WP_122169757.1|hemagglutinin repeat-containing protein [Burkholderia stabilis]
Merged-Chr2-3098662-2	Chr2:3098313-3098923	promoter	-364	DM992_32180	1&2	BYSR-1_peak_563	1.29552	5.56942	3.38568	Chr2:3098313-3098914	3098451	BYSR-2_peak_451	1.49483	10.5091	6.67545	Chr2:3098499-3098923	3098698	DM992_32180	WP_072438718.1|MULTISPECIES: MFS transporter [Burkholderia]
Merged-Chr2-3097052-2	Chr2:3096742-3097302	promoter	-302	DM992_32165	1&2	BYSR-1_peak_562	1.32953	6.26495	3.89121	Chr2:3096742-3097278	3097094	BYSR-2_peak_450	1.41589	8.12547	5.0945	Chr2:3096889-3097302	3097111	DM992_32165	WP_126365707.1|DUF2837 family protein [Burkholderia cenocepacia]

Merged-Chr2-3095679-2	Chr2:3095438-3095909	promoter	-474	DM992_32155	1&2	BYSR-1_peak_561	1.4319	9.946	6.62122	Chr2:3095484-3095909	3095693	BYSR-2_peak_448	1.50992	11.8124	7.53239	Chr2:3095438-3095886	3095672	DM992_32155	WP_105394044.1|IS30 family transposase [Burkholderia cepacia]
Merged-Chr2-3092336-2	Chr2:3091821-3092747	promoter	2	DM992_32145	1&2	BYSR-1_peak_559	1.38865	7.65031	4.90746	Chr2:3091821-3092709	3091997	BYSR-2_peak_446	1.30174	4.69047	2.66684	Chr2:3092069-3092747	3092376	DM992_32145	AIO37084.1|LGFP repeat family protein [Burkholderia cenocepacia]
Merged-Chr2-2998963-2	Chr2:2998783-2999241	promoter	-125	DM992_31725	1&2	BYSR-1_peak_551	1.47011	10.7477	7.2244	Chr2:2998783-2999009	2998948	BYSR-2_peak_442	1.39103	7.14606	4.42137	Chr2:2998820-2999241	2998942	DM992_31725	WP_072438967.1|MULTISPECIES: oxalate decarboxylase family bicupin [Burkholderia]
Merged-Chr2-2997041-2	Chr2:2996807-2997241	promoter	-79	DM992_31715	1&2	BYSR-1_peak_550	1.31492	5.28612	3.18105	Chr2:2996807-2997241	2996876	BYSR-2_peak_441	1.31141	4.57485	2.58255	Chr2:2996904-2997214	2997072	DM992_31715	WP_111020062.1|MULTISPECIES: helix-turn-helix transcriptional regulator [Burkholderia]
Merged-Chr2-2965658-2	Chr2:2965478-2965835	exon (JP2270_GM006677_rna, exon 1 of 1)	1179	DM992_31560	1&2	BYSR-1_peak_549	1.38221	7.62042	4.88443	Chr2:2965478-2965835	2965612	BYSR-2_peak_439	1.43407	8.05787	5.04577	Chr2:2965518-2965804	2965672	DM992_31560	WP_034180303.1|type VI secretion system membrane subunit TssM [Burkholderia pyrrocinia]
Merged-Chr2-2960541-2	Chr2:2960228-2960952	promoter	108	DM992_31545	1&2	BYSR-1_peak_547	1.22693	3.26831	1.73477	Chr2:2960228-2960634	2960326	BYSR-2_peak_438	1.3135	5.00501	2.89248	Chr2:2960353-2960952	2960847	DM992_31545	WP_096475835.1|DUF2235 domain-containing protein [Burkholderia stabilis]
Merged-Chr2-2949863-2	Chr2:2949724-2950035	promoter	-35	DM992_31490	1&2	BYSR-1_peak_543	1.26186	3.85622	2.15429	Chr2:2949724-2949971	2949931	BYSR-2_peak_436	1.39916	6.5356	3.99281	Chr2:2949724-2950035	2949833	DM992_31490	WP_034180314.1|MULTISPECIES: type VI secretion system contractile sheath small subunit [Burkholderia]
Merged-Chr2-2947117-2	Chr2:2946849-2947358	promoter	-92	DM992_31485	1&2	BYSR-1_peak_542	1.347	6.61243	4.14805	Chr2:2946906-2947358	2946954	BYSR-2_peak_435	1.48995	9.99282	6.35501	Chr2:2946849-2947358	2947040	DM992_31485	WP_034180315.1|type VI secretion system ATPase TssH [Burkholderia pyrrocinia]
Merged-Chr2-2940380-2	Chr2:2940094-2940654	promoter	252	DM992_31450	1&2	BYSR-1_peak_541	1.37032	7.24781	4.61402	Chr2:2940094-2940545	2940375	BYSR-2_peak_434	1.41881	7.56975	4.71454	Chr2:2940228-2940654	2940332	DM992_31450	KFL53673.1|hypothetical protein JM78_09055 [Burkholderia pyrrocinia]
Merged-Chr2-2939638-2	Chr2:2939478-2939815	promoter	-476	DM992_31450	1&2	BYSR-1_peak_540	1.23756	3.26554	1.73276	Chr2:2939478-2939749	2939729	BYSR-2_peak_433	1.47562	8.72771	5.50218	Chr2:2939512-2939815	2939641	DM992_31450	KFL53673.1|hypothetical protein JM78_09055 [Burkholderia pyrrocinia]
Merged-Chr2-2924101-2	Chr2:2923781-2924369	promoter	261	DM992_31375	1&2	BYSR-1_peak_539	1.42301	8.46933	5.50597	Chr2:2923781-2924336	2924163	BYSR-2_peak_432	1.32381	4.97167	2.86912	Chr2:2923918-2924369	2924098	DM992_31375	WP_072443292.1|MULTISPECIES: heavy metal-responsive transcriptional regulator [Burkholderia]
Merged-Chr2-2788275-2	Chr2:2788053-2788482	promoter	-107	DM992_30760	1&2	BYSR-1_peak_534	1.31464	4.88971	2.8943	Chr2:2788164-2788482	2788322	BYSR-2_peak_427	1.39152	6.23163	3.77181	Chr2:2788053-2788403	2788246	DM992_30760	WP_034179974.1|acyltransferase [Burkholderia pyrrocinia]
Merged-Chr2-270517-2	Chr2:270284-270804	promoter	-92	DM992_18885	1&2	BYSR-1_peak_296	1.32826	5.56624	3.38349	Chr2:270284-270509	270443	BYSR-2_peak_234	1.29493	4.27843	2.37212	Chr2:270471-270804	270635	DM992_18885	WP_105843172.1|recombinase family protein [Burkholderia multivorans]
Merged-Chr2-2702688-2	Chr2:2702566-2702813	promoter	65	DM992_30355	1&2	BYSR-1_peak_532	1.29373	4.15661	2.36855	Chr2:2702577-2702813	2702665	BYSR-2_peak_422	1.39702	6.17082	3.72684	Chr2:2702566-2702797	2702698	DM992_30355	ABB12298.1|protein of unknown function DUF1311 [Burkholderia lata]
Merged-Chr2-268951-2	Chr2:268165-269737	promoter	-616	DM992_18875	1&2	BYSR-1_peak_295	1.36282	6.94434	4.39025	Chr2:268767-269135	268955	BYSR-2_peak_233	1.44191	8.36669	5.25562	Chr2:268165-269737	268246	DM992_18875	WP_154641254.1|hypothetical protein [Burkholderia cenocepacia]
Merged-Chr2-2683134-2	Chr2:2682766-2683614	promoter	-57	DM992_30265	1&2	BYSR-1_peak_528	1.26816	4.13885	2.35577	Chr2:2682832-2683327	2683160	BYSR-2_peak_420	1.35682	5.85139	3.50167	Chr2:2682766-2683614	2683179	DM992_30265	WP_105391452.1|MULTISPECIES: hypothetical protein [Burkholderia cepacia complex]
Merged-Chr2-2679999-2	Chr2:2679694-2680429	promoter	-517	DM992_30250	1&2	BYSR-1_peak_526	1.38044	6.77237	4.26601	Chr2:2679712-2680162	2680084	BYSR-2_peak_419	1.41285	6.77328	4.16464	Chr2:2679694-2680429	2679951	DM992_30250	KUY72921.1|transposase [Burkholderia cepacia]
Merged-Chr2-2607522-2	Chr2:2607341-2607759	promoter	-137	DM992_29900	1&2	BYSR-1_peak_523	1.28343	4.35877	2.5128	Chr2:2607341-2607759	2607689	BYSR-2_peak_417	1.32415	4.66649	2.64951	Chr2:2607366-2607623	2607566	DM992_29900	GAU05033.1|undecaprenyl-phosphate glucose phosphotransferase [Burkholderia stabilis]
Merged-Chr2-2604453-2	Chr2:2604233-2604656	promoter	-402	DM992_29890	1&2	BYSR-1_peak_522	1.57486	14.5328	10.2256	Chr2:2604233-2604656	2604355	BYSR-2_peak_416	1.21092	2.87014	1.39407	Chr2:2604278-2604645	2604484	DM992_29890	WP_105392306.1|MULTISPECIES: transposase [Burkholderia cepacia complex]
Merged-Chr2-2571104-2	Chr2:2570974-2571225	promoter	-147	DM992_29725	1&2	BYSR-1_peak_520	1.30391	5.19342	3.11195	Chr2:2570974-2571217	2571170	BYSR-2_peak_414	1.2696	3.84039	2.0616	Chr2:2571000-2571225	2571224	DM992_29725	WP_175958612.1|hybrid sensor histidine kinase/response regulator [Burkholderia pyrrocinia]
Merged-Chr2-2546306-2	Chr2:2546039-2546505	TTS (JP2270_GM006288_rna)	1008	DM992_29620	1&2	BYSR-1_peak_516	1.27125	4.22848	2.41918	Chr2:2546039-2546463	2546439	BYSR-2_peak_413	1.39902	7.01001	4.32974	Chr2:2546219-2546505	2546417	DM992_29620	BBA39689.1|hypothetical protein BCCH1_21120 [Burkholderia contaminans]
Merged-Chr2-2543614-2	Chr2:2543347-2543904	promoter	128	DM992_29600	1&2	BYSR-1_peak_515	1.39086	7.99617	5.16122	Chr2:2543351-2543856	2543525	BYSR-2_peak_411	1.50129	10.5069	6.67391	Chr2:2543347-2543904	2543632	DM992_29600	WP_157915376.1|hypothetical protein [[Pseudomonas] mesoacidophila]
Merged-Chr2-2540407-2	Chr2:2540169-2540619	promoter	-211	DM992_29585	1&2	BYSR-1_peak_514	1.24561	3.82022	2.12791	Chr2:2540263-2540619	2540535	BYSR-2_peak_409	1.26953	4.08255	2.23185	Chr2:2540169-2540577	2540246	DM992_29585	WP_084908072.1|hypothetical protein [[Pseudomonas] mesoacidophila]
Merged-Chr2-2535564-2	Chr2:2535285-2535876	promoter	-265	DM992_29565	1&2	BYSR-1_peak_511	1.49782	11.5187	7.81404	Chr2:2535285-2535876	2535588	BYSR-2_peak_408	1.4394	8.22175	5.15746	Chr2:2535437-2535658	2535553	DM992_29565	WP_131948043.1|type VI secretion system baseplate subunit TssK [Burkholderia pyrrocinia]
Merged-Chr2-2508335-2	Chr2:2507704-2508919	promoter	-632	DM992_29440	1&2	BYSR-1_peak_508	1.29236	5.59542	3.4049	Chr2:2507704-2508908	2508099	BYSR-2_peak_407	1.50083	11.071	7.03933	Chr2:2507812-2508919	2508644	DM992_29440	WP_175960861.1|TIGR02594 family protein [Burkholderia pyrrocinia]
Merged-Chr2-2503208-2	Chr2:2502842-2503624	promoter	-62	DM992_29415	1&2	BYSR-1_peak_505	1.27763	4.94867	2.93552	Chr2:2502842-2503624	2503482	BYSR-2_peak_406	1.20307	2.76375	1.32216	Chr2:2502863-2503506	2503398	DM992_29415	WP_065503752.1|hypothetical protein [Burkholderia stabilis]
Merged-Chr2-2499336-2	Chr2:2498729-2499881	promoter	405	DM992_29400	1&2	BYSR-1_peak_503	1.42923	9.37873	6.19153	Chr2:2498729-2499823	2499341	BYSR-2_peak_404	1.41009	7.82529	4.88603	Chr2:2498912-2499881	2499319	DM992_29400	WP_065502675.1|PAAR domain-containing protein [Burkholderia stabilis]
Merged-Chr2-2497991-2	Chr2:2497479-2498468	promoter	186	DM992_29395	1&2	BYSR-1_peak_502	1.41369	9.1413	6.0161	Chr2:2497479-2498287	2497930	BYSR-2_peak_403	1.37547	7.12326	4.40958	Chr2:2497733-2498468	2498029	DM992_29395	WP_083214469.1|hypothetical protein [Burkholderia stabilis]
Merged-Chr2-2477224-2	Chr2:2476885-2477429	promoter	-22	DM992_29300	1&2	BYSR-1_peak_501	1.30105	5.84689	3.58726	Chr2:2477155-2477428	2477364	BYSR-2_peak_402	1.3459	6.27611	3.80526	Chr2:2476885-2477429	2477101	DM992_29300	WP_063778223.1|MULTISPECIES: K(+)-transporting ATPase subunit F [Burkholderia]
Merged-Chr2-2420439-2	Chr2:2420289-2420564	promoter	-85	DM992_29025	1&2	BYSR-1_peak_497	1.29717	5.01972	2.98846	Chr2:2420289-2420564	2420342	BYSR-2_peak_401	1.30975	4.80103	2.74639	Chr2:2420340-2420564	2420443	DM992_29025	WP_174383081.1|MULTISPECIES: fucose-binding lectin protein [Burkholderia cepacia complex]
Merged-Chr2-2405988-2	Chr2:2405691-2406171	promoter	-275	DM992_28970	1&2	BYSR-1_peak_495	1.19004	3.03621	1.57252	Chr2:2405931-2406171	2405932	BYSR-2_peak_400	1.33234	6.30696	3.82798	Chr2:2405691-2406160	2405970	DM992_28970	WP_175900972.1|IS256 family transposase [Burkholderia cepacia]
Merged-Chr2-2405108-2	Chr2:2404475-2405454	TTS (JP2270_GM006154_rna)	1015	DM992_28960	1&2	BYSR-1_peak_494	1.33598	6.59381	4.13413	Chr2:2404475-2405371	2405303	BYSR-2_peak_399	1.38142	7.30329	4.52887	Chr2:2405134-2405454	2405321	DM992_28960	AJX12699.1|hypothetical protein BW23_5081 [Burkholderia ubonensis MSMB22]
Merged-Chr2-2402511-3	Chr2:2401336-2403828	promoter	-625	DM992_28955	1&2	BYSR-1_peak_493, BYSR-1_peak_492	-	-	-	-	-	BYSR-2_peak_398	1.28894	5.07147	2.94263	Chr2:2401336-2403785	2401654	DM992_28955	WP_174973906.1|hypothetical protein [Burkholderia contaminans]

Merged-Chr2-2399697-2	Chr2:2399445-2399861	exon (JP2270_GM006152_rna, exon 1 of 1)	-3554	DM992_28955	1&2	BYSR-1_peak_491	1.21458	3.26114	1.72959	Chr2:2399632-2399852	2399736	BYSR-2_peak_397	1.34866	6.19972	3.75137	Chr2:2399445-2399861	2399750	DM992_28955	WP_174973906.1|hypothetical protein [Burkholderia contaminans]
Merged-Chr2-239405-2	Chr2:239184-239609	promoter	92	DM992_18755	1&2	BYSR-1_peak_294	1.34223	5.82719	3.57243	Chr2:239184-239590	239401	BYSR-2_peak_232	1.28108	3.98352	2.16248	Chr2:239237-239609	239501	DM992_18755	WP_006755380.1|MULTISPECIES: Lrp/AsnC family transcriptional regulator [Burkholderia]
Merged-Chr2-2392122-2	Chr2:2391779-2392478	promoter	-192	DM992_28945	1&2	BYSR-1_peak_488	1.4337	10.2872	6.87486	Chr2:2391780-2392453	2392315	BYSR-2_peak_396	1.40388	8.14475	5.10789	Chr2:2391779-2392478	2392316	DM992_28945	WP_105389695.1|histone acetyltransferase [Burkholderia cepacia]
Merged-Chr2-2230969-2	Chr2:2230624-2231321	promoter	-115	DM992_28170	1&2	BYSR-1_peak_480	1.39148	9.26137	6.10649	Chr2:2230624-2231321	2230792	BYSR-2_peak_394	1.38686	7.77315	4.85256	Chr2:2230736-2231197	2230856	DM992_28170	WP_175963663.1|hypothetical protein [Burkholderia pyrrocinia]
Merged-Chr2-2229972-2	Chr2:2229623-2230307	promoter	127	DM992_28165	1&2	BYSR-1_peak_479	1.486	13.2467	9.20507	Chr2:2229623-2230307	2230120	BYSR-2_peak_393	1.23335	3.67931	1.94868	Chr2:2229691-2230268	2230021	DM992_28165	WP_072438548.1|MULTISPECIES: glycosyl transferase family protein [Burkholderia]
Merged-Chr2-2225394-2	Chr2:2225144-2225641	promoter	-110	DM992_28155	1&2	BYSR-1_peak_478	1.29764	5.93649	3.65155	Chr2:2225144-2225641	2225507	BYSR-2_peak_391	1.36959	7.46822	4.6468	Chr2:2225176-2225617	2225382	DM992_28155	WP_072438488.1|MULTISPECIES: hypothetical protein [Burkholderia]
Merged-Chr2-2212940-2	Chr2:2212630-2213247	promoter	-156	DM992_28090	1&2	BYSR-1_peak_477	1.3698	7.59426	4.86533	Chr2:2212630-2213247	2213142	BYSR-2_peak_390	1.33299	5.95044	3.56997	Chr2:2212695-2213189	2212976	DM992_28090	WP_174422014.1|hypothetical protein [Burkholderia diffusa]
Merged-Chr2-2177519-2	Chr2:2177312-2177680	promoter	-42	DM992_27915	1&2	BYSR-1_peak_475	1.37812	7.69266	4.93938	Chr2:2177414-2177671	2177554	BYSR-2_peak_388	1.32833	5.48465	3.23848	Chr2:2177312-2177680	2177627	DM992_27915	AIO34800.1|hypothetical protein DM39_4667 [Burkholderia cenocepacia]
Merged-Chr2-2176887-2	Chr2:2176704-2177041	promoter	303	DM992_27910	1&2	BYSR-1_peak_474	1.20268	2.99291	1.54097	Chr2:2176704-2177010	2176721	BYSR-2_peak_387	1.25833	3.86583	2.07981	Chr2:2176795-2177041	2177019	DM992_27910	WP_040130507.1|DUF1911 domain-containing protein [Burkholderia cenocepacia]
Merged-Chr2-2175620-2	Chr2:2175379-2175826	promoter	-49	DM992_27900	1&2	BYSR-1_peak_473	1.40283	8.55064	5.56747	Chr2:2175514-2175762	2175668	BYSR-2_peak_385	1.47943	10.2079	6.49134	Chr2:2175379-2175826	2175663	DM992_27900	WP_155754315.1|hypothetical protein [Burkholderia multivorans]
Merged-Chr2-2174145-2	Chr2:2173897-2174536	promoter	-19	DM992_27890	1&2	BYSR-1_peak_472	1.22144	3.55035	1.93644	Chr2:2173911-2174236	2174127	BYSR-2_peak_384	1.35433	6.28473	3.81062	Chr2:2173897-2174536	2174267	DM992_27890	WP_106409001.1|YgcG family protein [Burkholderia puraquae]
Merged-Chr2-2173027-3	Chr2:2172300-2173592	promoter	125	DM992_27875	1&2	BYSR-1_peak_471	1.37114	7.57306	4.84964	Chr2:2172790-2173592	2172847	BYSR-2_peak_382, BYSR-2_peak_383	-	-	-	-	-	DM992_27875	WP_105393332.1|MULTISPECIES: hypothetical protein [Burkholderia cepacia complex]
Merged-Chr2-2145475-2	Chr2:2145215-2145790	promoter	-404	DM992_27765	1&2	BYSR-1_peak_469	1.32399	6.18172	3.82948	Chr2:2145215-2145575	2145514	BYSR-2_peak_380	1.38423	7.29371	4.52416	Chr2:2145321-2145790	2145460	DM992_27765	WP_175960427.1|peptidase S10 [Burkholderia pyrrocinia]
Merged-Chr2-2133800-2	Chr2:2133573-2134200	promoter	-36	DM992_27700	1&2	BYSR-1_peak_468	1.48514	11.4159	7.73778	Chr2:2133573-2134200	2133731	BYSR-2_peak_379	1.27023	3.95031	2.13845	Chr2:2133584-2133845	2133691	DM992_27700	WP_174416679.1|SDR family oxidoreductase [Burkholderia diffusa]
Merged-Chr2-2124187-2	Chr2:2123830-2124571	promoter	56	DM992_27670	1&2	BYSR-1_peak_466	1.36422	7.04082	4.46197	Chr2:2123852-2124571	2123960	BYSR-2_peak_378	1.35764	6.09943	3.67744	Chr2:2123830-2124497	2124024	DM992_27670	WP_175917367.1|cache domain-containing protein [Burkholderia pyrrocinia]
Merged-Chr2-2096247-2	Chr2:2096118-2096396	promoter	16	DM992_27510	1&2	BYSR-1_peak_465	1.40855	9.16759	6.03723	Chr2:2096118-2096342	2096191	BYSR-2_peak_377	1.56188	13.5495	8.59448	Chr2:2096134-2096396	2096255	DM992_27510	WP_174910054.1|hypothetical protein [Burkholderia diffusa]
Merged-Chr2-2075107-2	Chr2:2074863-2075463	promoter	-360	DM992_27410	1&2	BYSR-1_peak_463	1.27524	4.72697	2.7766	Chr2:2074943-2075161	2075098	BYSR-2_peak_376	1.45807	9.15865	5.80528	Chr2:2074863-2075463	2075319	DM992_27410	TWF02949.1|hemolysin activation/secretion protein [Burkholderia cepacia]
Merged-Chr2-2070341-2	Chr2:2069940-2070634	promoter	-386	DM992_27395	1&2	BYSR-1_peak_461	1.24032	3.72918	2.06314	Chr2:2069940-2070405	2069959	BYSR-2_peak_375	1.26667	4.01202	2.18339	Chr2:2070388-2070634	2070427	DM992_27395	WP_060158326.1|type I secretion system permease/ATPase [Burkholderia cepacia]
Merged-Chr2-2067713-2	Chr2:2066167-2069420	promoter	-1830	DM992_27390	1&2	BYSR-1_peak_460	1.36504	7.80898	5.02496	Chr2:2066167-2069420	2066560	BYSR-2_peak_374	1.36778	7.25742	4.49917	Chr2:2066210-2069055	2066512	DM992_27390	WP_124823683.1|MULTISPECIES: transposase [Burkholderia cepacia complex]
Merged-Chr2-2060236-2	Chr2:2060019-2060399	promoter	-215	DM992_27365	1&2	BYSR-1_peak_459	1.52771	12.6662	8.73177	Chr2:2060149-2060399	2060290	BYSR-2_peak_373	1.5689	12.1537	7.73835	Chr2:2060019-2060378	2060326	DM992_27365	WP_175746821.1|LysE family translocator [Burkholderia pyrrocinia]
Merged-Chr2-1999840-2	Chr2:1999682-1999987	promoter	-124	DM992_27020	1&2	BYSR-1_peak_456	1.52595	12.1591	8.32105	Chr2:1999682-1999987	1999862	BYSR-2_peak_370	1.46198	8.67044	5.46427	Chr2:1999716-1999975	1999923	DM992_27020	WP_131948176.1|2-keto-3-deoxygluconate transporter [Burkholderia pyrrocinia]
Merged-Chr2-1952125-2	Chr2:1951757-1952508	promoter	-44	DM992_26780	1&2	BYSR-1_peak_454	1.36996	6.91661	4.37203	Chr2:1951757-1952445	1951916	BYSR-2_peak_369	1.34232	5.84711	3.50017	Chr2:1951792-1952508	1952439	DM992_26780	WP_072438495.1|MULTISPECIES: hypothetical protein [Burkholderia]
Merged-Chr2-1948999-2	Chr2:1948372-1949540	promoter	121	DM992_26745	1&2	BYSR-1_peak_453	1.38025	7.08294	4.49408	Chr2:1948836-1949540	1949078	BYSR-2_peak_368	1.35721	6.20378	3.75387	Chr2:1948372-1949250	1948503	DM992_26745	WP_175959809.1|Crp/Fnr family transcriptional regulator [Burkholderia pyrrocinia]
Merged-Chr2-1940506-2	Chr2:1940128-1941031	TTS (JP2270_GM005687_rna)	573	DM992_26700	1&2	BYSR-1_peak_451	1.40218	8.02769	5.18516	Chr2:1940128-1940726	1940198	BYSR-2_peak_367	1.35543	5.81998	3.47743	Chr2:1940141-1941031	1940237	DM992_26700	WP_105391355.1|universal stress protein [Burkholderia cepacia]
Merged-Chr2-1930007-2	Chr2:1929661-1930419	promoter	-241	DM992_26640	1&2	BYSR-1_peak_450	1.38591	7.29535	4.6475	Chr2:1929661-1930419	1930005	BYSR-2_peak_365	1.42254	7.93608	4.96295	Chr2:1929756-1930195	1930088	DM992_26640	WP_175746976.1|hypothetical protein [Burkholderia pyrrocinia]
Merged-Chr2-1878030-3	Chr2:1877397-1878629	promoter	-439	DM992_26350	1&2	BYSR-1_peak_442	1.53549	12.9617	8.97722	Chr2:1877397-1878629	1878328	BYSR-2_peak_357, BYSR-2_peak_358	-	-	-	-	-	DM992_26350	WP_080323111.1|MULTISPECIES: TniB family NTP-binding protein [Burkholderia]
Merged-Chr2-1872531-2	Chr2:1872195-1872806	TTS (JP2270_GM005608_rna)	976	DM992_26325	1&2	BYSR-1_peak_438	1.65626	15.8847	11.2685	Chr2:1872195-1872806	1872747	BYSR-2_peak_353	1.34395	5.364	3.15041	Chr2:1872328-1872796	1872411	DM992_26325	WP_080484403.1|MULTISPECIES: IS30 family transposase [Burkholderia]
Merged-Chr2-1868738-3	Chr2:1868010-1869565	promoter	312	DM992_26310	1&2	BYSR-1_peak_436	1.38355	6.97749	4.41467	Chr2:1868035-1869565	1868154	BYSR-2_peak_351, BYSR-2_peak_352	-	-	-	-	-	DM992_26310	WP_059743231.1|alpha/beta fold hydrolase [Burkholderia ubonensis]
Merged-Chr2-1866701-3	Chr2:1865617-1867468	promoter	-795	DM992_26305	1&2	BYSR-1_peak_435, BYSR-1_peak_434	-	-	-	-	-	BYSR-2_peak_350	1.35517	5.85186	3.50167	Chr2:1865617-1867463	1866502	DM992_26305	WP_143134552.1|DNA adenine methylase [Burkholderia ubonensis]
Merged-Chr2-1864136-2	Chr2:1862906-1865236	exon (JP2270_GM005604_rna, exon 1 of 1)	730	DM992_26295	1&2	BYSR-1_peak_433	1.39729	7.68856	4.93668	Chr2:1864191-1865236	1864986	BYSR-2_peak_349	1.31693	5.06499	2.93717	Chr2:1862906-1864213	1863216	DM992_26295	WP_080407288.1|diguanylate cyclase [Burkholderia ubonensis]
Merged-Chr2-1833613-2	Chr2:1833296-1833942	promoter	-385	DM992_26140	1&2	BYSR-1_peak_428	1.41207	7.9258	5.1083	Chr2:1833296-1833897	1833672	BYSR-2_peak_345	1.39991	6.55249	4.006	Chr2:1833320-1833942	1833373	DM992_26140	WP_105391311.1|FHA domain-containing protein [Burkholderia cepacia]
Merged-Chr2-1822001-2	Chr2:1821754-1822183	TTS (JP2270_GM005561_rna)	579	DM992_26060	1&2	BYSR-1_peak_427	1.23327	3.23641	1.71378	Chr2:1821754-1822115	1822022	BYSR-2_peak_343	1.34488	5.22085	3.04874	Chr2:1821954-1822183	1822101	DM992_26060	WP_175718158.1|nuclear transport factor 2 family protein [Burkholderia anthina]
Merged-Chr2-1776439-2	Chr2:1776237-1776668	promoter	-16	DM992_25845	1&2	BYSR-1_peak_423	1.40459	8.1417	5.26655	Chr2:1776237-1776477	1776395	BYSR-2_peak_341	1.2833	4.277	2.37103	Chr2:1776374-1776668	1776448	DM992_25845	WP_174379542.1|hypothetical protein [Burkholderia pyrrocinia]

Merged-Chr2-1757028-2	Chr2:1756550-1757495	promoter	-534	DM992_25785	1&2	BYSR-1_peak_422	1.34483	6.39264	3.98469	Chr2:1756550-1757495	1756678	BYSR-2_peak_337	1.34321	5.70717	3.39823	Chr2:1756650-1757417	1756951	DM992_25785	WP_174433457.1|ShlB/FhaC/HecB family hemolysin secretion/activation protein [Burkholderia metallica]
Merged-Chr2-1753995-2	Chr2:1753825-1754250	TTS (JP2270_GM005509_rna)	742	DM992_25780	1&2	BYSR-1_peak_421	1.40037	7.52173	4.81384	Chr2:1753826-1754250	1754114	BYSR-2_peak_336	1.26026	3.44363	1.78576	Chr2:1753825-1754082	1753838	DM992_25780	WP_175771183.1|DUF746 domain-containing protein [Burkholderia ambifaria]
Merged-Chr2-1731764-2	Chr2:1731577-1731932	promoter	151	DM992_25690	1&2	BYSR-1_peak_420	1.4031	7.40939	4.73268	Chr2:1731577-1731849	1731677	BYSR-2_peak_335	1.27205	3.6015	1.895	Chr2:1731698-1731932	1731733	DM992_25690	WP_096473226.1|Lrp/AsnC family transcriptional regulator [Burkholderia stabilis]
Merged-Chr2-1702104-2	Chr2:1701619-1702382	promoter	-393	DM992_25550	1&2	BYSR-1_peak_418	1.44808	9.82962	6.53217	Chr2:1701619-1702382	1702084	BYSR-2_peak_333	1.27965	4.22801	2.33524	Chr2:1702047-1702369	1702228	DM992_25550	WP_175942972.1|diaminopimelate epimerase [Burkholderia pyrrocinia]
Merged-Chr2-1698773-4	Chr2:1697171-1701408	promoter-TSS (JP2270_GM005459_rna)	762	DM992_25540	1&2	BYSR-1_peak_417	1.46686	10.4706	7.0118	Chr2:1697171-1701252	1697419	BYSR-2_peak_330, BYSR-2_peak_331, BYSR-2_peak_332	-	-	-	-	-	DM992_25540	WP_078041825.1|ATP-binding protein [Burkholderia cenocepacia]
Merged-Chr2-1693666-2	Chr2:1693335-1693973	promoter	221	DM992_25520	1&2	BYSR-1_peak_415	1.23587	3.66511	2.01849	Chr2:1693335-1693909	1693737	BYSR-2_peak_329	1.40318	7.41126	4.60562	Chr2:1693449-1693973	1693508	DM992_25520	WP_059854403.1|carbamoyltransferase [Burkholderia ubonensis]
Merged-Chr2-1690494-2	Chr2:1690253-1690731	promoter	-182	DM992_25510	1&2	BYSR-1_peak_414	1.50932	11.6287	7.90156	Chr2:1690253-1690731	1690531	BYSR-2_peak_327	1.304	4.53157	2.55355	Chr2:1690382-1690613	1690424	DM992_25510	WP_006762471.1|MBL fold metallo-hydrolase [Burkholderia ambifaria]
Merged-Chr2-1684067-3	Chr2:1683350-1684922	promoter	-606	DM992_25485	1&2	BYSR-1_peak_409	1.41988	8.7978	5.75359	Chr2:1683375-1684914	1683579	BYSR-2_peak_325, BYSR-2_peak_326	-	-	-	-	-	DM992_25485	WP_174999924.1|TauD/TfdA family dioxygenase [Burkholderia lata]
Merged-Chr2-1664092-3	Chr2:1662932-1665499	promoter	-191	DM992_25410	1&2	BYSR-1_peak_407	1.4474	10.267	6.85825	Chr2:1663030-1665499	1664676	BYSR-2_peak_323, BYSR-2_peak_324	-	-	-	-	-	DM992_25410	WP_071333832.1|hypothetical protein [Burkholderia contaminans]
Merged-Chr2-1649027-2	Chr2:1648772-1649272	promoter	-596	DM992_25315	1&2	BYSR-1_peak_405	1.43028	9.15717	6.02898	Chr2:1648826-1649239	1649176	BYSR-2_peak_322	1.38179	6.72096	4.1256	Chr2:1648772-1649272	1649176	DM992_25315	ABF80186.1|acylphosphatase [Burkholderia cenocepacia AU 1054]
Merged-Chr2-1600174-2	Chr2:1599861-1600432	TTS (JP2270_GM005375_rna)	1173	DM992_25085	1&2	BYSR-1_peak_403	1.19393	2.71864	1.3487	Chr2:1600021-1600385	1600158	BYSR-2_peak_320	1.36626	6.04621	3.63937	Chr2:1599861-1600432	1600117	DM992_25085	WP_047902235.1|MULTISPECIES: MgtC/SapB family protein [Burkholderia cepacia complex]
Merged-Chr2-1597746-2	Chr2:1597497-1597931	promoter	-127	DM992_25070	1&2	BYSR-1_peak_401	1.34822	6.59056	4.13159	Chr2:1597645-1597931	1597720	BYSR-2_peak_318	1.28814	4.47172	2.50919	Chr2:1597497-1597914	1597704	DM992_25070	WP_011546093.1|MULTISPECIES: efflux RND transporter periplasmic adaptor subunit [Burkholderia]
Merged-Chr2-1466418-2	Chr2:1466137-1466724	promoter	-37	DM992_24475	1&2	BYSR-1_peak_394	1.26453	4.03921	2.28537	Chr2:1466137-1466515	1466422	BYSR-2_peak_315	1.35395	5.71102	3.39942	Chr2:1466297-1466724	1466409	DM992_24475	WP_034181737.1|PAS domain-containing methyl-accepting chemotaxis protein [Burkholderia pyrrocinia]
Merged-Chr2-1442849-2	Chr2:1442311-1443403	promoter	-504	DM992_24385	1&2	BYSR-1_peak_393	1.43726	8.4016	5.45787	Chr2:1442770-1443403	1443192	BYSR-2_peak_314	1.32758	4.81714	2.758	Chr2:1442311-1442913	1442777	DM992_24385	WP_059599090.1|MULTISPECIES: alpha/beta hydrolase [Burkholderia]
Merged-Chr2-1437696-2	Chr2:1437071-1438321	promoter	256	DM992_24350	1&2	BYSR-1_peak_391	1.43274	8.35813	5.42525	Chr2:1437149-1438321	1437325	BYSR-2_peak_312	1.28757	4.09404	2.24034	Chr2:1437071-1438245	1437460	DM992_24350	WP_060346242.1|MULTISPECIES: hypothetical protein [Burkholderia]
Merged-Chr2-1339578-2	Chr2:1339426-1339749	promoter	-255	DM992_23915	1&2	BYSR-1_peak_390	1.23635	3.20428	1.69021	Chr2:1339430-1339708	1339536	BYSR-2_peak_311	1.43733	7.27729	4.51275	Chr2:1339426-1339749	1339526	DM992_23915	WP_175769740.1|IS5 family transposase [Burkholderia cenocepacia]
Merged-Chr2-1337543-3	Chr2:1336751-1338068	promoter	280	DM992_23905	1&2	BYSR-1_peak_389	1.32332	5.71026	3.48769	Chr2:1336751-1338003	1337680	BYSR-2_peak_309, BYSR-2_peak_310	-	-	-	-	-	DM992_23905	WP_080970853.1|hypothetical protein [Burkholderia cepacia]
Merged-Chr2-1293351-2	Chr2:1293109-1293536	promoter	-248	DM992_23705	1&2	BYSR-1_peak_388	1.34985	5.80505	3.55632	Chr2:1293109-1293471	1293358	BYSR-2_peak_308	1.28009	3.8345	2.05716	Chr2:1293288-1293536	1293497	DM992_23705	ABB10549.1|diguanylate phosphodiesterase [Burkholderia lata]
Merged-Chr2-126644-3	Chr2:126245-127172	promoter	498	DM992_18220	1&2	BYSR-1_peak_288, BYSR-1_peak_287	-	-	-	-	-	BYSR-2_peak_229	1.39359	6.81132	4.18785	Chr2:126273-126905	126522	DM992_18220	WP_124617331.1|helix-turn-helix domain-containing protein [Burkholderia contaminans]
Merged-Chr2-125758-2	Chr2:125508-125972	promoter	441	DM992_18215	1&2	BYSR-1_peak_286	1.27857	4.42367	2.55754	Chr2:125508-125870	125530	BYSR-2_peak_228	1.28326	4.07792	2.22854	Chr2:125682-125972	125909	DM992_18215	WP_048022524.1|MULTISPECIES: carbon-nitrogen hydrolase family protein [Burkholderia]
Merged-Chr2-1173284-2	Chr2:1173074-1173511	promoter	-299	DM992_23200	1&2	BYSR-1_peak_381	1.52866	13.531	9.43473	Chr2:1173116-1173436	1173251	BYSR-2_peak_303	1.49642	10.841	6.88906	Chr2:1173074-1173511	1173156	DM992_23200	WP_174417613.1|MULTISPECIES: transcriptional regulator BetI [Burkholderia cepacia complex]
Merged-Chr2-1157523-2	Chr2:1157293-1157758	promoter	-123	DM992_23140	1&2	BYSR-1_peak_380	1.31379	6.12423	3.78944	Chr2:1157293-1157720	1157624	BYSR-2_peak_302	1.36724	6.62654	4.06002	Chr2:1157323-1157758	1157715	DM992_23140	WP_114179127.1|GlxA family transcriptional regulator [Burkholderia pyrrocinia]
Merged-Chr2-114880-3	Chr2:114334-115740	promoter	-924	DM992_18165	1&2	BYSR-1_peak_282, BYSR-1_peak_281	-	-	-	-	-	BYSR-2_peak_224	1.42292	8.02043	5.01895	Chr2:114508-115740	114890	DM992_18165	WP_126225775.1|hypothetical protein [Burkholderia ambifaria]
Merged-Chr2-1136261-2	Chr2:1135929-1136525	promoter	104	DM992_23050	1&2	BYSR-1_peak_378	1.33715	6.36669	3.96545	Chr2:1135929-1136525	1136185	BYSR-2_peak_301	1.30352	4.66939	2.65162	Chr2:1136103-1136490	1136443	DM992_23050	WP_146254505.1|hypothetical protein [Burkholderia cepacia]
Merged-Chr2-1135463-2	Chr2:1135163-1135660	promoter	-76	DM992_23045	1&2	BYSR-1_peak_377	1.31416	5.81056	3.56042	Chr2:1135163-1135648	1135200	BYSR-2_peak_300	1.21865	3.11161	1.55777	Chr2:1135384-1135660	1135610	DM992_23045	WP_034180733.1|hypothetical protein [Burkholderia pyrrocinia]
Merged-Chr2-1100743-2	Chr2:1100604-1100876	promoter	25	DM992_22870	1&2	BYSR-1_peak_374	1.23304	3.29952	1.75731	Chr2:1100604-1100869	1100700	BYSR-2_peak_299	1.38986	6.72216	4.12643	Chr2:1100625-1100876	1100817	DM992_22870	WP_059447722.1|MULTISPECIES: DUF4148 domain-containing protein [Burkholderia]
Merged-Chr2-1076518-2	Chr2:1075229-1077458	promoter	-741	DM992_22750	1&2	BYSR-1_peak_372	1.55219	14.8582	10.4615	Chr2:1075971-1077458	1076711	BYSR-2_peak_297	1.39051	7.98613	4.99894	Chr2:1075229-1077415	1076794	DM992_22750	WP_175849793.1|IS3 family transposase [Burkholderia cepacia]
Merged-Chr2-1058344-2	Chr2:1058067-1058670	promoter	-691	DM992_22660	1&2	BYSR-1_peak_371	1.32395	6.40771	3.99569	Chr2:1058075-1058670	1058272	BYSR-2_peak_296	1.27513	4.4243	2.47632	Chr2:1058067-1058564	1058282	DM992_22660	WP_051983680.1|type VI secretion system tip protein VgrG [Burkholderia pyrrocinia]
Merged-Chr2-1057250-2	Chr2:1056678-1057818	promoter	429	DM992_22660	1&2	BYSR-1_peak_370	1.50822	12.6739	8.73867	Chr2:1056678-1057818	1057337	BYSR-2_peak_295	1.40325	7.73577	4.82701	Chr2:1056698-1057808	1057082	DM992_22660	WP_051983680.1|type VI secretion system tip protein VgrG [Burkholderia pyrrocinia]
Merged-Chr2-1054247-2	Chr2:1053988-1054539	promoter	85	DM992_22650	1&2	BYSR-1_peak_368	1.30823	5.67166	3.45901	Chr2:1053988-1054235	1054102	BYSR-2_peak_294	1.26054	4.18601	2.3051	Chr2:1054228-1054539	1054443	DM992_22650	WP_081938416.1|LysM peptidoglycan-binding domain-containing protein [Burkholderia pyrrocinia]
Merged-Chr2-1052610-2	Chr2:1052083-1053098	promoter	-2	DM992_22645	1&2	BYSR-1_peak_367	1.30222	5.69073	3.47304	Chr2:1052214-1053098	1052339	BYSR-2_peak_293	1.33299	5.95044	3.56997	Chr2:1052083-1053046	1052268	DM992_22645	WP_034180658.1|hypothetical protein [Burkholderia pyrrocinia]
Merged-Chr2-1051357-2	Chr2:1050904-1051769	promoter	416	DM992_22635	1&2	BYSR-1_peak_366	1.29226	5.3593	3.23298	Chr2:1051159-1051598	1051403	BYSR-2_peak_292	1.46658	10.4906	6.66378	Chr2:1050904-1051769	1051613	DM992_22635	WP_034180657.1|hypothetical protein [Burkholderia pyrrocinia]
Merged-Chr1-958146-2	Chr1:957889-958648	promoter	-464	DM992_04575	1&2	BYSR-1_peak_81	1.32866	4.95635	2.94089	Chr1:957898-958149	958078	BYSR-2_peak_61	1.35587	5.63968	3.35031	Chr1:957889-958648	958553	DM992_04575	WP_175963332.1|sensor histidine kinase [Burkholderia pyrrocinia]
Merged-Chr1-928808-2	Chr1:928288-929161	promoter	102	DM992_04420	1&2	BYSR-1_peak_79	1.25109	3.69881	2.04078	Chr1:928799-929161	928867	BYSR-2_peak_60	1.2862	4.2562	2.35625	Chr1:928288-928985	928658	DM992_04420	WP_175919546.1|MFS transporter [Burkholderia pyrrocinia]

Merged-Chr1-927507-2	Chr1:927206-927926	promoter	-55	DM992_04415	1&2	BYSR-1_peak_78	1.28096	4.82533	2.84644	Chr1:927253-927926	927663	BYSR-2_peak_59	1.575	11.8184	7.53239	Chr1:927206-927643	927354	DM992_04415	WP_175919547.1|radical SAM protein [Burkholderia pyrrocinia]
Merged-Chr1-913699-2	Chr1:913521-913884	promoter	22	DM992_04350	1&2	BYSR-1_peak_76	1.21481	2.91383	1.48562	Chr1:913521-913808	913667	BYSR-2_peak_57	1.33031	4.95651	2.85796	Chr1:913583-913884	913861	DM992_04350	WP_175963376.1|helix-turn-helix transcriptional regulator [Burkholderia pyrrocinia]
Merged-Chr1-872573-2	Chr1:872400-872751	exon (JP2270_GM000803_rna, exon 1 of 1)	1089	DM992_04145	1&2	BYSR-1_peak_75	1.28771	4.02884	2.27776	Chr1:872400-872751	872620	BYSR-2_peak_56	1.33911	4.67728	2.65714	Chr1:872457-872685	872620	DM992_04145	WP_175963281.1|DNA topoisomerase III [Burkholderia pyrrocinia]
Merged-Chr1-834943-2	Chr1:834775-835109	promoter	-58	DM992_03965	1&2	BYSR-1_peak_71	1.32632	5.12386	3.06344	Chr1:834775-835108	835065	BYSR-2_peak_52	1.33474	4.82226	2.76158	Chr1:834782-835109	835090	DM992_03965	WP_174420241.1|MULTISPECIES: transcriptional regulator [Burkholderia cepacia complex]
Merged-Chr1-827568-2	Chr1:827342-827725	TTS (JP2270_GM000765_rna)	696	DM992_03930	1&2	BYSR-1_peak_68	1.31758	4.92075	2.91721	Chr1:827342-827719	827440	BYSR-2_peak_51	1.25451	3.24313	1.64822	Chr1:827489-827725	827615	DM992_03930	WP_047899369.1|MULTISPECIES: DNA topoisomerase (ATP-hydrolyzing) subunit B [Burkholderia cepacia complex]
Merged-Chr1-823250-4	Chr1:821942-824354	TTS (JP2270_GM000760_rna)	986	DM992_03920	1&2	BYSR-1_peak_67, BYSR-1_peak_66	-	-	-	-	-	BYSR-2_peak_49, BYSR-2_peak_50	-	-	-	-	-	DM992_03920	WP_175766264.1|hypothetical protein [Burkholderia ambifaria]
Merged-Chr1-816251-2	Chr1:816114-816392	TTS (JP2270_GM000759_rna)	783	DM992_03885	1&2	BYSR-1_peak_65	1.41888	7.25289	4.61502	Chr1:816115-816392	816341	BYSR-2_peak_47	1.39913	6.00992	3.61042	Chr1:816114-816383	816331	DM992_03885	WP_175962629.1|cytochrome b [Burkholderia pyrrocinia]
Merged-Chr1-811409-2	Chr1:811206-811641	promoter	-122	DM992_03860	1&2	BYSR-1_peak_63	1.35384	5.65203	3.44507	Chr1:811271-811641	811492	BYSR-2_peak_46	1.46972	7.81873	4.88229	Chr1:811206-811520	811341	DM992_03860	WP_174418356.1|hypothetical protein [Burkholderia diffusa]
Merged-Chr1-791838-2	Chr1:791667-792015	promoter	19	DM992_03770	1&2	BYSR-1_peak_62	1.25114	3.60208	1.97222	Chr1:791689-791984	791789	BYSR-2_peak_45	1.29932	4.03127	2.19729	Chr1:791667-792015	791688	DM992_03770	WP_175704564.1|response regulator transcription factor [Burkholderia ambifaria]
Merged-Chr1-789356-2	Chr1:788219-790382	promoter	-540	DM992_03755	1&2	BYSR-1_peak_61	1.46603	10.0136	6.67019	Chr1:788219-790382	789080	BYSR-2_peak_44	1.54364	11.6406	7.41914	Chr1:788453-790373	789437	DM992_03755	WP_174917334.1|hypothetical protein [Burkholderia lata]
Merged-Chr1-723625-2	Chr1:723434-723844	promoter	-81	DM992_03410	1&2	BYSR-1_peak_60	1.41339	7.84153	5.0496	Chr1:723488-723844	723739	BYSR-2_peak_40	1.23825	3.09792	1.54802	Chr1:723434-723737	723513	DM992_03410	EAY63185.1|ATP synthase C chain [Burkholderia cenocepacia PC184]
Merged-Chr1-704103-2	Chr1:703869-704378	promoter-TSS (JP2270_GM000653_rna)	610	DM992_03330	1&2	BYSR-1_peak_59	1.43921	8.11158	5.24497	Chr1:703888-704378	704225	BYSR-2_peak_39	1.36025	5.36616	3.15188	Chr1:703869-704279	703963	DM992_03330	WP_131948682.1|glutathione S-transferase [Burkholderia pyrrocinia]
Merged-Chr1-638614-2	Chr1:638484-638747	promoter	-72	DM992_03070	1&2	BYSR-1_peak_57	1.29641	4.25039	2.43543	Chr1:638484-638732	638588	BYSR-2_peak_35	1.43122	6.80405	4.18293	Chr1:638496-638747	638574	DM992_03070	WP_006401410.1|MULTISPECIES: 30S ribosomal protein S21 [Burkholderiaceae]
Merged-Chr1-598075-2	Chr1:597860-598260	promoter	-81	DM992_02870	1&2	BYSR-1_peak_55	1.4791	9.86793	6.56135	Chr1:597972-598260	598026	BYSR-2_peak_32	1.43364	7.47819	4.65082	Chr1:597860-598208	598030	DM992_02870	PXX27166.1|H-NS family DNA-binding protein [Burkholderia pyrrocinia]
Merged-Chr1-588871-2	Chr1:588617-589099	TTS (JP2270_GM000550_rna)	567	DM992_02810	1&2	BYSR-1_peak_54	1.39541	6.67578	4.1934	Chr1:588835-589099	588954	BYSR-2_peak_31	1.39292	6.03942	3.63351	Chr1:588617-588933	588786	DM992_02810	WP_175771190.1|hypothetical protein [Burkholderia ambifaria]
Merged-Chr1-587509-2	Chr1:587241-587732	promoter	379	DM992_02805	1&2	BYSR-1_peak_52	1.32437	5.06554	3.01958	Chr1:587241-587732	587354	BYSR-2_peak_30	1.41407	6.77655	4.16653	Chr1:587374-587689	587552	DM992_02805	WP_175771190.1|hypothetical protein [Burkholderia ambifaria]
Merged-Chr1-579817-2	Chr1:579602-580033	TTS (JP2270_GM000543_rna)	646	DM992_02775	1&2	BYSR-1_peak_49	1.36861	6.46093	4.03687	Chr1:579602-579926	579723	BYSR-2_peak_27	1.57756	12.0089	7.65601	Chr1:579707-580033	579866	DM992_02775	WP_059952602.1|proline dehydrogenase family protein [Burkholderia ubonensis]
Merged-Chr1-567795-2	Chr1:567355-568105	promoter	-804	DM992_02715	1&2	BYSR-1_peak_45	1.30059	4.63695	2.71246	Chr1:567355-568024	567686	BYSR-2_peak_22	1.36003	5.49771	3.24862	Chr1:567696-568105	567859	DM992_02715	WP_174389411.1|TniB family NTP-binding protein [Burkholderia metallica]
Merged-Chr1-490900-2	Chr1:490679-491252	promoter	149	DM992_02355	1&2	BYSR-1_peak_40	1.37947	6.18099	3.82895	Chr1:490679-491252	491158	BYSR-2_peak_19	1.37345	5.40891	3.18243	Chr1:490708-490962	490778	DM992_02355	WP_004199272.1|MULTISPECIES: 50S ribosomal protein L22 [Burkholderiaceae]
Merged-Chr1-401162-2	Chr1:400839-401798	promoter	-53	DM992_01830	1&2	BYSR-1_peak_37	1.36837	5.97388	3.67993	Chr1:400896-401118	401082	BYSR-2_peak_18	1.43741	7.77758	4.85532	Chr1:400839-401798	401645	DM992_01830	KVC58881.1|hypothetical protein WI72_14000 [Burkholderia ubonensis]
Merged-Chr1-392627-2	Chr1:392127-393161	promoter	447	DM992_01800	1&2	BYSR-1_peak_32	1.42171	7.46915	4.77529	Chr1:392127-392887	392756	BYSR-2_peak_15	1.33853	4.71961	2.68629	Chr1:392333-393161	392723	DM992_01800	WP_174393674.1|restriction endonuclease subunit S [Burkholderia cepacia]
Merged-Chr1-388604-2	Chr1:388366-388946	promoter	-1467	DM992_01790	1&2	BYSR-1_peak_30	1.28115	3.90629	2.19084	Chr1:388409-388697	388438	BYSR-2_peak_14	1.42874	6.75088	4.14874	Chr1:388366-388946	388917	DM992_01790	WP_165948012.1|ShlB/FhaC/HecB family hemolysin secretion/activation protein [Burkholderia pyrrocinia]
Merged-Chr1-387475-2	Chr1:386598-387969	promoter	-94	DM992_01790	1&2	BYSR-1_peak_29	1.45266	8.23654	5.33389	Chr1:386598-387969	387600	BYSR-2_peak_13	1.40554	6.16496	3.72215	Chr1:387488-387846	387540	DM992_01790	WP_165948012.1|ShlB/FhaC/HecB family hemolysin secretion/activation protein [Burkholderia pyrrocinia]
Merged-Chr1-374729-2	Chr1:374466-374920	promoter	92	DM992_01775	1&2	BYSR-1_peak_27	1.3037	4.59941	2.68488	Chr1:374466-374914	374876	BYSR-2_peak_12	1.3758	5.78771	3.45374	Chr1:374618-374920	374880	DM992_01775	WP_124519266.1|hypothetical protein [Burkholderia stagnalis]
Merged-Chr1-37078-2	Chr1:36321-37596	promoter	460	DM992_00145	1&2	BYSR-1_peak_10	1.33509	5.77945	3.53715	Chr1:37049-37347	37286	BYSR-2_peak_6	1.4037	6.96221	4.29757	Chr1:36321-37596	37255	DM992_00145	WP_059590844.1|dTDP-4-dehydrorhamnose 3,5-epimerase [Burkholderia cepacia]
Merged-Chr1-368894-2	Chr1:368587-369313	promoter	470	DM992_01730	1&2	BYSR-1_peak_26	1.41252	7.36465	4.70059	Chr1:368587-369081	368725	BYSR-2_peak_11	1.30343	4.1054	2.24864	Chr1:368596-369313	368649	DM992_01730	WP_105393972.1|MULTISPECIES: hypothetical protein [Burkholderia cepacia complex]
Merged-Chr1-3684201-2	Chr1:3684013-3684372	promoter	-295	DM992_17470	1&2	BYSR-1_peak_272	1.5826	13.3576	9.29521	Chr1:3684013-3684372	3684117	BYSR-2_peak_210	1.36542	5.53097	3.27092	Chr1:3684049-3684372	3684117	DM992_17470	WP_034182847.1|MULTISPECIES: LysR family transcriptional regulator [Burkholderia cepacia complex]
Merged-Chr1-3649705-2	Chr1:3649437-3650108	promoter	365	DM992_17310	1&2	BYSR-1_peak_270	1.39162	6.54351	4.0965	Chr1:3649437-3649796	3649550	BYSR-2_peak_208	1.37558	5.57548	3.30122	Chr1:3649481-3650108	3649700	DM992_17310	WP_031402778.1|MULTISPECIES: chaperonin GroEL [Burkholderia]
Merged-Chr1-3630448-2	Chr1:3630157-3630798	promoter	262	DM992_17215	1&2	BYSR-1_peak_268	1.44168	8.62598	5.62421	Chr1:3630157-3630798	3630467	BYSR-2_peak_207	1.34985	5.34238	3.13615	Chr1:3630199-3630640	3630598	DM992_17215	WP_069706985.1|glycosyltransferase [Burkholderia seminalis]
Merged-Chr1-3589013-2	Chr1:3588813-3589206	promoter	245	DM992_17010	1&2	BYSR-1_peak_264	1.28093	4.26747	2.44773	Chr1:3588905-3589206	3589172	BYSR-2_peak_205	1.36553	5.4934	3.24573	Chr1:3588813-3589129	3588924	DM992_17010	WP_047900015.1|MULTISPECIES: cupin domain-containing protein [Burkholderia]
Merged-Chr1-3476145-2	Chr1:3475926-3476427	promoter	140	DM992_16410	1&2	BYSR-1_peak_261	1.32879	4.92544	2.91807	Chr1:3475948-3476280	3475974	BYSR-2_peak_201	1.30927	4.0427	2.20333	Chr1:3475926-3476427	3476303	DM992_16410	WP_034183032.1|MULTISPECIES: DNA gyrase subunit A [Burkholderia]
Merged-Chr1-3377413-2	Chr1:3377041-3377642	promoter	-371	DM992_15895	1&2	BYSR-1_peak_257	1.34211	5.52115	3.34974	Chr1:3377041-3377642	3377285	BYSR-2_peak_197	1.28591	3.87383	2.08563	Chr1:3377362-3377610	3377515	DM992_15895	WP_034183110.1|MULTISPECIES: sigma-54-dependent Fis family transcriptional regulator [Burkholderia]

Merged-Chr1-3344819-2	Chr1:3344639-3344991	promoter	-208	DM992_15715	1&2	BYSR-1_peak_255	1.33326	5.49656	3.33151	Chr1:3344671-3344991	3344852	BYSR-2_peak_196	1.4058	6.75537	4.15231	Chr1:3344639-3344975	3344795	DM992_15715	WP_072435964.1|MULTISPECIES: LLM class flavin-dependent oxidoreductase [Burkholderia]
Merged-Chr1-3334835-2	Chr1:3334470-3335181	promoter	64	DM992_15660	1&2	BYSR-1_peak_253	1.35507	6.01975	3.71375	Chr1:3334470-3335181	3334546	BYSR-2_peak_194	1.46777	8.26892	5.19045	Chr1:3334637-3335055	3334902	DM992_15660	BAX60286.1|membrane protein [Burkholderia stabilis]
Merged-Chr1-3333595-2	Chr1:3333379-3333788	promoter	-564	DM992_15655	1&2	BYSR-1_peak_252	1.28403	4.33177	2.49284	Chr1:3333379-3333788	3333490	BYSR-2_peak_192	1.27848	3.6888	1.95532	Chr1:3333427-3333786	3333439	DM992_15655	WP_096472845.1|hypothetical protein [Burkholderia stabilis]
Merged-Chr1-3319066-2	Chr1:3318868-3319345	promoter	196	DM992_15585	1&2	BYSR-1_peak_251	1.33433	5.65827	3.44952	Chr1:3318868-3319345	3318965	BYSR-2_peak_191	1.22274	2.77651	1.32859	Chr1:3318874-3319179	3318964	DM992_15585	WP_072435948.1|MULTISPECIES: NYN domain-containing protein [Burkholderia]
Merged-Chr1-32620-2	Chr1:32319-32884	promoter	-26	DM992_00125	1&2	BYSR-1_peak_7	1.36957	6.46431	4.03938	Chr1:32461-32817	32746	BYSR-2_peak_5	1.44008	7.44585	4.63122	Chr1:32319-32884	32480	DM992_00125	KUY66007.1|dTDP-glucose 4,6-dehydratase [Burkholderia cepacia]
Merged-Chr1-3211090-2	Chr1:3210930-3211230	promoter	142	DM992_15105	1&2	BYSR-1_peak_250	1.37465	7.06047	4.47666	Chr1:3210930-3211193	3211069	BYSR-2_peak_190	1.3601	5.94505	3.56581	Chr1:3211010-3211230	3211049	DM992_15105	WP_034183278.1|MULTISPECIES: ATP-dependent metallopeptidase FtsH/Yme1/Tma family protein [Burkholderia]
Merged-Chr1-3193966-2	Chr1:3193749-3194162	promoter	-39	DM992_15035	1&2	BYSR-1_peak_249	1.33407	5.49783	3.33229	Chr1:3193815-3194140	3193995	BYSR-2_peak_189	1.38015	6.06966	3.65536	Chr1:3193749-3194162	3193911	DM992_15035	WP_176114881.1|hypothetical protein [Burkholderia cepacia]
Merged-Chr1-3179831-2	Chr1:3179592-3180022	promoter	-662	DM992_14965	1&2	BYSR-1_peak_245	1.37185	7.06748	4.48216	Chr1:3179774-3180022	3179885	BYSR-2_peak_187	1.38408	6.71298	4.11978	Chr1:3179592-3179939	3179733	DM992_14965	WP_080410955.1|autoinducer binding domain-containing protein [Burkholderia ubonensis]
Merged-Chr1-3164814-2	Chr1:3164474-3165187	promoter	329	DM992_14880	1&2	BYSR-1_peak_240	1.40459	8.1417	5.26655	Chr1:3164474-3165082	3164737	BYSR-2_peak_185	1.39761	6.9531	4.29041	Chr1:3164515-3165187	3164633	DM992_14880	WP_175960396.1|DUF1330 domain-containing protein [Burkholderia pyrrocinia]
Merged-Chr1-3127721-3	Chr1:3126957-3128305	promoter	233	DM992_14750	1&2	BYSR-1_peak_238	1.38198	7.4662	4.77311	Chr1:3126957-3128278	3127924	BYSR-2_peak_183, BYSR-2_peak_184	-	-	-	-	-	DM992_14750	WP_027787815.1|hypothetical protein [Burkholderia cepacia]
Merged-Chr1-3116756-2	Chr1:3116588-3116939	promoter	-124	DM992_14710	1&2	BYSR-1_peak_237	1.37677	6.93008	4.38187	Chr1:3116588-3116892	3116702	BYSR-2_peak_182	1.34712	5.432	3.19798	Chr1:3116605-3116939	3116710	DM992_14710	WP_034183318.1|MULTISPECIES: porin [Burkholderia cepacia complex]
Merged-Chr1-2971387-2	Chr1:2971210-2971576	TTS (JP2270_GM002728_rna)	764	DM992_14020	1&2	BYSR-1_peak_231	1.20764	2.71677	1.34723	Chr1:2971270-2971576	2971271	BYSR-2_peak_179	1.24036	3.052	1.51759	Chr1:2971210-2971493	2971248	DM992_14020	WP_091926168.1|MULTISPECIES: DUF945 domain-containing protein [Burkholderia]
Merged-Chr1-2970660-2	Chr1:2970492-2970810	promoter	22	DM992_14020	1&2	BYSR-1_peak_230	1.34263	5.90808	3.63037	Chr1:2970581-2970810	2970769	BYSR-2_peak_178	1.44758	7.67468	4.78509	Chr1:2970492-2970759	2970600	DM992_14020	WP_091926168.1|MULTISPECIES: DUF945 domain-containing protein [Burkholderia]
Merged-Chr1-2947905-2	Chr1:2947734-2948097	promoter	-234	DM992_13920	1&2	BYSR-1_peak_227	1.31833	5.39733	3.26115	Chr1:2947734-2948038	2947867	BYSR-2_peak_176	1.3693	5.99462	3.6042	Chr1:2947752-2948097	2947828	DM992_13920	WP_105392886.1|hypothetical protein [Burkholderia cepacia]
Merged-Chr1-2937402-3	Chr1:2936903-2938074	TTS (JP2270_GM002692_rna)	672	DM992_13855	1&2	BYSR-1_peak_225	1.34537	5.55766	3.37679	Chr1:2936903-2937855	2937050	BYSR-2_peak_174, BYSR-2_peak_175	-	-	-	-	-	DM992_13855	RQT62264.1|hypothetical protein DF043_13830 [Burkholderia cepacia]
Merged-Chr1-2929919-2	Chr1:2929531-2930438	promoter	-1096	DM992_13810	1&2	BYSR-1_peak_221	1.30758	5.02584	2.99309	Chr1:2929846-2930438	2930096	BYSR-2_peak_173	1.32616	5.03767	2.91569	Chr1:2929531-2929862	2929687	DM992_13810	AQQ26679.1|hypothetical protein A8E88_13955 [Burkholderia cenocepacia]
Merged-Chr1-2925316-2	Chr1:2924956-2925617	promoter	-536	DM992_13800	1&2	BYSR-1_peak_219	1.30113	4.53186	2.63715	Chr1:2924956-2925352	2925273	BYSR-2_peak_170	1.35705	5.32065	3.11883	Chr1:2925342-2925617	2925379	DM992_13800	ONW43275.1|hypothetical protein A8E93_13695 [Burkholderia cenocepacia]
Merged-Chr1-2921049-2	Chr1:2920660-2921444	promoter	-46	DM992_13785	1&2	BYSR-1_peak_218	1.45702	8.96166	5.87685	Chr1:2920752-2921343	2921209	BYSR-2_peak_169	1.58298	11.5171	7.34099	Chr1:2920660-2921444	2921177	DM992_13785	WP_034199624.1|MULTISPECIES: hypothetical protein [Burkholderia]
Merged-Chr1-2919976-2	Chr1:2919453-2920445	TTS (JP2270_GM002680_rna)	1057	DM992_13785	1&2	BYSR-1_peak_217	1.39303	7.39625	4.72324	Chr1:2919453-2920445	2919598	BYSR-2_peak_168	1.47035	9.15117	5.79977	Chr1:2919671-2920335	2919902	DM992_13785	WP_034199624.1|MULTISPECIES: hypothetical protein [Burkholderia]
Merged-Chr1-2892986-2	Chr1:2892763-2893221	promoter	67	DM992_13660	1&2	BYSR-1_peak_215	1.46647	9.27761	6.11704	Chr1:2892892-2893221	2893002	BYSR-2_peak_165	1.36286	5.85125	3.50167	Chr1:2892763-2893071	2892910	DM992_13660	WP_072441703.1|MULTISPECIES: 2-oxoglutarate dehydrogenase E1 component [Burkholderia]
Merged-Chr1-2879508-2	Chr1:2879152-2879819	promoter	-320	DM992_13610	1&2	BYSR-1_peak_213	1.4886	9.86184	6.55664	Chr1:2879152-2879819	2879329	BYSR-2_peak_164	1.42784	7.31551	4.53696	Chr1:2879266-2879798	2879608	DM992_13610	WP_034184045.1|MULTISPECIES: Flp family type IVb pilin [Burkholderia cepacia complex]
Merged-Chr1-2868239-2	Chr1:2867979-2868473	promoter	-324	DM992_13550	1&2	BYSR-1_peak_211	1.36526	6.3793	3.97475	Chr1:2867979-2868463	2868122	BYSR-2_peak_162	1.28382	3.80952	2.03935	Chr1:2868044-2868473	2868141	DM992_13550	WP_105393206.1|sigma-54-dependent Fis family transcriptional regulator [Burkholderia cepacia]
Merged-Chr1-2841953-2	Chr1:2841709-2842272	promoter	-21	DM992_13425	1&2	BYSR-1_peak_209	1.39636	6.63029	4.15855	Chr1:2841892-2842272	2842092	BYSR-2_peak_161	1.38898	5.80349	3.46397	Chr1:2841709-2841942	2841778	DM992_13425	WP_034184071.1|MULTISPECIES: FadR family transcriptional regulator [Burkholderia]
Merged-Chr1-2790639-2	Chr1:2790335-2790970	promoter	141	DM992_13120	1&2	BYSR-1_peak_207	1.35932	5.79211	3.54664	Chr1:2790335-2790970	2790859	BYSR-2_peak_160	1.31569	4.17043	2.29387	Chr1:2790393-2790859	2790789	DM992_13120	WP_047900613.1|MULTISPECIES: PrkA family serine protein kinase [Burkholderia cepacia complex]
Merged-Chr1-2772388-2	Chr1:2772222-2772531	promoter	-251	DM992_13050	1&2	BYSR-1_peak_205	1.35988	6.67284	4.19116	Chr1:2772277-2772524	2772381	BYSR-2_peak_159	1.57173	12.8617	8.15907	Chr1:2772222-2772531	2772391	DM992_13050	WP_034184132.1|MULTISPECIES: fimbrial protein [Burkholderia]
Merged-Chr1-2717096-2	Chr1:2716927-2717235	promoter	-156	DM992_12850	1&2	BYSR-1_peak_204	1.44715	8.58592	5.59286	Chr1:2716927-2717203	2717029	BYSR-2_peak_156	1.37209	5.70986	3.39942	Chr1:2717020-2717235	2717077	DM992_12850	WP_105390184.1|hypothetical protein [Burkholderia cepacia]
Merged-Chr1-2642606-2	Chr1:2642132-2643217	promoter	157	DM992_12530	1&2	BYSR-1_peak_197	1.48756	10.2954	6.88031	Chr1:2642147-2642928	2642356	BYSR-2_peak_155	1.51808	10.3877	6.60203	Chr1:2642132-2643217	2642769	DM992_12530	WP_175025334.1|glutathione S-transferase N-terminal domain-containing protein [Burkholderia lata]
Merged-Chr1-2628125-2	Chr1:2627890-2628425	promoter	-17	DM992_12455	1&2	BYSR-1_peak_195	1.40832	7.37852	4.7101	Chr1:2627973-2628214	2628104	BYSR-2_peak_151	1.38802	6.15542	3.71825	Chr1:2627890-2628425	2628148	DM992_12455	WP_105390131.1|MULTISPECIES: universal stress protein [Burkholderia cepacia complex]
Merged-Chr1-2594263-2	Chr1:2593944-2594620	TTS (JP2270_GM002384_rna)	951	DM992_12285	1&2	BYSR-1_peak_194	1.35818	5.93961	3.65415	Chr1:2593944-2594620	2594094	BYSR-2_peak_150	1.32764	4.81831	2.7588	Chr1:2594054-2594437	2594235	DM992_12285	WP_175749454.1|PAS domain-containing protein [Burkholderia pyrrocinia]
Merged-Chr1-2577900-2	Chr1:2577617-2578187	promoter	-83	DM992_12215	1&2	BYSR-1_peak_192	1.64434	15.8233	11.219	Chr1:2577617-2578170	2577974	BYSR-2_peak_149	1.65449	14.5426	8.6917	Chr1:2577628-2578187	2577863	DM992_12215	WP_105393588.1|response regulator transcription factor [Burkholderia cepacia]
Merged-Chr1-2483922-2	Chr1:2483779-2484091	promoter	-14	DM992_11795	1&2	BYSR-1_peak_185	1.22992	2.92025	1.49047	Chr1:2483779-2483994	2483780	BYSR-2_peak_144	1.41971	6.60745	4.04537	Chr1:2483824-2484091	2483959	DM992_11795	WP_060306487.1|recombinase family protein [Burkholderia cepacia]
Merged-Chr1-242046-2	Chr1:241852-242305	promoter	31	DM992_01115	1&2	BYSR-1_peak_20	1.3851	6.32907	3.93686	Chr1:241852-242305	242159	BYSR-2_peak_10	1.22641	2.80739	1.3502	Chr1:241852-242177	241964	DM992_01115	WP_006025184.1|MULTISPECIES: 50S ribosomal protein L21 [Burkholderiaceae]
Merged-Chr1-2372207-2	Chr1:2372032-2372355	promoter	86	DM992_11255	1&2	BYSR-1_peak_181	1.32316	4.7946	2.82373	Chr1:2372089-2372355	2372171	BYSR-2_peak_139	1.52107	9.274	5.87716	Chr1:2372032-2372355	2372149	DM992_11255	WP_004193673.1|MULTISPECIES: 30S ribosomal protein S6 [Burkholderia]

Merged-Chr1-2346301-2	Chr1:2345886-2346820	TTS (JP2270_GM002146_rna)	906	DM992_11115	1&2	BYSR-1_peak_178	1.39121	6.90854	4.36549	Chr1:2345932-2346569	2346211	BYSR-2_peak_138	1.46352	8.0228	5.02054	Chr1:2345886-2346820	2346220	DM992_11115	WP_114181700.1|NADP-dependent oxidoreductase [Burkholderia pyrrocinia]
Merged-Chr1-2337722-2	Chr1:2337381-2338257	promoter-TSS (JP2270_GM002136_rna)	651	DM992_11075	1&2	BYSR-1_peak_176	1.41884	7.52134	4.81366	Chr1:2337460-2337790	2337571	BYSR-2_peak_137	1.4283	6.89422	4.2486	Chr1:2337381-2338257	2337659	DM992_11075	WP_006416008.1|hypothetical protein [Burkholderia multivorans]
Merged-Chr1-2333970-2	Chr1:2333716-2334152	promoter	-252	DM992_11065	1&2	BYSR-1_peak_175	1.24112	3.4219	1.84444	Chr1:2333716-2334077	2333983	BYSR-2_peak_136	1.35674	5.63948	3.35031	Chr1:2333937-2334152	2334015	DM992_11065	WP_059498014.1|acetyl-CoA C-acetyltransferase [Burkholderia vietnamiensis]
Merged-Chr1-2282562-2	Chr1:2282431-2282697	promoter	80	DM992_10850	1&2	BYSR-1_peak_169	1.40762	6.93825	4.38591	Chr1:2282458-2282697	2282622	BYSR-2_peak_134	1.39276	5.8802	3.51757	Chr1:2282431-2282662	2282567	DM992_10850	WP_157776331.1|hypothetical protein [Burkholderia stabilis]
Merged-Chr1-2233750-2	Chr1:2233301-2234228	TTS (JP2270_GM002047_rna)	1163	DM992_10595	1&2	BYSR-1_peak_167	1.45879	8.75662	5.72282	Chr1:2233301-2234228	2233818	BYSR-2_peak_130	1.27203	3.45132	1.79107	Chr1:2233603-2233871	2233633	DM992_10595	WP_175920235.1|3'-5' exonuclease [Burkholderia pyrrocinia]
Merged-Chr1-2226533-3	Chr1:2225603-2227846	promoter	-197	DM992_10555	1&2	BYSR-1_peak_164,BYSR-1_peak_163	-	-	-	-	-	BYSR-2_peak_128	1.59417	12.6031	8.01799	Chr1:2225603-2227846	2226969	DM992_10555	AIO35605.1|putative hedgehog/intein hint domain protein [Burkholderia cenocepacia]
Merged-Chr1-2218520-2	Chr1:2218243-2218751	promoter	-309	DM992_10540	1&2	BYSR-1_peak_161	1.39567	7.21971	4.5935	Chr1:2218397-2218751	2218640	BYSR-2_peak_126	1.30852	4.36516	2.43329	Chr1:2218243-2218691	2218552	DM992_10540	WP_060373856.1|type VI secretion system tip protein VgrG [Burkholderia stagnalis]
Merged-Chr1-2217574-2	Chr1:2217255-2217815	promoter	406	DM992_10535	1&2	BYSR-1_peak_160	1.25379	3.84041	2.14267	Chr1:2217442-2217815	2217485	BYSR-2_peak_125	1.29607	4.27232	2.36798	Chr1:2217255-2217786	2217601	DM992_10535	WP_052747129.1|hypothetical protein [Burkholderia vietnamiensis]
Merged-Chr1-2210525-2	Chr1:2210168-2210948	promoter	-428	DM992_10515	1&2	BYSR-1_peak_156	1.33395	5.61529	3.41704	Chr1:2210168-2210948	2210252	BYSR-2_peak_124	1.39547	6.6323	4.06342	Chr1:2210243-2210743	2210304	DM992_10515	WP_080420531.1|type VI secretion system tip protein VgrG [Burkholderia ubonensis]
Merged-Chr1-2191478-2	Chr1:2191067-2191827	promoter	-218	DM992_10445	1&2	BYSR-1_peak_153	1.3417	6.27598	3.89983	Chr1:2191067-2191827	2191675	BYSR-2_peak_121	1.3852	6.50361	3.96746	Chr1:2191248-2191773	2191623	DM992_10445	KWK69080.1|hypothetical protein WT82_14875 [Burkholderia stagnalis]
Merged-Chr1-2090203-2	Chr1:2089901-2090526	promoter	107	DM992_09940	1&2	BYSR-1_peak_149	1.44415	8.93401	5.85595	Chr1:2089913-2090526	2090071	BYSR-2_peak_118	1.47187	8.80982	5.56051	Chr1:2089901-2090475	2090072	DM992_09940	OXH80921.1|hypothetical protein CA830_42350, partial [Burkholderia multivorans]
Merged-Chr1-2089482-2	Chr1:2089329-2089644	promoter-TSS (JP2270_GM001921_rna)	542	DM992_09945	1&2	BYSR-1_peak_148	1.40785	8.11713	5.24832	Chr1:2089384-2089644	2089529	BYSR-2_peak_117	1.35821	5.70992	3.39942	Chr1:2089329-2089571	2089393	DM992_09945	WP_059695616.1|ShlB/FhaC/HecB family hemolysin secretion/activation protein [Burkholderia cepacia]
Merged-Chr1-2087812-3	Chr1:2086464-2088554	promoter	-245	DM992_09920	1&2	BYSR-1_peak_147, BYSR-1_peak_146	-	-	-	-	-	BYSR-2_peak_116	1.50783	9.51506	6.03984	Chr1:2086464-2088524	2088418	DM992_09920	WP_141684962.1|hypothetical protein [Burkholderia stabilis]
Merged-Chr1-2060010-2	Chr1:2059320-2060609	Intergenic	2419	DM992_09815	1&2	BYSR-1_peak_143	1.53757	11.4981	7.79908	Chr1:2059320-2060609	2060108	BYSR-2_peak_114	1.52501	9.81895	6.24234	Chr1:2059540-2060574	2060244	DM992_09815	WP_175941532.1|hypothetical protein [Burkholderia pyrrocinia]
Merged-Chr1-2058269-2	Chr1:2057783-2058750	TTS (JP2270_GM001900_rna)	721	DM992_09815	1&2	BYSR-1_peak_142	1.28588	4.45038	2.57726	Chr1:2057783-2058750	2057938	BYSR-2_peak_113	1.47344	7.93263	4.96046	Chr1:2057850-2058695	2058551	DM992_09815	WP_175941532.1|hypothetical protein [Burkholderia pyrrocinia]
Merged-Chr1-2000693-2	Chr1:2000455-2000942	promoter	-89	DM992_09545	1&2	BYSR-1_peak_138	1.22671	2.89563	1.47242	Chr1:2000455-2000714	2000539	BYSR-2_peak_112	1.42935	6.84068	4.21031	Chr1:2000661-2000942	2000836	DM992_09545	WP_072436992.1|MULTISPECIES: pyruvate dehydrogenase (acetyl-transferring), homodimeric type [Burkholderia]
Merged-Chr1-1962086-2	Chr1:1961717-1962418	promoter	-75	DM992_09385	1&2	BYSR-1_peak_137	1.26094	3.72242	2.05829	Chr1:1961717-1962418	1962070	BYSR-2_peak_111	1.38423	6.00886	3.61042	Chr1:1961814-1962398	1961983	DM992_09385	WP_080403834.1|hypothetical protein [Burkholderia ubonensis]
Merged-Chr1-1919476-2	Chr1:1919276-1919663	promoter	-202	DM992_09195	1&2	BYSR-1_peak_136	1.39636	6.63029	4.15855	Chr1:1919276-1919663	1919532	BYSR-2_peak_109	1.33495	4.56554	2.57574	Chr1:1919306-1919662	1919549	DM992_09195	WP_096471344.1|toxic anion resistance protein [Burkholderia stabilis]
Merged-Chr1-18895-2	Chr1:18629-19352	promoter	127	DM992_00065	1&2	BYSR-1_peak_2	1.27549	4.4071	2.54822	Chr1:18629-18898	18801	BYSR-2_peak_2	1.32299	4.85652	2.78516	Chr1:18703-19352	19063	DM992_00065	OOA60046.1|hypothetical protein A8F63_37480 [Burkholderia cenocepacia]
Merged-Chr1-1841483-2	Chr1:1841146-1841737	promoter	-232	DM992_08765	1&2	BYSR-1_peak_131	1.63463	15.0927	10.6499	Chr1:1841325-1841737	1841427	BYSR-2_peak_103	1.46472	7.96487	4.98366	Chr1:1841146-1841724	1841394	DM992_08765	WP_072440706.1|MULTISPECIES: carbonic anhydrase [Burkholderia]
Merged-Chr1-1638020-2	Chr1:1637761-1638394	promoter	-181	DM992_07815	1&2	BYSR-1_peak_126	1.32845	5.21996	3.13147	Chr1:1637761-1638097	1637877	BYSR-2_peak_99	1.23305	2.99101	1.4744	Chr1:1637831-1638394	1638142	DM992_07815	WP_034178770.1|MULTISPECIES: EVE domain-containing protein [Burkholderia]
Merged-Chr1-1605759-2	Chr1:1605625-1605889	promoter	255	DM992_07645	1&2	BYSR-1_peak_125	1.3119	4.53818	2.6395	Chr1:1605625-1605889	1605780	BYSR-2_peak_98	1.43122	6.80405	4.18293	Chr1:1605644-1605880	1605801	DM992_07645	WP_034178802.1|MULTISPECIES: acetylornithine transaminase [Burkholderia]
Merged-Chr1-1548684-2	Chr1:1548402-1548918	TTS (JP2270_GM001433_rna)	1466	DM992_07375	1&2	BYSR-1_peak_120	1.36672	6.11656	3.78394	Chr1:1548402-1548774	1548692	BYSR-2_peak_95	1.35417	5.35698	3.14682	Chr1:1548642-1548918	1548877	DM992_07375	WP_059696130.1|MULTISPECIES: UvrD-helicase domain-containing protein [Burkholderia]
Merged-Chr1-1544990-2	Chr1:1544699-1545162	promoter	-195	DM992_07365	1&2	BYSR-1_peak_119	1.35699	6.19848	3.84191	Chr1:1544699-1545162	1544854	BYSR-2_peak_94	1.38211	6.00616	3.61039	Chr1:1544939-1545162	1545054	DM992_07365	WP_151004258.1|helix-turn-helix domain-containing protein [Burkholderia territorii]
Merged-Chr1-1544085-3	Chr1:1543587-1544481	promoter	-1091	DM992_07365	1&2	BYSR-1_peak_118	1.40812	7.39705	4.72394	Chr1:1543587-1544481	1543802	BYSR-2_peak_92, BYSR-2_peak_93	-	-	-	-	-	DM992_07365	WP_151004258.1|helix-turn-helix domain-containing protein [Burkholderia territorii]
Merged-Chr1-1539427-2	Chr1:1539217-1539627	promoter	-847	DM992_07340	1&2	BYSR-1_peak_116	1.27218	4.05076	2.29122	Chr1:1539217-1539483	1539379	BYSR-2_peak_91	1.38875	6.36817	3.87031	Chr1:1539384-1539627	1539529	DM992_07340	WP_043291480.1|MULTISPECIES: hypothetical protein [Burkholderia]
Merged-Chr1-1537337-2	Chr1:1536901-1537663	promoter	353	DM992_07335	1&2	BYSR-1_peak_115	1.3309	5.35817	3.23214	Chr1:1536901-1537663	1537354	BYSR-2_peak_90	1.27378	3.6443	1.92578	Chr1:1537234-1537550	1537293	DM992_07335	WP_059739865.1|type VI secretion system tip protein VgrG [Burkholderia vietnamiensis]
Merged-Chr1-1529959-3	Chr1:1529341-1530644	promoter	57	DM992_07310	1&2	BYSR-1_peak_111, BYSR-1_peak_110	-	-	-	-	-	BYSR-2_peak_86	1.51761	9.7999	6.22825	Chr1:1529341-1530644	1530029	DM992_07310	WP_081064066.1|ATP-binding protein [Burkholderia vietnamiensis]
Merged-Chr1-1516034-3	Chr1:1515461-1516846	promoter	-1999	DM992_07260	1&2	BYSR-1_peak_105	1.3895	7.10698	4.50937	Chr1:1515461-1516846	1516334	BYSR-2_peak_82, BYSR-2_peak_83	-	-	-	-	-	DM992_07260	WP_175751356.1|hypothetical protein [Burkholderia ambifaria]
Merged-Chr1-1512860-2	Chr1:1512653-1513179	promoter	-497	DM992_07255	1&2	BYSR-1_peak_104	1.24735	3.29591	1.75475	Chr1:1512683-1512926	1512720	BYSR-2_peak_80	1.38011	5.66704	3.36744	Chr1:1512653-1513179	1513160	DM992_07255	ABO55627.1|hypothetical protein Bcep1808_2635 [Burkholderia vietnamiensis G4]
Merged-Chr1-1451787-2	Chr1:1451605-1451954	promoter	-174	DM992_06940	1&2	BYSR-1_peak_102	1.37947	6.18099	3.82895	Chr1:1451605-1451954	1451867	BYSR-2_peak_77	1.24134	2.97554	1.46597	Chr1:1451683-1451909	1451687	DM992_06940	WP_174419806.1|MULTISPECIES: purine/pyrimidine permease [Burkholderia cepacia complex]
Merged-Chr1-145164-2	Chr1:144969-145371	promoter	-286	DM992_00640	1&2	BYSR-1_peak_15	1.4414	7.90208	5.09052	Chr1:144990-145327	145140	BYSR-2_peak_9	1.59167	11.4603	7.30227	Chr1:144969-145371	145105	DM992_00640	WP_105389845.1|autotransporter outer membrane beta-barrel domain-containing protein [Burkholderia cepacia]

Merged-Chr1-1258720-4	Chr1:1257128-1261485	exon (JP2270_GM001175_rna, exon 1 of 1)	968	DM992_06050	1&2	BYSR-1_peak_98, BYSR-1_peak_97	-	-	-	-	-	BYSR-2_peak_72, BYSR-2_peak_73	-	-	-	-	-	DM992_06050	WP_081079149.1|ATP-binding protein [Burkholderia territorii]
Merged-Chr1-1253949-2	Chr1:1253789-1254081	promoter	371	DM992_06025	1&2	BYSR-1_peak_94	1.35916	6.11632	3.78394	Chr1:1253853-1254081	1254042	BYSR-2_peak_71	1.29368	4.4361	2.48306	Chr1:1253789-1254073	1253929	DM992_06025	RQZ81300.1|hypothetical protein DF058_34405 [Burkholderia cenocepacia]
Merged-Chr1-1251665-2	Chr1:1251433-1251898	promoter	-190	DM992_06020	1&2	BYSR-1_peak_92	1.36399	6.68215	4.19854	Chr1:1251578-1251898	1251763	BYSR-2_peak_70	1.40375	7.03288	4.3466	Chr1:1251433-1251752	1251566	DM992_06020	WP_124675909.1|D-amino acid dehydrogenase [Burkholderia cenocepacia]
Merged-Chr1-1233174-2	Chr1:1232999-1233361	promoter	114	DM992_05925	1&2	BYSR-1_peak_91	1.37384	6.03462	3.7225	Chr1:1232999-1233238	1233055	BYSR-2_peak_69	1.27077	3.31853	1.6993	Chr1:1233100-1233361	1233122	DM992_05925	WP_006477786.1|MULTISPECIES: ribosome-associated translation inhibitor RaiA [Burkholderia]
Merged-Chr1-1222395-2	Chr1:1222252-1222540	promoter	293	DM992_05865	1&2	BYSR-1_peak_90	1.30409	4.53349	2.63825	Chr1:1222252-1222522	1222458	BYSR-2_peak_67	1.37856	5.71969	3.40324	Chr1:1222266-1222540	1222399	DM992_05865	WP_034179070.1|MULTISPECIES: 50S ribosomal protein L25/general stress protein Ctc [Burkholderia]
Merged-Chr1-1194190-2	Chr1:1193976-1194385	exon (JP2270_GM001118_rna, exon 1 of 1)	1060	DM992_05745	1&2	BYSR-1_peak_88	1.31608	5.15613	3.08709	Chr1:1193976-1194385	1194134	BYSR-2_peak_65	1.28209	3.97635	2.15709	Chr1:1194065-1194337	1194156	DM992_05745	WP_175919432.1|error-prone DNA polymerase [Burkholderia pyrrocinia]
Merged-Chr1-1027510-2	Chr1:1027287-1027725	promoter	-35	DM992_04920	1&2	BYSR-1_peak_84	1.39129	7.35052	4.68954	Chr1:1027287-1027711	1027355	BYSR-2_peak_62	1.33686	5.19578	3.03086	Chr1:1027319-1027725	1027367	DM992_04920	WP_069257750.1|cupredoxin domain-containing protein [Burkholderia metallica]

Supplementary Table S5 The putative binding sequences of BysR in *Burkholderia* sp. JP2-270

**Peak ID**	**Putative binding sequences**	**Location of the binding sequence (Chr: Start-end)**
Merged-Chr1-32620-2	ATTTGCCGCACGAAT	Chr1:32723-32737
Merged-Chr1-32620-2	ATCACATTGAGGAAT	Chr1:32818-32832
Merged-Chr1-32620-2	ATCGTGCGGCGGGAT	Chr1:32741-32755
Merged-Chr1-32620-2	ATTGGCGCATTGGAT	Chr1:32848-32862
Merged-Chr1-32620-2	ATATTCGAAATCCAT	Chr1:32632-32646
Merged-Chr1-387475-2	ATTTCTATCTGAGAT	Chr1:387042-387056
Merged-Chr1-387475-2	ATGGTTGCCTGAAAT	Chr1:386812-386826
Merged-Chr1-401162-2	ATTCAAACCCCTCAT	Chr1:401145-401159
Merged-Chr1-401162-2	ATGCAGCTCGTTGAT	Chr1:401591-401605
Merged-Chr1-401162-2	ATGCGCTTGCAATAT	Chr1:401543-401557
Merged-Chr1-567795-2	ATCGATATGAGCAAT	Chr1:568030-568044
Merged-Chr1-567795-2	ATCACCATCATGCAT	Chr1:567875-567889
Merged-Chr1-567795-2	ATTCGCCGTGTGAAT	Chr1:567932-567946
Merged-Chr1-927507-2	ATCAACAATCGGAAT	Chr1:927371-927385
Merged-Chr1-927507-2	ATTTGATTCTTCAAT	Chr1:927700-927714
Merged-Chr1-927507-2	ATCGCCCTCGCTGAT	Chr1:927669-927683
Merged-Chr1-927507-2	ATCCATTGCCTCGAT	Chr1:927488-927502
Merged-Chr1-927507-2	ATCGAATCCCTGAAT	Chr1:927908-927922
Merged-Chr1-928808-2	ATCGCTGTTGATGAT	Chr1:929061-929075
Merged-Chr1-928808-2	ATATGACAGAGCCAT	Chr1:928683-928697
Merged-Chr1-928808-2	ATCGAACGATCGGAT	Chr1:928899-928913
Merged-Chr1-928808-2	ATGTCGCACGCCGAT	Chr1:929038-929052
Merged-Chr1-958146-2	ATCCGCCCCTGGAAT	Chr1:958315-958329
Merged-Chr1-958146-2	ATGTCCGCCTGACAT	Chr1:958514-958528
Merged-Chr1-958146-2	ATTTGGATACGTCAT	Chr1:958430-958444
Merged-Chr1-1529959-3	ATGCAAGAAGCGGAT	Chr1:1530059-1530073
Merged-Chr1-1529959-3	ATCCGCGATGTCAAT	Chr1:1529895-1529909
Merged-Chr1-1529959-3	ATATCCGCGAACAAT	Chr1:1530372-1530386
Merged-Chr1-1529959-3	ATTCCGCGCTTGTAT	Chr1:1529412-1529426
Merged-Chr1-1544085-3	ATGACATGCAGATAT	Chr1:1543906-1543920
Merged-Chr1-1544085-3	ATTCTGTCCAACAAT	Chr1:1543687-1543701
Merged-Chr1-1544085-3	ATAGAACCGCCTGAT	Chr1:1544431-1544445
Merged-Chr1-1638020-2	ATTCCTTGAACCAAT	Chr1:1637996-1638010
Merged-Chr1-1638020-2	ATGCCAACAGACCAT	Chr1:1638215-1638229
Merged-Chr1-1841483-2	ATCCTATTGCGCCAT	Chr1:1841600-1841614
Merged-Chr1-1841483-2	ATTAATCCAGGAAAT	Chr1:1841357-1841371
Merged-Chr1-1841483-2	ATTTGCCTTTGTCAT	Chr1:1841629-1841643
Merged-Chr1-1962086-2	ATGATCGTTCGCTAT	Chr1:1962091-1962105
Merged-Chr1-2090203-2	ATTCACAAATCAAAT	Chr1:2090362-2090376
Merged-Chr1-2090203-2	ATCCTTAATCCACAT	Chr1:2090202-2090216
Merged-Chr1-2090203-2	ATAGACAACAAACAT	Chr1:2089912-2089926
Merged-Chr1-2090203-2	ATTGATTCCATACAT	Chr1:2090042-2090056
Merged-Chr1-2090203-2	ATCATCAATCCGCAT	Chr1:2089949-2089963
Merged-Chr1-2191478-2	ATTGACATCGAGAAT	Chr1:2191578-2191592
Merged-Chr1-2191478-2	ATTTTCTCGTGAAAT	Chr1:2191440-2191454
Merged-Chr1-2210525-2	ATAATTTCGCGCGAT	Chr1:2210421-2210435
Merged-Chr1-2210525-2	ATACCACCGTGACAT	Chr1:2210276-2210290
Merged-Chr1-2210525-2	ATTCTTCCGCTTGAT	Chr1:2210864-2210878
Merged-Chr1-2218520-2	ATTACATCGAGAGAT	Chr1:2218296-2218310
Merged-Chr1-2577900-2	ATCCCTGTAAGCAAT	Chr1:2577724-2577738

Merged-Chr1-2577900-2	ATGAATTGATAGCAT	Chr1:2577968-2577982
Merged-Chr1-2577900-2	ATCCGACCGTCAAAT	Chr1:2577647-2577661
Merged-Chr1-2577900-2	ATCTAGTAGGAATAT	Chr1:2577941-2577955
Merged-Chr1-2628125-2	ATCCATTTTGTCGAT	Chr1:2627986-2628000
Merged-Chr1-2841953-2	ATTGCATGTTTAAAT	Chr1:2841740-2841754
Merged-Chr1-2879508-2	ATTAGGTGTGTACAT	Chr1:2879429-2879443
Merged-Chr1-2879508-2	ATATGCGGGGTGTAT	Chr1:2879746-2879760
Merged-Chr1-2921049-2	ATGTTTCTTGAGGAT	Chr1:2921098-2921112
Merged-Chr1-2921049-2	ATGGCCCAACTGGAT	Chr1:2920933-2920947
Merged-Chr1-2921049-2	ATTGAACCCGGCGAT	Chr1:2921020-2921034
Merged-Chr1-2921049-2	ATGGGTATCGTCCAT	Chr1:2921230-2921244
Merged-Chr1-2921049-2	ATAATCTCGGCCGAT	Chr1:2920723-2920737
Merged-Chr1-3377413-2	ATACAAATTGAATAT	Chr1:3377112-3377126
Merged-Chr1-3684201-2	ATATCGCAACTGTAT	Chr1: 3684338- 3684352
Merged-Chr1-3684201-2	ATTCGCCGCCACGAT	Chr1: 3684209- 3684223
Merged-Chr1-3684201-2	ATCGCCGCGCGGAAT	Chr1: 3684067- 3684081
Merged-Chr2-8872-2	ATTTGCCGCGAGTAT	Chr2:8843-8857
Merged-Chr2-8872-2	ATTTTTGTTTATAAT	Chr2:9024-9038
Merged-Chr2-8872-2	ATTGATATTACTGAT	Chr2:8957-8971
Merged-Chr2-8872-2	ATCGAATTAGACGAT	Chr2:9094-9108
Merged-Chr2-8872-2	ATTTGTTGAGCAGAT	Chr2:8531-8545
Merged-Chr2-8872-2	ATCGGAACGATGCAT	Chr2:8912-8926
Merged-Chr2-114880-3	ATCGTCTGGTTCCAT	Chr2:115320-115334
Merged-Chr2-268951-2	ATGAAAAGAAGAGAT	Chr2:268346-268360
Merged-Chr2-268951-2	ATTGAAATTGGGAAT	Chr2:268957-268971
Merged-Chr2-268951-2	ATCATGGAAGGAAAT	Chr2:269507-269521
Merged-Chr2-268951-2	ATCGTTGGCCTGGAT	Chr2:268748-268762
Merged-Chr2-268951-2	ATCAAGGCAATGAAT	Chr2:269348-269362
Merged-Chr2-268951-2	ATTATCGTCAACGAT	Chr2:269003-269017
Merged-Chr2-268951-2	ATGTACTTTCGGAAT	Chr2:268632-268646
Merged-Chr2-268951-2	ATGGCACAAGGGCAT	Chr2:268409-268423
Merged-Chr2-268951-2	ATCAAACACAATAAT	Chr2:269687-269701
Merged-Chr2-444433-2	ATAGGAGTATTGCAT	Chr2:444137-444151
Merged-Chr2-444433-2	ATCGTCGCAGACGAT	Chr2:444056-444070
Merged-Chr2-513065-2	ATTCGGCGCTCACAT	Chr2:513205-513219
Merged-Chr2-513065-2	ATGACCATCCGTCAT	Chr2:513275-513289
Merged-Chr2-513065-2	ATTAATCATTCCAAT	Chr2:513134-513148
Merged-Chr2-989182-2	ATTGTGGGCCCTGAT	Chr2:989241-989255
Merged-Chr2-989182-2	ATCGAAGCCGAATAT	Chr2:989111-989125
Merged-Chr2-989182-2	ATGCCGACGTCGCAT	Chr2:989434-989448
Merged-Chr2-989182-2	ATCCAAGACGGAAAT	Chr2:988974-988988
Merged-Chr2-989182-2	ATATGTATCGGCGAT	Chr2:989052-989066
Merged-Chr2-1054247-2	ATCGAGACGCAATAT	Chr2:1054110-1054124
Merged-Chr2-1058344-2	ATTTGTTGCTGGAAT	Chr2:1058407-1058421
Merged-Chr2-1058344-2	ATTGACGTACGATAT	Chr2:1058466-1058480
Merged-Chr2-1076518-2	ATTATGGCGGAAGAT	Chr2:1076501-1076515
Merged-Chr2-1076518-2	ATCACGAGTGGTTAT	Chr2:1076037-1076051
Merged-Chr2-1076518-2	ATGAGTATGTTAGAT	Chr2:1075502-1075516
Merged-Chr2-1076518-2	ATTATATTGCGTAAT	Chr2:1075367-1075381
Merged-Chr2-1076518-2	ATGCATCGTGTCCAT	Chr2:1075440-1075454
Merged-Chr2-1076518-2	ATGACAAGATGAAAT	Chr2:1076022-1076036
Merged-Chr2-1076518-2	ATCAGATAAAATCAT	Chr2:1076298-1076312
Merged-Chr2-1076518-2	ATGATGAGTCAATAT	Chr2:1075614-1075628
Merged-Chr2-1076518-2	ATCGAGCCAACTTAT	Chr2:1076350-1076364

Merged-Chr2-1076518-2	ATGCGGCGAGGTTAT	Chr2:1076150-1076164
Merged-Chr2-1135463-2	ATAAATGCGGCCAAT	Chr2:1135646-1135660
Merged-Chr2-1135463-2	ATTGACATGATGCAT	Chr2:1135329-1135343
Merged-Chr2-1135463-2	ATCCGCAGACAATAT	Chr2:1135574-1135588
Merged-Chr2-1135463-2	ATACTACTCCACGAT	Chr2:1135487-1135501
Merged-Chr2-1136261-2	ATACCTTACCACCAT	Chr2:1136097-1136111
Merged-Chr2-1136261-2	ATAGCGTCGATTAAT	Chr2:1136051-1136065
Merged-Chr2-1136261-2	ATAGTTCACGGCGAT	Chr2:1136215-1136229
Merged-Chr2-1466418-2	ATGAATTGCGGCCAT	Chr2:1466154-1466168
Merged-Chr2-1466418-2	ATGTTTTCGCCGAAT	Chr2:1466405-1466419
Merged-Chr2-1466418-2	ATGTGCGTGCCCGAT	Chr2:1466137-1466151
Merged-Chr2-1466418-2	ATATTTGTATTTCAT	Chr2:1466224-1466238
Merged-Chr2-1466418-2	ATGTCGACAACCGAT	Chr2:1466591-1466605
Merged-Chr2-1466418-2	ATGACCCACGCGCAT	Chr2:1466439-1466453
Merged-Chr2-1664092-3	ATATGGTCGAGGTAT	Chr2:1663985-1663999
Merged-Chr2-1664092-3	ATCACCATCGGCTAT	Chr2:1664559-1664573
Merged-Chr2-1664092-3	ATTCTTGTGAGATAT	Chr2:1663881-1663895
Merged-Chr2-1664092-3	ATTTCGGCGCTGGAT	Chr2:1664398-1664412
Merged-Chr2-1702104-2	ATTGATACGTCCCAT	Chr2:1701977-1701991
Merged-Chr2-1702104-2	ATTGCCGATTTCGAT	Chr2:1701816-1701830
Merged-Chr2-1702104-2	ATGGCGGGGATTGAT	Chr2:1702189-1702203
Merged-Chr2-1757028-2	ATAGTAATTATCTAT	Chr2:1756796-1756810
Merged-Chr2-1757028-2	ATTGGATAATATAAT	Chr2:1757319-1757333
Merged-Chr2-1757028-2	ATTCTAAAAATTCAT	Chr2:1757003-1757017
Merged-Chr2-1833613-2	ATGATCGCGAACAAT	Chr2:1833307-1833321
Merged-Chr2-1930007-2	ATTCAAAGAGGAAAT	Chr2:1930118-1930132
Merged-Chr2-1930007-2	ATACTGCGACCCAAT	Chr2:1930140-1930154
Merged-Chr2-1930007-2	ATAGCAACGGCGAAT	Chr2:1929794-1929808
Merged-Chr2-1930007-2	ATCGATACACGTAAT	Chr2:1929906-1929920
Merged-Chr2-1930007-2	ATATCGGCTGCTTAT	Chr2:1930338-1930352
Merged-Chr2-1930007-2	ATTCTGCGCCAGCAT	Chr2:1930201-1930215
Merged-Chr2-1930007-2	ATCGACTGCGGTGAT	Chr2:1930075-1930089
Merged-Chr2-2075107-2	ATGAAGTTGACGCAT	Chr2:2075101-2075115
Merged-Chr2-2075107-2	ATTCCGTCTGCGCAT	Chr2:2075447-2075461
Merged-Chr2-2145475-2	ATTCGCTGATTTGAT	Chr2:2145684-2145698
Merged-Chr2-2225394-2	ATGGCTCCGGAACAT	Chr2:2225430-2225444
Merged-Chr2-2225394-2	ATCGCGCAAACGTAT	Chr2:2225618-2225632
Merged-Chr2-2225394-2	ATTTTAGACGAAAAT	Chr2:2225384-2225398
Merged-Chr2-2225394-2	ATTATCGGACGATAT	Chr2:2225407-2225421
Merged-Chr2-2230969-2	ATGGCAATCCGAAAT	Chr2:2231106-2231120
Merged-Chr2-2230969-2	ATCCGTTCCGAAAAT	Chr2:2230858-2230872
Merged-Chr2-2230969-2	ATGTCAGACAAAAAT	Chr2:2231004-2231018
Merged-Chr2-2230969-2	ATTGCGATGAGAAAT	Chr2:2230877-2230891
Merged-Chr2-2230969-2	ATATGCAAAATAAAT	Chr2:2230910-2230924
Merged-Chr2-2392122-2	ATTCGGAACGTGAAT	Chr2:2392285-2392299
Merged-Chr2-2392122-2	ATCGCGACGACGAAT	Chr2:2392447-2392461
Merged-Chr2-2477224-2	ATGCATGAAACAGAT	Chr2:2477078-2477092
Merged-Chr2-2477224-2	ATCGTGCCGATCAAT	Chr2:2477045-2477059
Merged-Chr2-2477224-2	ATGACATTCACCGAT	Chr2:2477011-2477025
Merged-Chr2-2508335-2	ATATGTCACATCGAT	Chr2:2508128-2508142
Merged-Chr2-2508335-2	ATCATACTCCAGAAT	Chr2:2508530-2508544
Merged-Chr2-2508335-2	ATCGGATCGGGCAAT	Chr2:2508658-2508672
Merged-Chr2-2508335-2	ATACGAATCGTCAAT	Chr2:2507906-2507920
Merged-Chr2-2508335-2	ATTTCTTGCACGCAT	Chr2:2508458-2508472

Merged-Chr2-2508335-2	ATTCTTGAGATACAT	Chr2:2508026-2508040
Merged-Chr2-2535564-2	ATTAATCACTCACAT	Chr2:2535717-2535731
Merged-Chr2-2535564-2	ATTCCTAATCGATAT	Chr2:2535821-2535835
Merged-Chr2-2535564-2	ATATAAATGACACAT	Chr2:2535738-2535752
Merged-Chr2-2535564-2	ATGCGCACCCGCAAT	Chr2:2535311-2535325
Merged-Chr2-2679999-2	ATTTGTTTATGTAAT	Chr2:2680112-2680126
Merged-Chr2-2679999-2	ATTCAGGAGTCTCAT	Chr2:2679869-2679883
Merged-Chr2-2679999-2	ATGTTGTGTCTAAAT	Chr2:2680333-2680347
Merged-Chr2-2679999-2	ATTGAAAATCTCCAT	Chr2:2679885-2679899
Merged-Chr2-2679999-2	ATCCACTGCATCCAT	Chr2:2679984-2679998
Merged-Chr2-2679999-2	ATACCGCTACCATAT	Chr2:2680134-2680148
Merged-Chr2-2947117-2	ATGCAGCCAATGGAT	Chr2:2946887-2946901
Merged-Chr2-2947117-2	ATATTTTCACATAAT	Chr2:2947343-2947357
Merged-Chr2-2960541-2	ATCATGGTCACCGAT	Chr2:2960758-2960772
Merged-Chr2-2960541-2	ATTGCTTGATGCAAT	Chr2:2960298-2960312
Merged-Chr2-3092336-2	ATTGGCGAGAGATAT	Chr2:3092477-3092491
Merged-Chr2-3092336-2	ATGGCGCCAGGCCAT	Chr2:3092314-3092328
Merged-Chr2-3092336-2	ATCGTGTTGAGCGAT	Chr2:3092291-3092305
Merged-Chr2-3092336-2	ATCTTTCCCATCCAT	Chr2:3091860-3091874
Merged-Chr2-3129612-3	ATAGAGCGCAAACAT	Chr2:3129584-3129598
Merged-Chr2-3129612-3	ATCGAGCAGCACGAT	Chr2:3129530-3129544
Merged-Chr2-3130812-2	ATCATCAGCGTGCAT	Chr2:3130796-3130810
Merged-Chr3-34745-2	ATTTTTGCCATTGAT	Chr3:34967-34981
Merged-Chr3-34745-2	ATCGATCTGAACCAT	Chr3:34850-34864
Merged-Chr3-34745-2	ATCACCCCCTCGCAT	Chr3:34735-34749
Merged-Chr3-34745-2	ATTTTTCGTCGATAT	Chr3:34715-34729
Merged-Chr3-34745-2	ATAACTTTTTATTAT	Chr3:34644-34658
Merged-Chr3-96974-2	ATGCGCGAAGCGCAT	Chr3:96723-96737
Merged-Chr3-148813-2	ATTGTGCTTTTCCAT	Chr3:148836-148850
Merged-Chr3-148813-2	ATTCAATATGGATAT	Chr3:148684-148698
Merged-Chr3-148813-2	ATTGCGCGTGCATAT	Chr3:148533-148547
Merged-Chr3-148813-2	ATGAAAATCGCCGAT	Chr3:148938-148952
Merged-Chr3-148813-2	ATTTTTTCGTTGAAT	Chr3:149232-149246
Merged-Chr3-148813-2	ATTCGTGGATAAAAT	Chr3:149183-149197
Merged-Chr3-161268-2	ATAAAAAATCGCGAT	Chr3:161394-161408
Merged-Chr3-161268-2	ATTTACCTTTGTAAT	Chr3:161306-161320
Merged-Chr3-161268-2	ATCGCTGCGCCGGAT	Chr3:161561-161575
Merged-Chr3-161268-2	ATATGGAATATTAAT	Chr3:161525-161539
Merged-Chr3-161268-2	ATATCGTTCGACGAT	Chr3:161509-161523
Merged-Chr3-161268-2	ATCATGTCGAGGTAT	Chr3:160903-160917
Merged-Chr3-301729-2	ATTCGATCGATTAAT	Chr3:301956-301970
Merged-Chr3-301729-2	ATGGCCGGTCAGAAT	Chr3:301560-301574
Merged-Chr3-301729-2	ATTAACTTTTAATAT	Chr3:301405-301419
Merged-Chr3-301729-2	ATTGAATGATGGAAT	Chr3:301787-301801
Merged-Chr3-301729-2	ATTGAATGCACTGAT	Chr3:301516-301530
Merged-Chr3-301729-2	ATATATAAGATAAAT	Chr3:301882-301896
Merged-Chr3-423602-2	ATTGAAGGTGACGAT	Chr3:423440-423454
Merged-Chr3-435219-2	ATGGAAACGTTGAAT	Chr3:435082-435096
Merged-Chr3-435219-2	ATGCGCATAAGGAAT	Chr3:435055-435069
Merged-Chr3-439543-2	ATGCAGACATTTTAT	Chr3:439227-439241
Merged-Chr3-439543-2	ATCCCGCGTCTGAAT	Chr3:439747-439761
Merged-Chr3-439543-2	ATGCCAACTGCCGAT	Chr3:439729-439743
Merged-Chr3-439543-2	ATCGTGCAGTTGAAT	Chr3:439621-439635
Merged-Chr3-439543-2	ATTTCGACCGGGCAT	Chr3:439817-439831

Merged-Chr3-439543-2	ATCGCGTGCCGAAAT	Chr3:439710-439724
Merged-Chr3-484876-2	ATTGCTTGTATTCAT	Chr3:484892-484906
Merged-Chr3-484876-2	ATCGTCTATGCCCAT	Chr3:485025-485039
Merged-Chr3-497297-2	ATGGGCGAAAACAAT	Chr3:497427-497441
Merged-Chr3-497297-2	ATGCCTGGTCGGTAT	Chr3:497171-497185
Merged-Chr3-497297-2	ATTTTCATTCCAGAT	Chr3:497269-497283
Merged-Chr3-548297-2	ATTGGTGTCGATGAT	Chr3:548227-548241
Merged-Chr3-548297-2	ATCGTGAGTAGTGAT	Chr3:548143-548157
Merged-Chr3-548297-2	ATGTATTAAGCAGAT	Chr3:548578-548592
Merged-Chr3-551554-3	ATTCCGTGACCTCAT	Chr3:552115-552129
Merged-Chr3-551554-3	ATTTCCCTTTTTTAT	Chr3:551424-551438
Merged-Chr3-551554-3	ATGGTGTCTCTCTAT	Chr3:550963-550977
Merged-Chr3-551554-3	ATGGGATAACCAAAT	Chr3:551086-551100
Merged-Chr3-619701-2	ATATCAGTTAGCGAT	Chr3:619280-619294
Merged-Chr3-619701-2	ATTTCCATTGACCAT	Chr3:619634-619648
Merged-Chr3-619701-2	ATTCGCGACGCACAT	Chr3:619787-619801
Merged-Chr3-619701-2	ATCGGAAAGATGAAT	Chr3:619860-619874
Merged-Chr3-619701-2	ATAACAGCACCTGAT	Chr3:619376-619390
Merged-Chr3-798380-3	ATTGTAAGTCCACAT	Chr3:798732-798746
Merged-Chr3-798380-3	ATCGAAGACCCAGAT	Chr3:797885-797899
Merged-Chr3-798380-3	ATAAATCAATTCGAT	Chr3:798480-798494
Merged-Chr3-798380-3	ATTGAACGAATGGAT	Chr3:797900-797914
Merged-Chr3-798380-3	ATCCACGAGCGTCAT	Chr3:798255-798269
Merged-Chr3-802744-2	ATCTTCAAGAAACAT	Chr3:802840-802854
Merged-Chr3-802744-2	ATTCGGAAACGTTAT	Chr3:802708-802722
Merged-Chr3-802744-2	ATGGTATGAATATAT	Chr3:802752-802766
Merged-Chr3-802744-2	ATATAATCACCAAAT	Chr3:802784-802798
Merged-Chr3-802744-2	ATGGAATGTTCGTAT	Chr3:802513-802527
Merged-Chr3-802744-2	ATAATGCAGGTCGAT	Chr3:802821-802835
Merged-Chr3-802744-2	ATTTAGTGGGCACAT	Chr3:802641-802655
Merged-Chr3-802744-2	ATTTCCTGCGACGAT	Chr3:802616-802630
Merged-Chr3-802744-2	ATCCCGAACGATCAT	Chr3:802461-802475
Merged-Chr3-835916-2	ATCCACCCTGTAGAT	Chr3:835744-835758
Merged-Chr3-835916-2	ATGCAGCAAAAATAT	Chr3:836035-836049
Merged-Chr3-835916-2	ATCGTGTCGGCGCAT	Chr3:835677-835691
Merged-Chr3-835916-2	ATGCGTGGCGCGCAT	Chr3:836197-836211
Merged-Chr3-861222-2	ATGGAAAACAGATAT	Chr3:861452-861466
Merged-Chr3-861222-2	ATTCAGTCGATAAAT	Chr3:861202-861216
Merged-Chr3-861222-2	ATTCACTGCGGGCAT	Chr3:861067-861081
Merged-Chr3-861222-2	ATGCATCAATCCGAT	Chr3:861343-861357
Merged-Chr3-861222-2	ATTAACGCAACGCAT	Chr3:861146-861160
Merged-Chr3-944987-2	ATTTCCGAAATTGAT	Chr3:944923-944937
Merged-Chr3-944987-2	ATCCACGGCAATAAT	Chr3:945298-945312
Merged-Chr3-944987-2	ATCGCGACTCAAAAT	Chr3:944880-944894
Merged-Chr3-944987-2	ATCTTTTCATCGAAT	Chr3:944691-944705
Merged-Chr3-944987-2	ATTTACTCGGCATAT	Chr3:945262-945276
Merged-Chr3-944987-2	ATATTCATCGCCGAT	Chr3:944708-944722
Merged-Chr3-1039738-2	ATCCAAGATGCAGAT	Chr3:1040289-1040303
Merged-Chr3-1039738-2	ATGGACGGATTCCAT	Chr3:1039970-1039984
Merged-Chr3-1039738-2	ATCAACCGGGGAAAT	Chr3:1039486-1039500
Merged-Chr3-1140226-2	ATTTGCCATCGCCAT	Chr3:1140496-1140510
Merged-Chr3-1151532-2	ATTTACCGATTTGAT	Chr3:1151630-1151644
Merged-Chr3-1151532-2	ATTTTTTATTTCAAT	Chr3:1151646-1151660
Merged-Chr3-1196017-2	ATTGGCGACGGTCAT	Chr3:1195728-1195742

Merged-Chr3-1196017-2	ATAAAATTATCGAAT	Chr3:1196377-1196391
Merged-Chr3-1196017-2	ATTCCACACGATAAT	Chr3:1195907-1195921
Merged-Chr3-1196017-2	ATTGTCGCGGCCGAT	Chr3:1196221-1196235
Merged-Chr3-1196017-2	ATTGTGACGCCGTAT	Chr3:1195702-1195716
Merged-Chr3-1196017-2	ATCAACGTAGAGTAT	Chr3:1196543-1196557
Merged-Chr3-1196017-2	ATACGTTATACGTAT	Chr3:1196135-1196149
Merged-Chr3-1362826-2	ATGAGATTATTCAAT	Chr3:1362754-1362768
Merged-Chr3-1454489-2	ATTCTGCTGGTCGAT	Chr3:1454386-1454400
Merged-Chr3-1454489-2	ATCCGACATCAATAT	Chr3:1454334-1454348
Merged-Chr3-1454489-2	ATACGACTCCTAGAT	Chr3:1454778-1454792
Merged-Chr3-1454489-2	ATGAGGTTCTTCGAT	Chr3:1454068-1454082
Merged-Chr3-1459497-2	ATCGATCCGTTGCAT	Chr3:1459199-1459213
Merged-Plas2-27858-2	ATATTCGAGCTTCAT	Plas2:27584-27598
Merged-Plas2-27858-2	ATCGGAACGATAGAT	Plas2:27919-27933
Merged-Plas2-27858-2	ATGAATCAATGGGAT	Plas2:27470-27484
Merged-Plas2-27858-2	ATGCCTTCCGTCAAT	Plas2:27774-27788
Merged-Plas2-27858-2	ATGACAAGCTGCCAT	Plas2:27515-27529
Merged-Plas2-40729-2	ATGTAGATATCGCAT	Plas2:40506-40520
Merged-Plas2-44297-3	ATCGGTCGAGCTGAT	Plas2:44257-44271
Merged-Plas2-44297-3	ATCAAGGCGGCCAAT	Plas2:44790-44804
Merged-Plas2-44297-3	ATGTAGTGGTCTAAT	Plas2:43861-43875
Merged-Plas2-44297-3	ATGTCCGCGCCACAT	Plas2:45118-45132
Merged-Plas2-44297-3	ATGCAAGCCGGAAAT	Plas2:44805-44819
Merged-Plas2-44297-3	ATCTCAATCGCGAAT	Plas2:44559-44573
Merged-Plas2-111794-2	ATTCGTACTACAGAT	Plas2:111853-111867
Merged-Plas2-111794-2	ATTGCTCACCTGCAT	Plas2:112006-112020
Merged-Plas2-241445-2	ATGGAATTCAGCAAT	Plas2:241468-241482
Merged-Plas2-241445-2	ATCCTCGTCCGACAT	Plas2:241354-241368
Merged-Plas2-246094-2	ATCGAATGCTCGGAT	Plas2:246420-246434
Merged-Plas2-246094-2	ATACGAGTATTCGAT	Plas2:246176-246190
Merged-Plas2-246094-2	ATAACCGGTTGACAT	Plas2:245773-245787
Merged-Plas2-290078-2	ATCCGTTGAGGCGAT	Plas2:290093-290107
Merged-Plas2-290078-2	ATCAAGTTATCTCAT	Plas2:290259-290273
Merged-Plas2-290078-2	ATCGACTCACGCCAT	Plas2:290026-290040
Merged-Plas2-305067-2	ATCCGCAACCCGGAT	Plas2:305012-305026
Merged-Plas2-343011-2	ATCCACGACACAGAT	Plas2:342599-342613
Merged-Plas2-343011-2	ATCCGCCGTTTGAAT	Plas2:343480-343494
Merged-Plas2-343011-2	ATTGAGACACGCTAT	Plas2:343032-343046
Merged-Plas2-343011-2	ATTGCGCTCACCAAT	Plas2:342780-342794
Merged-Plas2-343011-2	ATCAATCCGCAAGAT	Plas2:342756-342770
Merged-Plas2-343011-2	ATTTAAACCTCACAT	Plas2:343347-343361
Merged-Plas2-343011-2	ATTGAACCCCTGGAT	Plas2:343197-343211
Merged-Plas2-343011-2	ATTCGATGGCATGAT	Plas2:343592-343606
Merged-Plas2-343011-2	ATTAGAGTTAGAAAT	Plas2:343364-343378
Merged-Plas2-363376-3	ATCAATTTCGTTCAT	Plas2:363795-363809
Merged-Plas2-363376-3	ATCCTTGACCGTGAT	Plas2:363172-363186
Merged-Plas2-363376-3	ATCAAGCCTGAACAT	Plas2:362875-362889
Merged-Plas2-363376-3	ATCGCAGTTCACCAT	Plas2:364426-364440
Merged-Plas2-363376-3	ATGAGTAACGATGAT	Plas2:363895-363909
Merged-Plas2-363376-3	ATAGATGGCGGGTAT	Plas2:363766-363780
Merged-Plas2-385235-2	ATTCCGAAACGGGAT	Plas2:385019-385033
Merged-Plas2-385235-2	ATTCGACAAGGTCAT	Plas2:385061-385075
Merged-Plas2-385235-2	ATCCGCTTCTGGCAT	Plas2:385489-385503
